# Red Wine Oxidation: Accelerated Ageing Tests, Possible Reaction Mechanisms and Application to Syrah Red Wines

**DOI:** 10.3390/antiox9080663

**Published:** 2020-07-24

**Authors:** Stacy Deshaies, Guillaume Cazals, Christine Enjalbal, Thibaut Constantin, François Garcia, Laetitia Mouls, Cédric Saucier

**Affiliations:** 1 SPO, Université de Montpellier, INRAE, Institut Agro, 34000 Montpellier, France; stacy.deshaies@umontpellier.fr (S.D.); francois.garcia@umontpellier.fr (F.G.); laetitia.mouls@supagro.fr (L.M.); 2 IBMM, Université de Montpellier, 34093 Montpellier, France; guillaume.cazals@umontpellier.fr (G.C.); christine.enjalbal@umontpellier.fr (C.E.); 3 Laboratoire d’Œnologie, UFR Pharmacie, Université de Montpellier, 34000 Montpellier, France; thibaut.constantin@umontpellier.fr

**Keywords:** wine, oxidation, polyphenol, Syrah, mass spectrometry, oxygen, vintage, markers

## Abstract

Wine oxidation and ageing involve many complex chemical pathways and reaction mechanisms. The purpose of this study is to set up new and reproducible accelerated red wine ageing tests and identify chemical oxidation or ageing molecular markers. Three accelerated and reproducible ageing tests were developed: a heat test (60 °C); an enzymatic test (laccase test; a chemical test (hydrogen peroxide test). Depending on the test, oxygen consumption was significantly different. For a young wine (2018), the oxygen consumption rate moved from 2.40 ppm·h^−1^ for the heat test to 3.33 ppm·h^−1^ for the enzymatic test and 2.86 ppm·h^−1^ for the chemical test. Once applied to two other vintages (2010 and 2014) from the same winery, the tests revealed different comportments corresponding to wine natural evolution. High resolution UPLC-MS was performed on forced ageing samples and compared to naturally aged red wines. Specific oxidation or ageing ion markers were found with significant differences between tests, revealing the specificity of each test and different possible molecular pathways involved. The hydrogen peroxide test seems to be closer to natural oxidation with an important decrease in absorbance at 520 nm and similar molecular ion variations for [M+H]^+^ = 291, 331, 347, 493, 535, 581, 639 Da.

## 1. Introduction

Oxygen has an important role in chemical reactions in red wines from the winemaking process to bottle ageing. Oxidation reactions have an impact on the chemical and sensory characteristics, such as wine color [1,2,3] or organoleptic properties [4,5].

An optimal red wine quality is correlated with a moderate oxygen exposure during the whole wine lifetime [3]. One example is its consumption by yeasts to produce sterols during alcoholic fermentation. This allows them to have better alcohol resistance and nitrogen nutrient absorption. From an organoleptic point of view, an optimal oxygen exposure may reduce some wine negative aspects, such as bitterness or astringency [3,6]. Unwanted aroma or color instability may also occur in the case of too high or too low oxygen exposures [7]. For red wines, a too high oxygen exposure will reduce antioxidant concentrations (sulfur dioxide or ascorbic acid) and desirable volatile compounds, affecting wine quality [8].

Managing oxygen exposure then is a real challenge for winemakers. Each step of the winemaking process involves a specific oxygen level exposure, provided in continuous diffusion manner or at a given moment. This oxygen transfer occurs during different winemaking steps, such as barrel aging (exchange through or with the barrel wood), micro-oxygenation or even bottle storage, which is particularly important for the improvement of red wine quality [9]. As the wine composition is closely related to its ability to react with oxygen, it is often difficult to predict the outcomes for a specific wine and a given oxygen amount, without mentioning accidental and uncontrolled oxygen exposure. Even if some tools were developed to precisely control the dissolved oxygen levels in wines, there is a lack of techniques to predict the wine evolution depending on its oxygen exposure and its aging capacity in bottles.

**Scheme 1:**
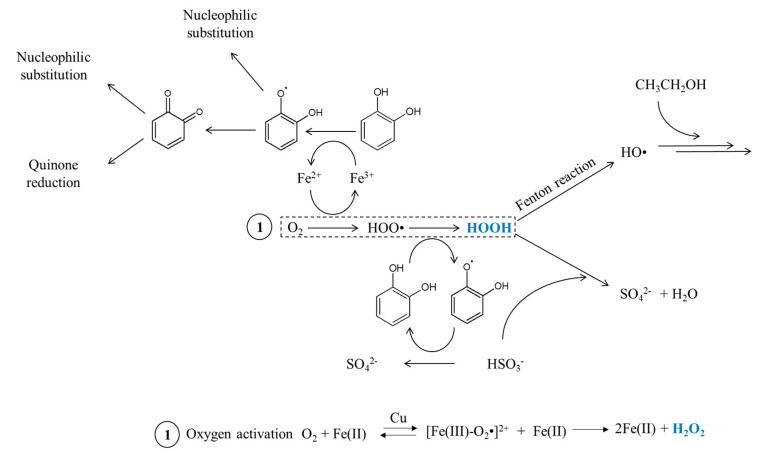
Partial iron-catalyzed wine oxidation scheme.

From a chemical point of view, polyphenols are among the most readily oxidized wine constituents [8,10]. Chemical oxidation reactions occur in red wines, and involve polyphenols, such as anthocyanins, proanthocyanidins and flavan-3-ols [11,12]. Enzymatic oxidation can also occur in botrytized wines containing laccases, which are polyphenol oxidase enzymes [13]. Polyphenols containing a galloyl or catechol group will be oxidized in *ortho*-quinones, which are very reactive and electrophilic oxidation intermediates [14]. Many reactions with wine nucleophile compounds will also occur, like those involving sulfur dioxide, ascorbic acid, amino acids, thiols or the A-ring of other flavonoid compounds [15,16,17]. Red wine is a very complex medium and many different chemical reactions may occur in a wine-dependent manner. Antioxidant species, such as sulfites, can also form adducts with condensed tannins on C4 position, preventing them from further undesirable reactions, such as nucleophilic addition [18]. Polyphenols can also act as hydroxyl radical scavengers. These radicals are formed through the Fenton reaction [19]. Some of these reactions involving the polyphenols catechol groups are summarized in Scheme 1.

Wine evolution (color, chemical composition, impact on polyphenolic composition), regarding different levels of oxygen, has already widely been described, notably by Petrozziello et al. [20]. Ferreira et al. [21] evaluated oxygen consumption for different wines. For both studies, it took several days or months to characterize and follow wine evolution. Accelerated ageing tests are very few and are mainly focused on heat test at 60 °C [22].

There is a need to develop new artificial wine ageing protocols to accelerate these oxidation processes in a shorter period at the laboratory compared to natural oxidations.

The objectives of this article are:

- To set up new and reproducible accelerated ageing tests for red wines.
- Identify oxidation markers involved in these tests by LC-MS.
- Apply the new tests to red wine samples from different vintages.

## 2. Materials and Methods

### 2.1. Materials

Hydrogen peroxide solution 30% (ACS reagent) and Laccase from Trametes versicolor (0.94 U/mg) were obtained from Sigma-Aldrich (Saint-Louis, MO, USA).

### 2.2. Model Wine Solution

The model wine solution was an ethanol water solution (12/88; *v/v*) with 0.033 M tartaric acid, adjusted to pH 3.6 with NaOH 1 M [23].

### 2.3. Wine Samples

Three red wines samples, 100% Syrah, 13.5% alcoholic strength, were obtained from the same producer (Domaine des Bouzons, Côtes du Rhône, France) and from three different vintages (2018, 2014, 2010). Wine production: 20 days vatting time in stainless steel vats; maturing of Syrah (40%) in oak barrels for 10 months. Two 750 mL bottles of each vintage were opened and slowly homogenized under nitrogen to avoid oxidation reactions. Aliquots of 50 mL tubes were then immediately frozen at −80 °C.

### 2.4. Wine Global Chemical Characterization

Global chemical analyses of the wine were performed by the Natoli laboratory (St Clément de Rivière, France) according to OIV procedures (www.oiv.int). Analyses included: alcoholic percentage (Fourier transformed infrared spectroscopy—FTIR); glucose and fructose (FTIR–Foss wine scan auto); total acidity (FTIR–Foss wine scan auto); volatile acidity; free, active and total sulfur dioxide (automated colorimetric method); pH; malic and lactic acid (FTIR–Foss wine scan auto); total polyphenols index (FTIR–Foss wine scan auto); CO_2_ (FTIR–Foss wine scan auto); Fe (Colorimetric method, reaction with disodium salt of (pyrildil-2)-3bis(phenyl-4-sulfonic 5–6 triazin-1,2,3,4) acid); absorbance read at 570 nm on a sequential analyzer (Olympus AU2700); Cu (colorimetric method 4-(3,5-Dibromo-2-Pyridilazo)-N-Ethyl-N-(3-Sulfopropyl)Aniline reaction; absorbance read at 570 nm on a sequential analyzer (Olympus AU2700). (supplementary data: Tables S1–S3).

### 2.5. Accelerated Ageing Tests

(a) Oxygen saturation: A previously −80 °C frozen wine sample (50 mL) was thawed and 35 mL was placed in a 500 mL closed flask. This wine sample was then saturated with air by vigorously shaking the flask for 10 s, after which the cap was opened (5 s) to let fresh air get into the flask. This saturation operation was repeated three times. Hermetic Pyrex 11 mL cultures tubes (VWR 734-4224, Radnor, PA, USA) containing Pst3 oxygen sensors (Presens—Precision Sensing GmbH, Regensburg, Germany) were filled with wine (11 mL) with a minimum headspace. It was previously shown that this procedure allows a headspace volume between 0 µL and 120 µL [21]. Further studies confirmed that less than 0.5 mg/L oxygen could pass through this closure, which is negligible for our accelerated test conditions. (b) Accelerated ageing heat test: Tubes containing oxygen saturated wine were heated (60 °C) and stirred (200 rpm) with a thermostatically-controlled stirrer (Hettich Benelux, Bäch, Switzerland). The dissolved oxygen level was monitored with an oxygen analyzer (Presens—Precision Sensing GmbH, Regensburg, Germany). The acquisition began when the temperature reached 60 °C in the tube. (c) Enzymatic and chemical oxidation: A tube containing wine saturated with oxygen was thermostatically-controlled (22 °C) and stirred (200 rpm). A 10 g/L laccase solution (50 µL) or a 30% vol. H_2_O_2_ solution (20 µL) was then added to the tube. The acquisition began 30 s after this addition. The oxidation test was stopped once O_2_ levels had values lower than 1 mg/L. All tests were performed in triplicate.

### 2.6. UV–Visible Measurements

The UV–vis spectra were determined with an Agilent Carry 60 spectrometer (Agilent technologies, Santa Clara, CA, USA) equipped with 1 mm cells. Model wine was used for the blank (see model wine section). Spectra were determined in a 400–800 nm UV range. 

### 2.7. UPLC-ESI-Tof Parameters

Analyses were performed using the same UPLC system and method, as described in Gil et al. [24] with slight modifications. The binary mobile phase consisted of Milli-Q water (solvent A) and acetonitrile (solvent B) both acidified with 0.1% formic acid. The separation was performed at a constant flow rate of 0.6 mL/min, using the following short gradient: 1% B for 1 min; 1–100% B in 0.5 min; 100% B for 2 min; 100–1% B in 1.5 min; equilibration at 1% B for 2 min. The injection volume was 10 µL. The mass spectrometer was operated in the positive ESI mode and data were collected for m/z from 100 to 2000 under the following conditions: capillary voltage, 2 kV; cone gas flow, 0 L/h; nitrogen desolvation gas flow, 1000 L/h; desolvation temperature, 350 °C; cone voltage, 60 V. All UPLC-ESI-Tof analyses were performed in triplicate.

### 2.8. Statistical Analysis

Statistical analyses were done by using XLSTAT 2020.1.1 software. For each oxidation protocol, sample color and intensity measurements were submitted to univariate analysis of variance (ANOVA) followed by a Tukey multiple comparison test (significance for *p* < 0.05).

## 3. Results and Discussion

### 3.1. Set Up and Application of Three Different Forced Oxidation Protocols for Syrah Wine Samples (2018, 2014, 2010)

**Figure 1:**
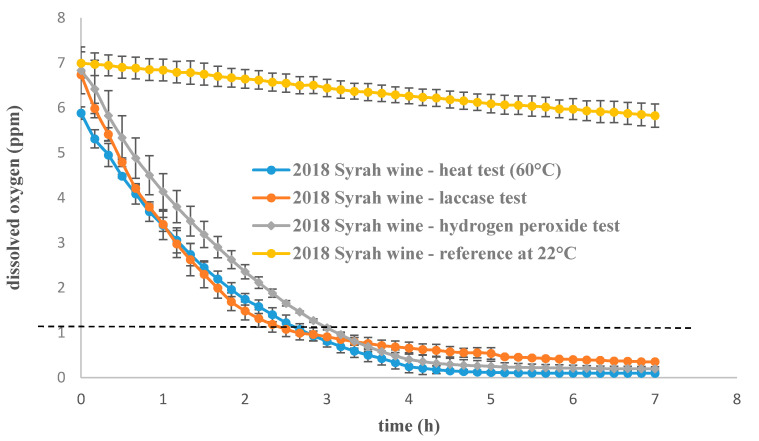
Rate of dissolved oxygen consumption in three different ageing tests used on a 2018 red wine sample (average, *n* = 3 or 4, and standard deviation for each point). Data are available in supplementary data: Tables S4–S7.

The dissolved oxygen concentration kinetics were monitored in a 2018 Syrah wine sample with three different accelerated oxidation protocols: heat test at 60 °C, laccase oxidation at 22 °C and hydrogen peroxide oxidation at 22 °C. Initial levels of dissolved oxygen were between 5.8 and 7 ppm for each experiment, as shown in Figure 1 and decreased rapidly under 1 ppm in less than 3 h until a final plateau was reached. The concentrations of dissolved oxygen at the plateau were: 0.1 ± *0.02* ppm for 60 °C experiment; 0.2 ± *0.04* ppm for hydrogen peroxide experiment; 0.2 ± *0.02* ppm for laccase experiment. No significant evolution was observed for the control sample over the experiment time; however, after 24 h the oxygen level in the control sample was measured at 4.36 ppm (average, *n* = 4). A complete oxygen consumption ([O_2_] < 1 ppm) was reached after five days. Even if the total oxygen consumption in a given wine sample is closely related to the wine composition [21], this consumption is significantly faster with an ageing test from five days to a few hours in the present experiment.

The curve profiles and the different threshold values indicated different behaviors depending on the oxidation test, indicating that different oxidation pathways may have occurred for each protocol.

Oxygen consumption rates were also investigated and revealed significant differences between oxidation tests. Oxygen is consumed approximately 1.4-times faster with the laccase test than the heat test at 60 °C and 1.2-times faster than with the hydrogen peroxide test, as shown in Table 1. The three different accelerated oxidation tests may target different wine constituents.

Temperature test: It has been established that wine oxidation is accelerated when the temperature increases [25]. This is the case for quinone formation which will lead to important changes in the wine evolution, as shown in Scheme 1. Another main oxidation reaction catalyzed by metals and accelerated with temperature is the anthocyanin degradation [26]. Anthocyanin are red grape pigments which accumulate in the skin during maturation [27,28]. As thermolabile compounds [29], they are the primary targets of chemical changes caused by an increase in temperature. The deglycosylation and cleavage of anthocyanins will lead to the release of the A and B rings of anthocyanins [30]. New compounds resulting from the degradation of anthocyanin are obtained, as benzoic acids. For example, malvidin 3-*O*-glucoside Baeyer–Villiger oxidation produces 2,4,6-trihydroxybenzaldehyde [31], syringic acid or anthocyanone A [32].

**Figure 2:**
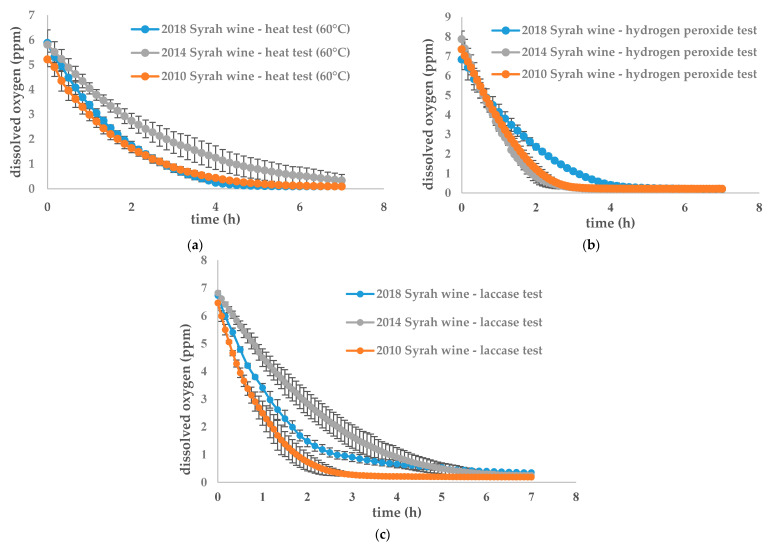
Evolution of dissolved oxygen concentration for three different wines (2018, 2014, 2010) oxidized with (**a**) heat test (60 °C); (**b**) hydrogen peroxide test; (**c**) laccase test. Data are available in supplementary data: Tables S4–S13.

The three oxidation protocols were then applied to three different wines from the same winery but from three different vintages—2018, 2014 and 2010—to identify different comportments between vintages regarding oxygen consumption, as shown in Figure 2.

For the 60 °C test, oxygen consumption rates, shown in Table 2a, indicate no significant difference between the three vintages. For the two other treatments, as shown in Table 2b,c, oxygen consumption was significantly different in the three different vintages. For the 2018 and 2010 vintages, the oxygen consumption rate was accelerated in the older wine. It can be hypothesized that oxidizable compound concentration decreases with years due to wine intrinsic oxidation mechanisms and that total oxidation is then faster. The 2014 wine follows this trend with the hydrogen peroxide test but not with the laccase test. The wine oxidation kinetic is not only correlated with age as some differences in vintages and wine composition may have an influence, as shown by Carascon et al. [33].

### 3.2. Colorimetric Analysis and Comparison of Three Untreated Wines from Different Vintages (2018, 2014 and 2010) and Three from 2018 Vintage Artificially Oxidized

**Figure 3:**
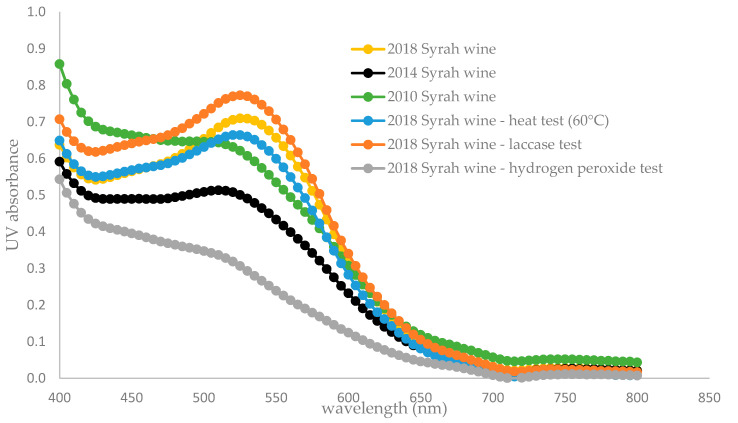
Absorbance measurements (400–800 nm) for six different wine samples: three untreated wines from different vintages (2018, 2014 and 2010) and three from 2018 vintage artificially oxidized. Data are available in supplementary data: Tables S14–S19.

As red wine oxidation induces color changes, UV measurements were done to compare natural and forced oxidation. Anthocyanins being the main compounds responsible for red color [27,28], their degradation due to oxidation could explain the differences in absorbance measurements, as shown in Table 3. At 520 nm, as shown in Figure 3, corresponding to the flavylium ring of anthocyanin, the 2018 Syrah wine sample had a profile significantly different from the other aged samples (2014 and 2010), which is coherent with a normal wine aging browning process. The 2018 wine at 60 °C followed the 2018 natural wine profile, as did the laccase one, with a maximal absorbance at 520 nm. It is possible that brown oxidized polyphenols were formed and could also increase the absorbance at 520 nm in this case, even if their maximal absorbance is around 420 nm [34]. The sample in the hydrogen peroxide test followed the natural oxidation profile of 2014 and 2010 natural samples, with a maximal absorbance around 420 nm corresponding to yellow and brown pigments. Forced oxidation with hydrogen peroxide seems to be closer to natural oxidation for absorbance measurements.

Other reactions resulting from the temperature increase can occur, such as the production of dioxane, dioxalane isomeres, furfural and 5-hydroxymethylfurfural, derived from carbohydrate dehydration and by cyclisation in Maillard type reactions. These reactions were investigated by Castro in Porto wines [22] under extreme oxidation procedures at 60 °C.

Hydrogen peroxide test: Hydrogen peroxide is notably involved in the Fenton reaction in wine, releasing a hydroxyl radical which is a strong oxidant, as shown in Scheme 1. It will then oxidize ethanol in acetaldehyde or accelerate quinone formation from polyphenols. This last reaction is more important in red wine than in other wine types. 

**Scheme 2:**
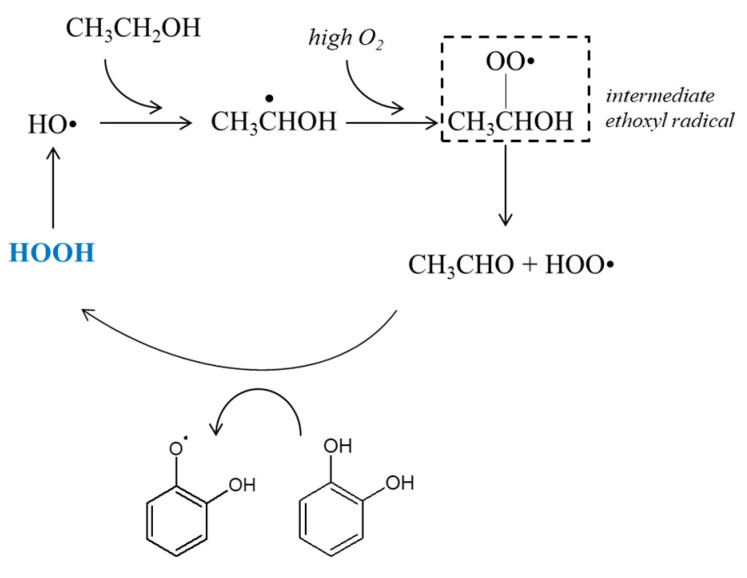
Proposed scheme depicting the pathway of non-enzymatic wine oxidation under high dissolved oxygen concentration.

This type of oxidation may be increased by a fast and high oxygen intake, favoring the formation of an intermediate ethoxyl radical, as shown in Scheme 2, instead of an immediate acetaldehyde formation from ethanol [19]. This radical leads to the formation of both acetaldehyde and HOO^•^ oxidizing polyphenols, oxygen consumption is consequently strongly increased by this mechanism.

Oxidation with hydrogen peroxide induces a radical color change. According to Figure 3, the absorbance spectrum for the sample with hydrogen peroxide is clearly different from the 2018 wine sample with a ratio of absorbance *Abs*(*420 nm*)/*Abs*(*520 nm*) and is significantly higher compared to other wine samples ratios, revealing a predominant yellow color of the oxidized sample and a strong decrease in the red pigments. It can be explained by the oxidation of malvidin 3-*O*-diglucoside in the presence of hydrogen peroxide under acidic conditions, which leads to the formation of ortho-benzoyloxyphenylacetic acid esters through Baeyer–Villiger oxidation type [35,36,37].

Laccase test: Laccase are polyphenoloxidaze enzyme types (PPO) which come from fungus and which especially oxidize 1,2 and 1,4-dihydroxybenzenes to ortho-benzoquinones, which are easily oxidisable species [8]. This enzymatic oxidation induces wine browning. As presented in Table 2, the absorbance at 420 nm and the ratio *Abs*(*420 nm*)/*Abs*(*520 nm*) for the wine enzymatically treated is significantly higher to the 2018 wine and close to the 2014 wine. The color is consequently browner (yellow pigments increase) and closer to the natural aging color. PPO action in wine is closely correlated to the hydroxycinnamates content and larger non-flavonoid polyphenols group in wine. In the presence of PPO, caffeoyl tartaric acid will be oxidized in benzoquinones [38], which act as electrophiles and as oxidants on substances with lower pH as polyphenols.

Laccases seem to be more selective concerning the oxidation targets as they lead to the formation of electrophile *ortho*-quinones on the flavonoids B-ring containing a catechol group, suffering then from nucleophilic attack by other polyphenols [39,40], particularly from the flavonoids A-ring [41]. The two other oxidation types have a larger range of action, not only attacking a specific site. Moreover, hydrogen peroxide releases a very strong oxidant HO•, which can impact a wide range of polyphenols, even less accessible ones, whereas a high temperature leads to numerous secondary reactions, such as anthocyanin degradation. The three different forced oxidations can generate other unknown reactions with the numerous antioxidant species present in wine (ascorbic acid, sulfites), which can also explain the different trends observed in Figure 3.

### 3.3. UPLC-ESI-QTof

**Figure 4:**
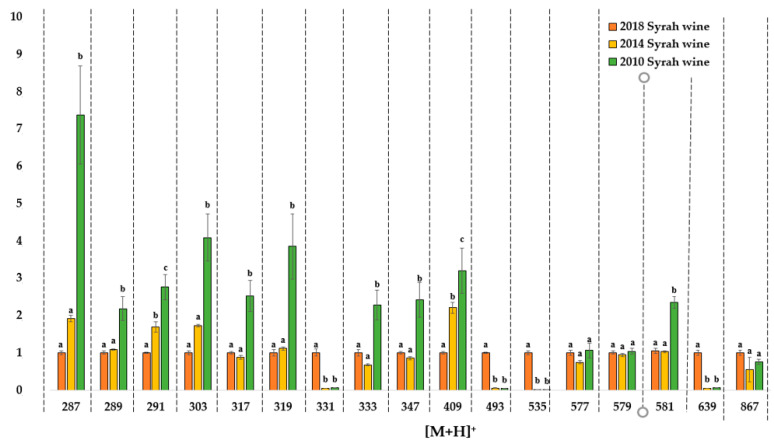
Natural oxidation samples. Ion intensity comparison of high-resolution MS spectra. Intensities for the three wine samples (2018, 2014 and 2010) are given for each [M+H]^+^ ion and normalized compared to the 2018 sample values. Figures are expressed as mean values (*n* = 3) with standard deviation. Different letters indicate a significant difference (Tukey test, *α* = 0.05) between vintages for a same [M+H]^+^ ion. Data are available in supplementary data: Tables S20–S22.

**Figure 5:**
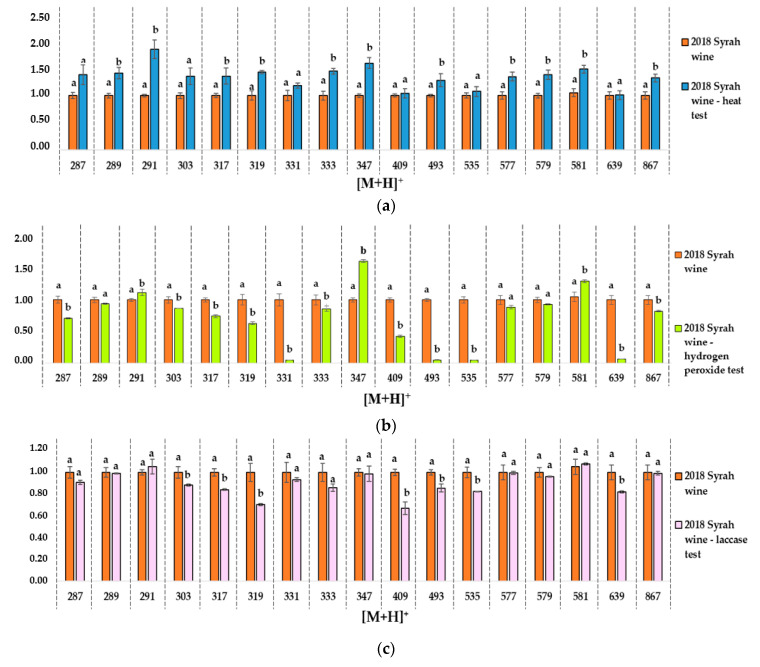
Ion intensity evolution in high resolution MS spectra. Intensity for the control wine aged 2018 and the ageing tests (**a**). Heat test −60 °C (**b**). Hydrogen peroxide test (**c**). Laccase tests. Results are given for each [M+H]^+^ ion compared to the 2018 sample. Intensities are expressed as mean values with standard deviation. Different letters indicate a significant difference (Tukey test, *α* = 0.05) between samples for the same [M+H]^+^ ion. Data are available in supplementary data: Tables S20, S23–S25.

High resolution UPLC-Q-Tof-MS was performed on three Syrah vintages—2018, 2014 and 2010—to observe natural wine oxidation. It was also performed on three 2018 forced oxidation samples (heat test, laccase test, hydrogen peroxide test) and compared to previous samples issued from natural evolution to detect similarities or differences between forced and natural ageing. Full scan positive mass spectra were acquired by a rapid metabolomics method [31]. A prefiltering of the ions with an intensity equal to or above 20% of the maximal intensity was applied to all samples to focus on the major ions. The intensities of these ions are presented in Figure 4 (natural oxidation samples) and Figure 5 (comparison between 2018 non oxidized and forced oxidation samples) The ion intensities are standardized compared to the 2018 sample.

Possible molecular attributions of the ions are presented in Table 4. The effect on some specific polyphenols (anthocyanin, flavanols and their derivatives) were observed on mass spectra in both naturally or artificially aged wines samples and differed depending on the oxidation protocol, as shown in Figure 5.

This semi-targeted approach allowed us to identify some specific ageing or vintage ion markers in the Syrah wines from different vintages. Ion intensities increased in mass spectra with the wine age for ions with [M+H]^+^ = 287, 289, 291, 303, 319, 333, 347, 409, 581 Da, which can be attributed to ageing markers, with significant differences between the three vintages for [M+H]^+^ ions detected at 291 and 409 Da. The 2014 wine can be too close to the 2018 one to observe significant differences for the following ions: 287, 289, 303, 319, 581 Da. However, the 2010 wine is old enough to observe very significant differences compared to the 2018 wine for some ions. For example, a 750% intensity increase was observed for the [M+H]^+^ ion at 287 Da and 250% for 581 Da between the 2018 and 2010 wines, which corresponds to an increase in the monomeric flavanol ions on the mass spectra.

Some ion intensities decreased with the age of the wines, such as 331, 493, 535, 639 [M+H]^+^. This was observed both for the 2014 and 2010 wine samples. It can be hypothesized that these ions correspond to malvidin derivatives, as shown in Table 4. Malvidin 3-*O*-glucoside is one of the main red pigments in wine and it is particularly sensitive to oxidation, as with most of the anthocyanins [2,5]. This intensity decrease was not observed for the 60 °C aging test, as shown in Figure 5a, and the laccase test, suggesting that other more sensitive molecules are impacted in these tests, as shown in Figure 5c.

Similarities can be observed between natural aging and the different artificial ageing tests.

Heat test: The same tendency for the ions at [M+H]^+^ = 289, 291, 303, 317, 319, 333, 347, 581 Da is observed between natural aging and oxidation protocol at 60 °C, as shown in Figure 5a. Ions intensities increased both with wine ageing and after the heat test.

Hydrogen peroxide test: An intensity increase for [M+H]^+^ = 581 Da is reported for this test, as shown in Figure 5b, as for the natural aging between 2018 and 2010 wines. An opposite tendency, correlated to natural aging, is noted for ions with [M+H]^+^ = 493, 535, 639 Da. This oxidation test had a stronger impact on anthocyanin ([M+H]^+^ = 331, 493, 535 and 639 Da) and a more moderate impact on low molecular weight flavonoids ([M+H]^+^ = 291, 347 and 581 Da). Forced oxidation with hydrogen peroxide seems to be closer to natural oxidation than the two other tests for the chosen markers.

Laccase test: The same decreasing tendency as for hydrogen peroxide test is reported for ions with [M+H]^+^ = 493, 535, 639 Da. It is the only test with no similarities with natural ageing for increasing intensities.

## 4. Conclusions

Three reproducible accelerated ageing tests based on three oxidation protocols were developed in this study and tested on three Syrah red wine samples. Each test revealed specific oxidation or ageing ion markers with significant differences between tests.

Intensities of their corresponding molecular ions were measured on MS spectra and revealed differences between wines with different vintages and between wines with different ageing tests, potentially revealing the presence of fragments from molecule degradation. Anthocyanins and polyphenols containing a galloyl or catechol group were among these potential oxidation markers both for natural ageing and accelerated ageing tests. MS spectra measured for natural ageing showed that the intensity for molecular ions corresponding to free anthocyanins decreased and the intensities increased for those corresponding to monomeric flavanols. The same result was observed for the hydrogen peroxide test on 2018 wine, which seems to be closer to natural oxidation than other tests for these oxidation markers.

Further research is needed to compare the slow and “natural” ageing kinetics of different red wines and the results of these ageing tests. The ultimate objective will be to determine which test is the most accurate to predict red wine ageing capacity.

## Figures and Tables

**Table 1:** Dissolved oxygen consumption rates in three different accelerated ageing tests. Rates are expressed as mean values ± standard error. Different letters indicate a significant difference (Tukey test, *α* = 0.05) between oxidation protocols.

Oxidation Protocol	Oxygen Consumption Rate (ppm·h^−1^)
Heat test −60 °C	2.40 ± 0.06 ^a^
Laccase test	3.33 ± 0.05 ^b^
Hydrogen peroxide test	2.86 ± 0.28 ^c^

**Table 2:** Dissolved oxygen consumption rates for three different oxidized wines: (**a**) at 60 °C; (**b**) with a hydrogen peroxide solution; (**c**) with a laccase solution. Means with different lowercase letters are significantly different (Tukey test, *α* = 0.05).

(**a**)	
**Wine Sample**	**Oxygen Consumption Rate (ppm·h^−1^)**
2018 Syrah wine—heat test (60 °C)	2.40 ± 0.06 ^a^
2014 Syrah wine—heat test (60 °C)	1.77 ± 0.45 ^a^
2010 Syrah wine—heat test (60 °C)	2.28 ± 0.28 ^a^
(**b**)	
2018 Syrah wine—hydrogen peroxide test	2.86 ± 0.28 ^a^
2014 Syrah wine—hydrogen peroxide test	4.41 ± 0.33 ^b^
2010 Syrah wine—hydrogen peroxide test	3.65 ± 0.08 ^c^
(**c**)	
2018 Syrah wine—laccase test	3.33 ± 0.06 ^a^
2014 Syrah wine—laccase test	2.45 ± 0.34 ^b^
2010 Syrah wine—laccase test	4.54 ± 0.66 ^c^

**Table 3:** Mean of three replicates ± standard error. Means in the same column with different lowercase letters are significantly different (Tukey test, *α* = 0.05).

Wine Sample	Abs 420	Abs 520	420/520
2018	0.545 ± 0.002 ^a^	0.705 ± 0.003 ^a^	0.773 ± 0.001 ^a^
2014	0.499 ± 0.003 ^b^	0.508 ± 0.004 ^b^	0.982 ± 0.001 ^b^
2010	0.702 ± 0.006 ^c^	0.631 ± 0.006 ^c^	1.111 ± 0.001 ^c^
2018—heat test (60 °C)	0.554 ± 0.002 ^a^	0.664 ± 0.003 ^d^	0.834 ± 0.002 ^a^
2018—hydrogen peroxide test	0.435 ± 0.006 ^e^	0.318 ± 0.007 ^f^	1.365 ± 0.013 ^d^
2018—laccase test	0.620 ± 0.02 ^d^	0.770 ± 0.025 ^e^	0.806 ± 0.002 ^a,b^

**Table 4:** Ion annotation in high-resolution MS spectra.

[M+H]^+^	Ion Annotation
287	*Cyanidin, Kaempferol*
289	*Fragment from polymeric proanthocyanidin*
291	*(+)-catechin, (-)-epicatechin, Fragment from polymeric proanthocyanidin*
303	*Quercetin, delphinidin, fragment from quercetin 3-glucoside, fragment from quercetin glucuronide*
317	*Petunidin, isorhamnetin*
319	*Fragment from myricetin 3-glucoside, fragment from myricetin glucuronide*
331	*Malvidin*
333	*unknown*
347	*Syringetin*
409	*Retro Diels Alder fragment*
493	*Malvidin 3-O-glucoside*
535	*Malvidin 3-O-acetyl glucoside*
577	*Oxidized dimeric proanthocyanidin*
579	*Dimeric proanthocyanidin, p-hydroxyphenylpyranopeodin 3-O-glucoside*
581	*Oxidized dimeric proanthocyanidin*
639	*Guaiacylpyranomalvidin 3-O-glucoside*
867	*Trimeric proanthocyanidins*

## Supplementary Materials

The following are available online at https://www.mdpi.com/2076-3921/9/8/663/s1, Table S1: analytical characterization of 2018 wine; Table S2: analytical characterization of 2014 wine; Table S3: analytical characterization of 2010 wine; Table S4: evolution of dissolved oxygen in 2018 red wine at 22 °C; Table S5: evolution of dissolved oxygen in 2018 red wine at 60 °C; Table S6: evolution of dissolved oxygen in 2018 red wine—laccase oxidation test; Table S7: evolution of dissolved oxygen in 2018 red wine—hydrogen peroxide oxidation test; Table S8: evolution of dissolved oxygen in 2014 red wine at 60 °C; Table S9: evolution of dissolved oxygen in 2014 red wine—laccase test; Table S10: evolution of dissolved oxygen in 2014 red wine—hydrogen peroxide oxidation test; Table S11: evolution of dissolved oxygen in 2010 red wine at 60 °C; Table S12: evolution of dissolved oxygen in 2010 red wine—laccase oxidation test; Table S13: evolution of dissolved oxygen in 2010 red wine—hydrogen peroxide oxidation test; Table S14: Absorbance measurements in UVvis (400–800 nm) for 2018 red wine; Table S15: Absorbance measurements in UVvis (400–800 nm) for 2014 red wine; Table S16: Absorbance measurements in UVvis (400–800 nm) for 2010 red wine; Table S17: Absorbance measurements in UVvis (400–800 nm) for 2018 red wine at 60 °C; Table S18: Absorbance measurements in UVvis (400–800 nm) for 2018 red wine—laccase oxidation test; Table S19: Absorbance measurements in UVvis (400–800 nm) for 2018 red wine—hydrogen peroxide oxidation test; Table S20: High resolution UPLC-MS for 2018 red wine; Table S21: High resolution UPLC-MS for 2014 red wine; Table S22: High resolution UPLC-MS for 2010 red wine; Table S23: High resolution UPLC-MS for 2018 wine at 60 °C; Table S24: High resolution UPLC-MS for 2018 red wine—laccase oxidation test; Table S25: High resolution UPLC-MS for 2018 wine—hydrogen peroxide oxidation test.
