# Supplementary material for: Red Wine Oxidation: Accelerated Ageing Tests, Possible Reaction Mechanisms and Application to Syrah Red Wines
Source: Antioxidants (Basel). 2020 Jul 24;9(8):663. doi: 10.3390/antiox9080663 (PMC7464692; doi:10.3390/antiox9080663)
Supplement: Supplementary file 1 [file antioxidants-09-00663-s001.pdf]

# SUPPLEMENTARY DATA

## TABLE OF CONTENTS


I. Original wines – analytical characterization ....... 3
1) 2018 wine ....... 3
2) 2014 wine ....... 3
3) 2010 wine ....... 4
II. Forced ageing protocols ....... 5
1) 2018 wine ....... 5
a. Reference at 22°C ....... 5
b. 60°C ....... 8
c. Laccase ....... 10
d. hydrogen peroxide ....... 13
2) 2014 wine ....... 15
a. 60°C ....... 15
b. Laccase ....... 18
c. hydrogen peroxide ....... 23
3) 2010 wine ....... 28
a. 60°C ....... 28
b. Laccase ....... 30
c. hydrogen peroxide ....... 35

III. Absorbance measurements ..... 40
1) 2018 wine ..... 40
2) 2014 wine ..... 43
3) 2010 wine ..... 46
4) 2018 wine at 60°C ..... 48
5) 2018 wine with laccase solution ..... 51
6) 2018 wine with hydrogen peroxide solution ..... 54
IV. High resolution UPLC-MS ..... 57
1) 2018 wine ..... 57
2) 2014 wine ..... 59
3) 2010 wine ..... 62
4) 2018 wine at 60°C ..... 63
5) 2018 wine with laccase solution ..... 65
6) 2018 wine with hydrogen peroxide solution ..... 67

# I. ORIGINAL WINES – ANALYTICAL CHARACTERIZATION

## 1) 2018 WINE

*Table S1 : analytical characterization of 2018 wine*

| | Result | Unit | Quantification limit | Detection limit | Uncertainty (95%) |
|---|---|---|---|---|---|
| **Alcoholic strength** | 14.25 | % vol. | | | 0.150 |
| **Glucose + Fructose** | ≤ 1.0 | g/L | 1.0 | 0.33 | N/V |
| **Total acidity** | 3.41 | g/L eq. $H_2SO_4$ | 2.0 | | 0.180 |
| **Volatil acidity** | 0.52 | g/L eq. $H_2SO_4$ | | | 0.050 |
| **Total $SO_2$** | 19 | mg/L | 10.0 | 3.3 | 15.0 |
| **Free $SO_2$** | 14 | mg/L | 5.0 | 1.667 | 6.0 |
| **pH** | 3.94 | | | | 0.080 |
| **Malic acid** | nd | g/L | 0.2 | 0.1 | N/V |
| **Lactic acid** | 2 | g/L | 0.2 | 0.1 | 0.30 |
| **Total polyphenol content** | 65 | | 5.0 | | 6.0 |
| **$CO_2$** | 260 | mg/L | 100.0 | | 130 |
| **Active $SO_2$** | 0.17 | mg/L | | | 0.000 |
| **Copper** | 0.3 | mg/L | 0.2 | | 0.20 |
| **Iron** | 2.7 | mg/L | 0.5 | | 0.50 |

## 2) 2014 WINE

*Table S2 : analytical characterization of 2014 wine*

| | Result | Unit | Quantification limit | Detection limit | Uncertainty (95%) |
|---|---|---|---|---|---|
| **Alcoholic strength** | 14.24 | % vol. | | | 0.150 |

| | | | | | |
|---|---|---|---|---|---|
| **Glucose + Fructose** | nd | g/L | 1.0 | 0.33 | N/V |
| **Total acidity** | 3.27 | g/L eq. $H_2SO_4$ | 2.0 | | 0.180 |
| **Volatil acidity** | 0.5 | g/L eq. $H_2SO_4$ | | | 0.050 |
| **Total $SO_2$** | ≤ 10.0 | mg/L | 10.0 | 3.3 | N/A |
| **Free $SO_2$** | ≤ 5.0 | mg/L | 5.0 | 1.667 | N/A |
| **pH** | 3.73 | | | | 0.080 |
| **Malic acid** | nd | g/L | 0.2 | 0.1 | N/V |
| **Lactic acid** | 1.5 | g/L | 0.2 | 0.1 | 0.30 |
| **Total polyphenol content** | 58 | | 5.0 | | 6.0 |
| **$CO_2$** | 510 | mg/L | 100.0 | | 130 |
| **Active $SO_2$** | 0.1 | mg/L | | | 0.000 |
| **Copper** | 0.3 | mg/L | 0.2 | | 0.20 |
| **Iron** | 2.7 | mg/L | 0.5 | | 0.50 |

## 3) 2010 WINE

*Table S3 : analytical characterization of 2010 wine*

| | Result | Unit | Quantification limit | Detection limit | Uncertainty (95%) |
|---|---|---|---|---|---|
| **Alcoholic strength** | 14.13 | % vol. | | | 0.150 |
| **Glucose + Fructose** | nd | g/L | 1.0 | 0.33 | N/V |
| **Total acidity** | 3.03 | g/L eq. $H_2SO_4$ | 2.0 | | 0.180 |
| **Volatil acidity** | 0.43 | g/L eq. $H_2SO_4$ | | | 0.050 |
| **Total $SO_2$** | nd | mg/L | 10.0 | 3.3 | N/V |
| **Free $SO_2$** | ≤ 5.0 | mg/L | 5.0 | 1.667 | N/V |
| **pH** | 3.83 | | | | 0.080 |
| **Malic acid** | nd | g/L | 0.2 | 0.1 | N/V |
| **Lactic acid** | 1.2 | g/L | 0.2 | 0.1 | 0.30 |
| **Total polyphenol content** | 72 | | 5.0 | | 6.0 |

| | | | | | |
|---|---|---|---|---|---|
| **$CO_2$** | 440 | mg/L | 100.0 | | 130 |
| **Active $SO_2$** | 0.06 | mg/L | | | 0.000 |
| **Copper** | 0.3 | mg/L | 0.2 | | 0.20 |
| **Iron** | 2.7 | mg/L | 0.5 | | 0.50 |

# II. FORCED AGEING PROTOCOLS

## 1) 2018 WINE

### a. Reference at 22°C

*Table S4 : evolution of dissolved oxygen in 2018 red wine at 22 °C*

| Time (h) | Temperature (°C) | Oxygen (ppm) n1 | Oxygen (ppm) n2 | Oxygen (ppm) n3 | Oxygen (ppm) n4 | Mean | Standard deviation | Variation coefficient (%) |
|---|---|---|---|---|---|---|---|---|
| 0 | 22 | 6,784 | 6,778 | 7,148 | 7,264 | 6,9935 | 0,24991399 | 3,57351806 |
| 0,167 | 22 | 6,747 | 6,76 | 7,122 | 7,247 | 6,969 | 0,25407217 | 3,64574786 |
| 0,334 | 22 | 6,747 | 6,76 | 7,057 | 7,214 | 6,9445 | 0,2297339 | 3,30814175 |
| 0,5 | 22 | 6,681 | 6,717 | 7,018 | 7,189 | 6,90125 | 0,24419169 | 3,53836905 |

| | | | | | | | | |
|---|---|---|---|---|---|---|---|---|
| 0,667 | 22 | 6,657 | 6,693 | 7,076 | 7,11 | 6,884 | 0,24217762 | 3,51797822 |
| 0,834 | 22 | 6,633 | 6,657 | 6,999 | 7,11 | 6,84975 | 0,24092789 | 3,5173239 |
| 1 | 22 | 6,615 | 6,644 | 7,044 | 7,058 | 6,84025 | 0,24370799 | 3,56285206 |
| 1,167 | 22 | 6,556 | 6,62 | 6,961 | 7,031 | 6,792 | 0,2387202 | 3,51472613 |
| 1,334 | 22 | 6,526 | 6,602 | 6,961 | 7,051 | 6,785 | 0,25968057 | 3,82727447 |
| 1,5 | 22 | 6,497 | 6,585 | 6,917 | 6,999 | 6,7495 | 0,24571189 | 3,64044582 |
| 1,667 | 22 | 6,462 | 6,561 | 6,867 | 6,917 | 6,70175 | 0,22429947 | 3,34687902 |
| 1,834 | 22 | 6,422 | 6,579 | 6,804 | 6,873 | 6,6695 | 0,20732342 | 3,10853016 |
| 2 | 22 | 6,404 | 6,544 | 6,78 | 6,848 | 6,644 | 0,2063266 | 3,10545758 |
| 2,167 | 22 | 6,37 | 6,543 | 6,743 | 6,823 | 6,61975 | 0,20393361 | 3,08068444 |
| 2,334 | 22 | 6,314 | 6,526 | 6,67 | 6,78 | 6,5725 | 0,20128504 | 3,06253387 |
| 2,5 | 22 | 6,269 | 6,529 | 6,694 | 6,707 | 6,54975 | 0,20394995 | 3,11385859 |
| 2,667 | 22 | 6,235 | 6,496 | 6,616 | 6,658 | 6,50125 | 0,19031093 | 2,92729752 |
| 2,834 | 22 | 6,252 | 6,479 | 6,616 | 6,67 | 6,50425 | 0,18639452 | 2,86573423 |
| 3 | 22 | 6,175 | 6,444 | 6,516 | 6,628 | 6,44075 | 0,19266443 | 2,99133536 |
| 3,167 | 22 | 6,142 | 6,393 | 6,504 | 6,581 | 6,405 | 0,19156374 | 2,99084685 |
| 3,334 | 22 | 6,12 | 6,393 | 6,423 | 6,533 | 6,36725 | 0,17547721 | 2,75593398 |
| 3,5 | 22 | 6,082 | 6,364 | 6,446 | 6,51 | 6,3505 | 0,18871054 | 2,97158549 |

| | | | | | | | | |
|---|---|---|---|---|---|---|---|---|
| 3,667 | 22 | 6,061 | 6,387 | 6,36 | 6,487 | 6,32375 | 0,18348728 | 2,90155816 |
| 3,834 | 22 | 6,034 | 6,341 | 6,371 | 6,417 | 6,29075 | 0,17399689 | 2,76591642 |
| 4 | 22 | 6,002 | 6,366 | 6,326 | 6,36 | 6,2635 | 0,17522081 | 2,79749043 |
| 4,167 | 22 | 5,965 | 6,33 | 6,286 | 6,36 | 6,23525 | 0,18271175 | 2,93030351 |
| 4,334 | 22 | 5,949 | 6,358 | 6,247 | 6,326 | 6,22 | 0,18659225 | 2,99987535 |
| 4,5 | 22 | 5,907 | 6,319 | 6,219 | 6,292 | 6,18425 | 0,18959848 | 3,06582818 |
| 4,667 | 22 | 5,881 | 6,316 | 6,191 | 6,236 | 6,156 | 0,19048185 | 3,094247 |
| 4,834 | 22 | 5,86 | 6,263 | 6,137 | 6,238 | 6,1245 | 0,18455442 | 3,01337935 |
| 5 | 22 | 5,794 | 6,26 | 6,158 | 6,153 | 6,09125 | 0,20420802 | 3,3524814 |
| 5,167 | 22 | 5,773 | 6,258 | 6,088 | 6,153 | 6,068 | 0,20876622 | 3,44044524 |
| 5,334 | 22 | 5,776 | 6,261 | 6,077 | 6,12 | 6,0585 | 0,2040727 | 3,36837005 |
| 5,5 | 22 | 5,728 | 6,256 | 6,05 | 6,12 | 6,0385 | 0,22397247 | 3,70907458 |
| 5,667 | 22 | 5,728 | 6,235 | 6,023 | 6,093 | 6,01975 | 0,21356244 | 3,5476962 |
| 5,834 | 22 | 5,708 | 6,185 | 5,986 | 6,023 | 5,9755 | 0,19816912 | 3,31636047 |
| 6 | 22 | 5,678 | 6,235 | 5,939 | 6,039 | 5,97275 | 0,23179067 | 3,88080322 |
| 6,167 | 22 | 5,648 | 6,158 | 5,897 | 6,037 | 5,935 | 0,21904794 | 3,69078248 |
| 6,334 | 22 | 5,594 | 6,174 | 5,886 | 6,007 | 5,91525 | 0,24455862 | 4,13437498 |
| 6,5 | 22 | 5,592 | 6,158 | 5,884 | 5,991 | 5,90625 | 0,23791367 | 4,02816804 |

| | | | | | | | | |
|---|---|---|---|---|---|---|---|---|
| 6,667 | 22 | 5,56 | 6,13 | 5,835 | 5,981 | 5,8765 | 0,24295198 | 4,13429736 |
| 6,834 | 22 | 5,531 | 6,112 | 5,789 | 5,978 | 5,8525 | 0,25198214 | 4,30554707 |
| 7 | 22 | 5,493 | 6,108 | 5,789 | 5,912 | 5,8255 | 0,2576671 | 4,42308983 |

**b. 60°C**

*Table S5 : evolution of dissolved oxygen in 2018 red wine at 60 °C*

| Time (h) | Temperature (°C) | Oxygen (ppm) n1 | Oxygen (ppm) n2 | Oxygen (ppm) n3 | Mean | Standard deviation | Variation coefficient (%) |
|---|---|---|---|---|---|---|---|
| 0 | 59.9 | 5.912 | 5.855 | 5.49 | 5.88 | 0.04 | 0.69 |
| 0.167 | 62.7 | 5.362 | 5.504 | 5.063 | 5.31 | 0.23 | 4.24 |
| 0.334 | 63.4 | 5.04 | 5.106 | 4.703 | 4.95 | 0.22 | 4.37 |
| 0.5 | 63.2 | 4.619 | 4.708 | 4.12 | 4.48 | 0.32 | 7.07 |
| 0.667 | 63.4 | 4.229 | 4.264 | 3.766 | 4.09 | 0.28 | 6.80 |
| 0.834 | 63.6 | 3.866 | 3.806 | 3.41 | 3.69 | 0.25 | 6.71 |
| 1 | 63.6 | 3.486 | 3.427 | 3.247 | 3.39 | 0.12 | 3.68 |
| 1.167 | 63.5 | 3.186 | 3.124 | 2.858 | 3.06 | 0.17 | 5.70 |
| 1.334 | 63.6 | 2.865 | 2.784 | 2.564 | 2.74 | 0.16 | 5.69 |
| 1.5 | 63.8 | 2.575 | 2.459 | 2.316 | 2.45 | 0.13 | 5.30 |
| 1.667 | 63.6 | 2.322 | 2.234 | 2.028 | 2.19 | 0.15 | 6.88 |

| | | | | | | | |
|---|---|---|---|---|---|---|---|
| 1.834 | 63.6 | 2.101 | 1.982 | 1.79 | 1.96 | 0.16 | 8.02 |
| 2 | 63.7 | 1.907 | 1.761 | 1.562 | 1.74 | 0.17 | 9.93 |
| 2.167 | 63.8 | 1.722 | 1.537 | 1.482 | 1.58 | 0.13 | 7.96 |
| 2.334 | 63.7 | 1.52 | 1.359 | 1.321 | 1.40 | 0.11 | 7.55 |
| 2.5 | 63.7 | 1.365 | 1.183 | 1.115 | 1.22 | 0.13 | 10.59 |
| 2.667 | 63.6 | 1.228 | 1.035 | 0.958 | 1.07 | 0.14 | 12.95 |
| 2.834 | 63.6 | 1.088 | 0.895 | 0.815 | 0.93 | 0.14 | 15.05 |
| 3 | 63.7 | 0.973 | 0.761 | 0.695 | 0.81 | 0.15 | 17.94 |
| 3.167 | 63.5 | 0.854 | 0.647 | 0.58 | 0.69 | 0.14 | 20.59 |
| 3.334 | 63.4 | 0.754 | 0.543 | 0.48 | 0.59 | 0.14 | 24.23 |
| 3.5 | 63.4 | 0.657 | 0.453 | 0.395 | 0.50 | 0.14 | 27.43 |
| 3.667 | 63.5 | 0.584 | 0.372 | 0.323 | 0.43 | 0.14 | 32.54 |
| 3.834 | 63.4 | 0.434 | 0.306 | 0.266 | 0.34 | 0.09 | 26.17 |
| 4 | 63.4 | 0.277 | 0.254 | 0.207 | 0.25 | 0.04 | 14.50 |
| 4.167 | 63.4 | 0.252 | 0.207 | 0.169 | 0.21 | 0.04 | 19.85 |
| 4.334 | 63.4 | 0.22 | 0.169 | 0.14 | 0.18 | 0.04 | 22.97 |
| 4.5 | 63.5 | 0.195 | 0.146 | 0.116 | 0.15 | 0.04 | 26.18 |
| 4.667 | 63.3 | 0.175 | 0.132 | 0.103 | 0.14 | 0.04 | 26.51 |
| 4.834 | 63.3 | 0.16 | 0.118 | 0.093 | 0.12 | 0.03 | 27.38 |

| | | | | | | | |
|---|---|---|---|---|---|---|---|
| 5 | 63.1 | 0.151 | 0.113 | 0.086 | 0.12 | 0.03 | 27.99 |
| 5.167 | 63.1 | 0.143 | 0.103 | 0.083 | 0.11 | 0.03 | 27.86 |
| 5.334 | 63.2 | 0.135 | 0.102 | 0.079 | 0.11 | 0.03 | 26.72 |
| 5.5 | 62.6 | 0.131 | 0.1 | 0.078 | 0.10 | 0.03 | 25.85 |
| 5.667 | 62.6 | 0.13 | 0.096 | 0.073 | 0.10 | 0.03 | 28.77 |
| 5.834 | 62.9 | 0.127 | 0.098 | 0.074 | 0.10 | 0.03 | 26.63 |
| 6 | 63.4 | 0.127 | 0.098 | 0.074 | 0.10 | 0.03 | 26.63 |
| 6.167 | 63.4 | 0.127 | 0.097 | 0.073 | 0.10 | 0.03 | 27.33 |
| 6.334 | 63.3 | 0.124 | 0.099 | 0.07 | 0.10 | 0.03 | 27.67 |
| 6.5 | 63.5 | 0.125 | 0.097 | 0.075 | 0.10 | 0.03 | 25.31 |
| 6.667 | 63.5 | 0.125 | 0.097 | 0.071 | 0.10 | 0.03 | 27.65 |
| 6.834 | 63.5 | 0.124 | 0.099 | 0.071 | 0.10 | 0.03 | 27.06 |
| 7 | 63.5 | 0.123 | 0.101 | 0.072 | 0.10 | 0.03 | 25.93 |

### c. Laccase

*Table S6 : evolution of dissolved oxygen in 2018 red wine - laccase oxidation test*

| Time (h) | Oxygen (ppm) n1 | Oxygen (ppm) n2 | Oxygen (ppm) n3 | Mean | Standard deviation | Variation coefficient (%) |
|---|---|---|---|---|---|---|

| | | | | | | |
|---|---|---|---|---|---|---|
| 0 | 6.733 | 6.668 | 6.809 | 6.74 | 0.07 | 1.05 |
| 0.167 | 5.995 | 6.093 | 5.869 | 5.99 | 0.11 | 1.88 |
| 0.333 | 5.291 | 5.576 | 5.365 | 5.41 | 0.15 | 2.73 |
| 0.5 | 4.704 | 4.894 | 4.777 | 4.79 | 0.10 | 2.00 |
| 0.667 | 4.178 | 4.156 | 4.296 | 4.21 | 0.08 | 1.79 |
| 0.833 | 3.739 | 3.783 | 3.87 | 3.80 | 0.07 | 1.76 |
| 1 | 3.353 | 3.584 | 3.293 | 3.41 | 0.15 | 4.51 |
| 1.167 | 2.957 | 3.285 | 2.684 | 2.98 | 0.30 | 10.11 |
| 1.333 | 2.664 | 2.96 | 2.251 | 2.63 | 0.36 | 13.57 |
| 1.5 | 2.367 | 2.556 | 1.974 | 2.30 | 0.30 | 12.91 |
| 1.667 | 2.132 | 2.115 | 1.732 | 1.99 | 0.23 | 11.35 |
| 1.833 | 1.906 | 1.636 | 1.527 | 1.69 | 0.20 | 11.55 |
| 2 | 1.715 | 1.394 | 1.345 | 1.48 | 0.20 | 13.54 |
| 2.167 | 1.54 | 1.213 | 1.195 | 1.32 | 0.19 | 14.76 |
| 2.333 | 1.384 | 1.108 | 1.067 | 1.19 | 0.17 | 14.53 |
| 2.5 | 1.264 | 1.017 | 0.953 | 1.08 | 0.16 | 15.23 |
| 2.667 | 1.157 | 0.955 | 0.863 | 0.99 | 0.15 | 15.17 |
| 2.833 | 1.057 | 1.032 | 0.792 | 0.96 | 0.15 | 15.24 |
| 3 | 0.978 | 1.023 | 0.727 | 0.91 | 0.16 | 17.54 |

| | | | | | | |
|---|---|---|---|---|---|---|
| 3.167 | 0.896 | 0.975 | 0.684 | 0.85 | 0.15 | 17.67 |
| 3.333 | 0.839 | 0.923 | 0.641 | 0.80 | 0.14 | 18.08 |
| 3.5 | 0.783 | 0.881 | 0.613 | 0.76 | 0.14 | 17.87 |
| 3.667 | 0.741 | 0.822 | 0.583 | 0.72 | 0.12 | 16.99 |
| 3.833 | 0.693 | 0.81 | 0.554 | 0.69 | 0.13 | 18.69 |
| 4 | 0.653 | 0.796 | 0.526 | 0.66 | 0.14 | 20.52 |
| 4.167 | 0.619 | 0.754 | 0.506 | 0.63 | 0.12 | 19.82 |
| 4.333 | 0.59 | 0.751 | 0.485 | 0.61 | 0.13 | 22.01 |
| 4.5 | 0.561 | 0.699 | 0.468 | 0.58 | 0.12 | 20.18 |
| 4.667 | 0.546 | 0.66 | 0.463 | 0.56 | 0.10 | 17.78 |
| 4.833 | 0.523 | 0.68 | 0.457 | 0.55 | 0.11 | 20.70 |
| 5 | 0.499 | 0.681 | 0.438 | 0.54 | 0.13 | 23.44 |
| 5.167 | 0.479 | 0.49 | 0.427 | 0.47 | 0.03 | 7.23 |
| 5.333 | 0.461 | 0.484 | 0.421 | 0.46 | 0.03 | 7.00 |
| 5.5 | 0.442 | 0.473 | 0.41 | 0.44 | 0.03 | 7.13 |
| 5.667 | 0.431 | 0.448 | 0.401 | 0.43 | 0.02 | 5.58 |
| 5.833 | 0.418 | 0.435 | 0.395 | 0.42 | 0.02 | 4.83 |
| 6 | 0.405 | 0.42 | 0.389 | 0.40 | 0.02 | 3.83 |
| 6.167 | 0.394 | 0.411 | 0.38 | 0.40 | 0.02 | 3.93 |

| | | | | | | |
|---|---|---|---|---|---|---|
| 6.333 | 0.382 | 0.394 | 0.38 | 0.39 | 0.01 | 1.97 |
| 6.5 | 0.372 | 0.376 | 0.37 | 0.37 | 0.00 | 0.82 |
| 6.667 | 0.36 | 0.375 | 0.365 | 0.37 | 0.01 | 2.08 |
| 6.833 | 0.348 | 0.363 | 0.353 | 0.35 | 0.01 | 2.15 |
| 7 | 0.344 | 0.362 | 0.355 | 0.35 | 0.01 | 2.57 |

### d. hydrogen peroxide

*Table S7 : evolution of dissolved oxygen in 2018 red wine – hydrogen peroxide oxidation test*

| Time (h) | Oxygen (ppm) n1 | Oxygen (ppm) n2 | Oxygen (ppm) n3 | mean | Standard deviation | Variation coefficient (%) |
|---|---|---|---|---|---|---|
| 0 | 6.231 | 7.099 | 7.167 | 6.83 | 0.52 | 7.64 |
| 0.167 | 5.69 | 6.696 | 6.873 | 6.42 | 0.64 | 9.94 |
| 0.334 | 5.186 | 6.179 | 6.113 | 5.83 | 0.56 | 9.53 |
| 0.5 | 4.794 | 5.74 | 5.474 | 5.34 | 0.49 | 9.14 |
| 0.667 | 4.393 | 5.277 | 4.97 | 4.88 | 0.45 | 9.20 |
| 0.834 | 4.045 | 4.907 | 4.549 | 4.50 | 0.43 | 9.62 |
| 1 | 3.724 | 4.522 | 4.163 | 4.14 | 0.40 | 9.66 |
| 1.167 | 3.428 | 4.139 | 3.838 | 3.80 | 0.36 | 9.39 |
| 1.334 | 3.142 | 3.8 | 3.506 | 3.48 | 0.33 | 9.46 |

| | | | | | | |
|---|---|---|---|---|---|---|
| 1.5 | 2.883 | 3.46 | 3.214 | 3.19 | 0.29 | 9.09 |
| 1.667 | 2.655 | 3.128 | 2.928 | 2.90 | 0.24 | 8.18 |
| 1.834 | 2.407 | 2.803 | 2.662 | 2.62 | 0.20 | 7.65 |
| 2 | 2.182 | 2.484 | 2.405 | 2.36 | 0.16 | 6.64 |
| 2.167 | 1.972 | 2.215 | 2.16 | 2.12 | 0.13 | 6.02 |
| 2.334 | 1.776 | 1.934 | 1.93 | 1.88 | 0.09 | 4.79 |
| 2.5 | 1.586 | 1.675 | 1.7 | 1.65 | 0.06 | 3.62 |
| 2.667 | 1.439 | 1.461 | 1.485 | 1.46 | 0.02 | 1.57 |
| 2.834 | 1.297 | 1.222 | 1.291 | 1.27 | 0.04 | 3.28 |
| 3 | 1.143 | 1.062 | 1.113 | 1.11 | 0.04 | 3.70 |
| 3.167 | 1.02 | 0.903 | 0.946 | 0.96 | 0.06 | 6.19 |
| 3.334 | 0.904 | 0.733 | 0.792 | 0.81 | 0.09 | 10.73 |
| 3.5 | 0.813 | 0.597 | 0.658 | 0.69 | 0.11 | 16.15 |
| 3.667 | 0.724 | 0.478 | 0.537 | 0.58 | 0.13 | 22.16 |
| 3.834 | 0.633 | 0.378 | 0.438 | 0.48 | 0.13 | 27.60 |
| 4 | 0.575 | 0.29 | 0.354 | 0.41 | 0.15 | 36.80 |
| 4.167 | 0.529 | 0.245 | 0.302 | 0.36 | 0.15 | 41.89 |
| 4.334 | 0.478 | 0.215 | 0.264 | 0.32 | 0.14 | 43.84 |
| 4.5 | 0.449 | 0.194 | 0.243 | 0.30 | 0.14 | 45.82 |

| | | | | | | |
|---|---|---|---|---|---|---|
| 4.667 | 0.419 | 0.178 | 0.233 | 0.28 | 0.13 | 45.65 |
| 4.834 | 0.395 | 0.169 | 0.228 | 0.26 | 0.12 | 44.40 |
| 5 | 0.379 | 0.158 | 0.222 | 0.25 | 0.11 | 44.95 |
| 5.167 | 0.337 | 0.154 | 0.222 | 0.24 | 0.09 | 38.92 |
| 5.334 | 0.323 | 0.154 | 0.218 | 0.23 | 0.09 | 36.83 |
| 5.5 | 0.308 | 0.148 | 0.217 | 0.22 | 0.08 | 35.77 |
| 5.667 | 0.295 | 0.145 | 0.22 | 0.22 | 0.08 | 34.09 |
| 5.834 | 0.286 | 0.144 | 0.219 | 0.22 | 0.07 | 32.84 |
| 6 | 0.276 | 0.141 | 0.216 | 0.21 | 0.07 | 32.06 |
| 6.167 | 0.264 | 0.143 | 0.216 | 0.21 | 0.06 | 29.34 |
| 6.334 | 0.252 | 0.141 | 0.216 | 0.20 | 0.06 | 27.90 |
| 6.5 | 0.244 | 0.142 | 0.213 | 0.20 | 0.05 | 26.19 |
| 6.667 | 0.24 | 0.144 | 0.218 | 0.20 | 0.05 | 25.06 |
| 6.834 | 0.235 | 0.144 | 0.22 | 0.20 | 0.05 | 24.44 |
| 7 | 0.232 | 0.141 | 0.216 | 0.20 | 0.05 | 24.75 |

## 2) 2014 WINE

### a. 60°C

*Table S8 : evolution of dissolved oxygen in 2014 red wine at 60 °C*

| Time (h) | Temperature (°C) | Oxygen (ppm) n1 | Oxygen (ppm) n2 | Oxygen (ppm) n3 | Oxygen (ppm) n4 | Mean | Standard deviation | Variation coefficient (%) |
|---|---|---|---|---|---|---|---|---|
| 0 | 59.7 | 5.444 | 5.89 | 6.629 | 5.288 | 5.813 | 0.601 | 10.339 |
| 0.167 | 60 | 5.323 | 5.441 | 6.125 | 5.195 | 5.521 | 0.415 | 7.517 |
| 0.334 | 60 | 5.15 | 5.17 | 5.77 | 4.852 | 5.236 | 0.385 | 7.351 |
| 0.5 | 60.1 | 4.788 | 4.927 | 5.353 | 4.501 | 4.892 | 0.355 | 7.250 |
| 0.667 | 60.1 | 4.627 | 4.634 | 5.008 | 4.231 | 4.625 | 0.317 | 6.860 |
| 0.834 | 60.1 | 4.473 | 4.374 | 4.585 | 3.956 | 4.347 | 0.275 | 6.316 |
| 1 | 60.1 | 4.159 | 4.165 | 4.169 | 3.711 | 4.051 | 0.227 | 5.596 |
| 1.167 | 60 | 3.969 | 3.856 | 3.852 | 3.478 | 3.789 | 0.214 | 5.652 |
| 1.334 | 60.3 | 3.722 | 3.706 | 3.591 | 3.246 | 3.566 | 0.221 | 6.206 |
| 1.5 | 60.1 | 3.565 | 3.506 | 3.281 | 3.022 | 3.344 | 0.247 | 7.382 |
| 1.667 | 60.1 | 3.416 | 3.359 | 2.967 | 2.848 | 3.148 | 0.282 | 8.969 |
| 1.834 | 60.1 | 3.205 | 3.169 | 2.722 | 2.624 | 2.930 | 0.300 | 10.232 |
| 2 | 60.3 | 3.013 | 2.992 | 2.503 | 2.456 | 2.741 | 0.303 | 11.043 |
| 2.167 | 60.3 | 2.858 | 2.898 | 2.304 | 2.269 | 2.582 | 0.342 | 13.252 |
| 2.334 | 60.3 | 2.724 | 2.731 | 2.112 | 2.105 | 2.418 | 0.357 | 14.781 |
| 2.5 | 60.3 | 2.612 | 2.635 | 1.927 | 1.939 | 2.278 | 0.399 | 17.505 |
| 2.667 | 60.3 | 2.423 | 2.558 | 1.767 | 1.792 | 2.135 | 0.414 | 19.405 |

| | | | | | | | | |
|---|---|---|---|---|---|---|---|---|
| 2.834 | 60.3 | 2.291 | 2.44 | 1.629 | 1.636 | 1.999 | 0.428 | 21.389 |
| 3 | 60.3 | 2.146 | 2.326 | 1.491 | 1.497 | 1.865 | 0.435 | 23.306 |
| 3.167 | 60.3 | 2.044 | 2.257 | 1.372 | 1.361 | 1.759 | 0.461 | 26.212 |
| 3.334 | 60.4 | 2.01 | 2.164 | 1.254 | 1.244 | 1.668 | 0.488 | 29.251 |
| 3.5 | 60.2 | 1.898 | 2.065 | 1.14 | 1.139 | 1.561 | 0.491 | 31.457 |
| 3.667 | 60.3 | 1.773 | 1.939 | 1.035 | 1.036 | 1.446 | 0.479 | 33.100 |
| 3.834 | 60.3 | 1.677 | 1.846 | 0.941 | 0.933 | 1.349 | 0.481 | 35.650 |
| 4 | 60.3 | 1.581 | 1.748 | 0.853 | 0.846 | 1.257 | 0.475 | 37.825 |
| 4.167 | 59.8 | 1.441 | 1.606 | 0.771 | 0.759 | 1.144 | 0.443 | 38.724 |
| 4.334 | 59.6 | 1.317 | 1.521 | 0.689 | 0.683 | 1.053 | 0.431 | 40.981 |
| 4.5 | 59.8 | 1.234 | 1.45 | 0.622 | 0.619 | 0.981 | 0.426 | 43.393 |
| 4.667 | 59.5 | 1.145 | 1.379 | 0.556 | 0.553 | 0.908 | 0.419 | 46.188 |
| 4.834 | 59.6 | 1.083 | 1.308 | 0.499 | 0.498 | 0.847 | 0.413 | 48.732 |
| 5 | 59.8 | 1.056 | 1.226 | 0.451 | 0.448 | 0.795 | 0.405 | 50.956 |
| 5.167 | 60.1 | 1.003 | 1.158 | 0.403 | 0.4 | 0.741 | 0.397 | 53.589 |
| 5.334 | 60.1 | 0.924 | 1.117 | 0.362 | 0.364 | 0.692 | 0.388 | 56.046 |
| 5.5 | 60.3 | 0.848 | 1.058 | 0.326 | 0.326 | 0.640 | 0.372 | 58.172 |
| 5.667 | 60.2 | 0.783 | 1.004 | 0.297 | 0.292 | 0.594 | 0.357 | 60.171 |
| 5.834 | 59.7 | 0.725 | 0.956 | 0.269 | 0.259 | 0.552 | 0.346 | 62.647 |

| | | | | | | | | |
|---|---|---|---|---|---|---|---|---|
| 6 | 59.8 | 0.681 | 0.953 | 0.246 | 0.231 | 0.528 | 0.352 | 66.703 |
| 6.167 | 59.8 | 0.653 | 0.91 | 0.222 | 0.209 | 0.499 | 0.343 | 68.857 |
| 6.334 | 60 | 0.618 | 0.857 | 0.203 | 0.189 | 0.467 | 0.328 | 70.178 |
| 6.5 | 60.1 | 0.567 | 0.806 | 0.188 | 0.169 | 0.433 | 0.309 | 71.490 |
| 6.667 | 60.1 | 0.521 | 0.732 | 0.172 | 0.158 | 0.396 | 0.280 | 70.773 |
| 6.834 | 59.9 | 0.484 | 0.685 | 0.158 | 0.142 | 0.367 | 0.264 | 71.891 |
| 7 | 59.5 | 0.438 | 0.639 | 0.148 | 0.134 | 0.340 | 0.244 | 71.757 |

## b. Laccase

*Table S9 : evolution of dissolved oxygen in 2014 red wine – laccase test*

| Time (h) | Oxygen (ppm) n1 | Oxygen (ppm) n2 | Oxygen (ppm) n3 | Oxygen (ppm) n4 | Oxygen (ppm) n5 | Mean | Standard deviation | Variation coefficient (%) |
|---|---|---|---|---|---|---|---|---|
| 0 | 7.044 | 6.859 | 6.897 | 7.099 | 7.217 | 7.023 | 0.147 | 2.095 |
| 0.083 | 6.798 | 6.639 | 6.681 | 6.92 | 7.066 | 6.821 | 0.175 | 2.572 |
| 0.167 | 6.545 | 6.359 | 6.468 | 6.753 | 6.952 | 6.615 | 0.237 | 3.582 |
| 0.25 | 6.36 | 6.126 | 6.28 | 6.537 | 6.777 | 6.416 | 0.250 | 3.902 |
| 0.333 | 6.115 | 5.949 | 6.061 | 6.341 | 6.662 | 6.226 | 0.283 | 4.540 |
| 0.417 | 5.913 | 5.738 | 5.897 | 6.169 | 6.496 | 6.043 | 0.297 | 4.912 |
| 0.5 | 5.728 | 5.521 | 5.713 | 5.986 | 6.246 | 5.839 | 0.281 | 4.818 |
| 0.583 | 5.435 | 5.342 | 5.521 | 5.789 | 6.125 | 5.642 | 0.317 | 5.621 |

| | | | | | | | | |
|---|---|---|---|---|---|---|---|---|
| 0.667 | 5.268 | 5.124 | 5.295 | 5.609 | 5.965 | 5.452 | 0.337 | 6.178 |
| 0.75 | 5.063 | 4.951 | 5.106 | 5.454 | 5.809 | 5.277 | 0.352 | 6.671 |
| 0.833 | 4.854 | 4.788 | 4.934 | 5.273 | 5.624 | 5.095 | 0.350 | 6.868 |
| 0.917 | 4.658 | 4.65 | 4.743 | 5.107 | 5.493 | 4.930 | 0.366 | 7.422 |
| 1 | 4.275 | 4.468 | 4.591 | 4.804 | 5.273 | 4.682 | 0.382 | 8.160 |
| 1.083 | 3.954 | 4.322 | 4.404 | 4.599 | 5.191 | 4.494 | 0.454 | 10.112 |
| 1.167 | 3.872 | 4.171 | 4.249 | 4.449 | 5.019 | 4.352 | 0.427 | 9.803 |
| 1.25 | 3.69 | 4.022 | 4.108 | 4.315 | 4.833 | 4.194 | 0.422 | 10.074 |
| 1.333 | 3.537 | 3.868 | 3.938 | 4.164 | 4.679 | 4.037 | 0.423 | 10.484 |
| 1.417 | 3.397 | 3.73 | 3.784 | 4.012 | 4.545 | 3.894 | 0.425 | 10.925 |
| 1.5 | 3.226 | 3.582 | 3.643 | 3.889 | 4.442 | 3.756 | 0.451 | 11.996 |
| 1.583 | 3.084 | 3.446 | 3.49 | 3.734 | 4.268 | 3.604 | 0.438 | 12.142 |
| 1.667 | 2.971 | 3.314 | 3.348 | 3.595 | 4.143 | 3.474 | 0.435 | 12.519 |
| 1.75 | 2.864 | 3.182 | 3.218 | 3.496 | 4.002 | 3.352 | 0.427 | 12.728 |
| 1.833 | 2.727 | 3.066 | 3.081 | 3.357 | 3.872 | 3.221 | 0.427 | 13.264 |
| 1.917 | 2.569 | 2.93 | 2.943 | 3.213 | 3.747 | 3.080 | 0.437 | 14.197 |
| 2 | 2.451 | 2.825 | 2.834 | 3.084 | 3.613 | 2.961 | 0.429 | 14.475 |
| 2.083 | 2.335 | 2.699 | 2.722 | 2.961 | 3.481 | 2.840 | 0.423 | 14.882 |
| 2.167 | 2.215 | 2.589 | 2.589 | 2.847 | 3.38 | 2.724 | 0.430 | 15.801 |

| | | | | | | | | |
|---|---|---|---|---|---|---|---|---|
| 2.25 | 2.097 | 2.49 | 2.468 | 2.717 | 3.273 | 2.609 | 0.433 | 16.583 |
| 2.333 | 1.994 | 2.391 | 2.368 | 2.6 | 3.161 | 2.503 | 0.428 | 17.095 |
| 2.417 | 1.897 | 2.284 | 2.259 | 2.494 | 3.063 | 2.399 | 0.429 | 17.862 |
| 2.5 | 1.791 | 2.166 | 2.174 | 2.385 | 2.948 | 2.293 | 0.424 | 18.501 |
| 2.583 | 1.666 | 2.091 | 2.072 | 2.29 | 2.832 | 2.190 | 0.424 | 19.377 |
| 2.667 | 1.6 | 1.985 | 1.974 | 2.186 | 2.741 | 2.097 | 0.418 | 19.909 |
| 2.75 | 1.5 | 1.902 | 1.883 | 2.089 | 2.662 | 2.007 | 0.424 | 21.125 |
| 2.833 | 1.416 | 1.82 | 1.785 | 1.978 | 2.544 | 1.909 | 0.411 | 21.513 |
| 2.917 | 1.258 | 1.748 | 1.7 | 1.891 | 2.449 | 1.809 | 0.429 | 23.702 |
| 3 | 1.152 | 1.651 | 1.616 | 1.811 | 2.362 | 1.718 | 0.435 | 25.343 |
| 3.083 | 1.128 | 1.587 | 1.535 | 1.735 | 2.277 | 1.652 | 0.415 | 25.135 |
| 3.167 | 1.107 | 1.512 | 1.462 | 1.645 | 2.151 | 1.575 | 0.378 | 24.013 |
| 3.25 | 1.049 | 1.435 | 1.382 | 1.553 | 2.024 | 1.489 | 0.353 | 23.710 |
| 3.333 | 0.985 | 1.373 | 1.313 | 1.482 | 1.963 | 1.423 | 0.354 | 24.880 |
| 3.417 | 0.923 | 1.302 | 1.248 | 1.408 | 1.904 | 1.357 | 0.355 | 26.186 |
| 3.5 | 0.865 | 1.247 | 1.179 | 1.337 | 1.886 | 1.303 | 0.371 | 28.499 |
| 3.583 | 0.812 | 1.19 | 1.119 | 1.269 | 1.77 | 1.232 | 0.347 | 28.169 |
| 3.667 | 0.757 | 1.124 | 1.06 | 1.203 | 1.673 | 1.163 | 0.331 | 28.465 |
| 3.75 | 0.711 | 1.074 | 1.001 | 1.141 | 1.614 | 1.108 | 0.327 | 29.494 |

| | | | | | | | | |
|---|---|---|---|---|---|---|---|---|
| 3.833 | 0.677 | 1.021 | 0.946 | 1.085 | 1.511 | 1.048 | 0.302 | 28.805 |
| 3.917 | 0.656 | 0.973 | 0.894 | 1.039 | 1.439 | 1.000 | 0.285 | 28.479 |
| 4 | 0.626 | 0.937 | 0.844 | 0.997 | 1.38 | 0.957 | 0.275 | 28.778 |
| 4.083 | 0.589 | 0.881 | 0.799 | 0.946 | 1.307 | 0.904 | 0.262 | 28.989 |
| 4.167 | 0.565 | 0.852 | 0.757 | 0.9 | 1.248 | 0.864 | 0.250 | 28.907 |
| 4.25 | 0.527 | 0.804 | 0.714 | 0.858 | 1.196 | 0.820 | 0.245 | 29.889 |
| 4.333 | 0.498 | 0.768 | 0.676 | 0.801 | 1.132 | 0.775 | 0.232 | 29.894 |
| 4.417 | 0.475 | 0.735 | 0.639 | 0.756 | 1.062 | 0.733 | 0.215 | 29.258 |
| 4.5 | 0.449 | 0.698 | 0.608 | 0.717 | 1.006 | 0.696 | 0.203 | 29.224 |
| 4.583 | 0.425 | 0.665 | 0.572 | 0.69 | 0.971 | 0.665 | 0.200 | 30.141 |
| 4.667 | 0.398 | 0.636 | 0.544 | 0.648 | 0.948 | 0.635 | 0.202 | 31.760 |
| 4.75 | 0.381 | 0.606 | 0.518 | 0.602 | 0.919 | 0.605 | 0.198 | 32.668 |
| 4.833 | 0.368 | 0.582 | 0.486 | 0.57 | 0.881 | 0.577 | 0.190 | 32.912 |
| 4.917 | 0.348 | 0.558 | 0.46 | 0.551 | 0.847 | 0.553 | 0.185 | 33.495 |
| 5 | 0.341 | 0.536 | 0.436 | 0.525 | 0.796 | 0.527 | 0.170 | 32.232 |
| 5.083 | 0.331 | 0.516 | 0.416 | 0.497 | 0.75 | 0.502 | 0.157 | 31.238 |
| 5.167 | 0.321 | 0.495 | 0.396 | 0.473 | 0.704 | 0.478 | 0.144 | 30.105 |
| 5.25 | 0.306 | 0.475 | 0.377 | 0.453 | 0.664 | 0.455 | 0.135 | 29.562 |
| 5.333 | 0.298 | 0.461 | 0.355 | 0.43 | 0.626 | 0.434 | 0.125 | 28.766 |

| | | | | | | | | |
|---|---|---|---|---|---|---|---|---|
| 5.417 | 0.284 | 0.442 | 0.343 | 0.418 | 0.599 | 0.417 | 0.119 | 28.592 |
| 5.5 | 0.273 | 0.43 | 0.327 | 0.406 | 0.568 | 0.401 | 0.112 | 28.066 |
| 5.583 | 0.266 | 0.412 | 0.311 | 0.389 | 0.54 | 0.384 | 0.105 | 27.464 |
| 5.667 | 0.266 | 0.4 | 0.302 | 0.37 | 0.516 | 0.371 | 0.097 | 26.165 |
| 5.75 | 0.256 | 0.377 | 0.287 | 0.358 | 0.498 | 0.355 | 0.094 | 26.472 |
| 5.833 | 0.248 | 0.374 | 0.277 | 0.343 | 0.477 | 0.344 | 0.090 | 26.135 |
| 5.917 | 0.243 | 0.358 | 0.268 | 0.33 | 0.441 | 0.328 | 0.078 | 23.860 |
| 6 | 0.239 | 0.347 | 0.258 | 0.318 | 0.425 | 0.317 | 0.074 | 23.434 |
| 6.083 | 0.236 | 0.343 | 0.247 | 0.307 | 0.421 | 0.311 | 0.076 | 24.324 |
| 6.167 | 0.232 | 0.334 | 0.24 | 0.295 | 0.455 | 0.311 | 0.091 | 29.100 |
| 6.25 | 0.226 | 0.33 | 0.231 | 0.283 | 0.413 | 0.297 | 0.078 | 26.190 |
| 6.333 | 0.225 | 0.326 | 0.225 | 0.271 | 0.352 | 0.280 | 0.058 | 20.710 |
| 6.417 | 0.223 | 0.311 | 0.22 | 0.263 | 0.342 | 0.272 | 0.054 | 19.819 |
| 6.5 | 0.223 | 0.304 | 0.216 | 0.258 | 0.356 | 0.271 | 0.059 | 21.651 |
| 6.583 | 0.219 | 0.3 | 0.215 | 0.254 | 0.362 | 0.270 | 0.062 | 22.866 |
| 6.667 | 0.218 | 0.297 | 0.209 | 0.247 | 0.359 | 0.266 | 0.062 | 23.420 |
| 6.75 | 0.219 | 0.29 | 0.204 | 0.243 | 0.359 | 0.263 | 0.063 | 23.868 |
| 6.833 | 0.212 | 0.286 | 0.199 | 0.24 | 0.364 | 0.260 | 0.067 | 25.721 |
| 6.917 | 0.215 | 0.278 | 0.196 | 0.241 | 0.357 | 0.257 | 0.064 | 24.710 |

| | | | | | | | | |
|---|---|---|---|---|---|---|---|---|
| 7 | 0.215 | 0.276 | 0.191 | 0.237 | 0.36 | 0.256 | 0.066 | 25.845 |

### c. hydrogen peroxide

*Table S10 : evolution of dissolved oxygen in 2014 red wine – hydrogen peroxide oxidation test*

| Time (h) | Oxygen (ppm) n1 | Oxygen (ppm) n2 | Oxygen (ppm) n3 | Mean | Standard deviation | Variation coefficient (%) |
|---|---|---|---|---|---|---|
| 0 | 8.053 | 7.39 | 8.166 | 7.870 | 0.419 | 5.327 |
| 0.084 | 7.627 | 6.861 | 7.7 | 7.396 | 0.465 | 6.284 |
| 0.167 | 7.202 | 6.47 | 7.243 | 6.972 | 0.435 | 6.239 |
| 0.25 | 6.806 | 5.971 | 6.899 | 6.559 | 0.511 | 7.792 |
| 0.334 | 6.378 | 5.459 | 6.487 | 6.108 | 0.565 | 9.245 |
| 0.417 | 5.955 | 5.084 | 6.072 | 5.704 | 0.540 | 9.465 |
| 0.5 | 5.58 | 4.787 | 5.678 | 5.348 | 0.489 | 9.135 |
| 0.584 | 5.137 | 4.563 | 5.263 | 4.988 | 0.373 | 7.481 |
| 0.667 | 4.795 | 4.325 | 4.861 | 4.660 | 0.292 | 6.272 |
| 0.75 | 4.391 | 4.121 | 4.513 | 4.342 | 0.201 | 4.620 |
| 0.834 | 4.069 | 3.852 | 4.163 | 4.028 | 0.160 | 3.960 |
| 0.917 | 3.729 | 3.434 | 3.799 | 3.654 | 0.194 | 5.301 |
| 1 | 3.381 | 3.139 | 3.448 | 3.323 | 0.163 | 4.892 |

| | | | | | | |
|---|---|---|---|---|---|---|
| 1.084 | 3.052 | 2.958 | 3.123 | 3.044 | 0.083 | 2.719 |
| 1.167 | 2.759 | 2.898 | 2.834 | 2.830 | 0.070 | 2.458 |
| 1.25 | 2.465 | 2.741 | 2.538 | 2.581 | 0.143 | 5.540 |
| 1.334 | 2.192 | 2.155 | 2.245 | 2.197 | 0.045 | 2.059 |
| 1.417 | 1.922 | 2.027 | 1.983 | 1.977 | 0.053 | 2.667 |
| 1.5 | 1.691 | 1.902 | 1.739 | 1.777 | 0.111 | 6.223 |
| 1.584 | 1.462 | 1.769 | 1.507 | 1.579 | 0.166 | 10.497 |
| 1.667 | 1.254 | 1.632 | 1.288 | 1.391 | 0.209 | 15.030 |
| 1.75 | 1.063 | 1.507 | 1.086 | 1.219 | 0.250 | 20.512 |
| 1.834 | 0.907 | 1.393 | 0.91 | 1.070 | 0.280 | 26.143 |
| 1.917 | 0.771 | 1.288 | 0.752 | 0.937 | 0.304 | 32.457 |
| 2 | 0.665 | 1.191 | 0.624 | 0.827 | 0.316 | 38.248 |
| 2.084 | 0.587 | 1.105 | 0.523 | 0.738 | 0.319 | 43.226 |
| 2.167 | 0.523 | 1.006 | 0.445 | 0.658 | 0.304 | 46.184 |
| 2.25 | 0.462 | 0.915 | 0.388 | 0.588 | 0.285 | 48.495 |
| 2.334 | 0.426 | 0.833 | 0.344 | 0.534 | 0.262 | 49.011 |
| 2.417 | 0.388 | 0.752 | 0.31 | 0.483 | 0.236 | 48.811 |
| 2.5 | 0.352 | 0.68 | 0.279 | 0.437 | 0.214 | 48.876 |
| 2.584 | 0.333 | 0.615 | 0.257 | 0.402 | 0.189 | 46.959 |

| | | | | | | |
|---|---|---|---|---|---|---|
| 2.667 | 0.316 | 0.554 | 0.238 | 0.369 | 0.165 | 44.570 |
| 2.75 | 0.299 | 0.495 | 0.229 | 0.341 | 0.138 | 40.435 |
| 2.834 | 0.284 | 0.446 | 0.216 | 0.315 | 0.118 | 37.471 |
| 2.917 | 0.279 | 0.395 | 0.21 | 0.295 | 0.093 | 31.727 |
| 3 | 0.27 | 0.35 | 0.2 | 0.273 | 0.075 | 27.459 |
| 3.084 | 0.268 | 0.314 | 0.195 | 0.259 | 0.060 | 23.169 |
| 3.167 | 0.258 | 0.289 | 0.192 | 0.246 | 0.050 | 20.111 |
| 3.25 | 0.252 | 0.266 | 0.19 | 0.236 | 0.040 | 17.139 |
| 3.334 | 0.245 | 0.249 | 0.19 | 0.228 | 0.033 | 14.460 |
| 3.417 | 0.242 | 0.24 | 0.186 | 0.223 | 0.032 | 14.268 |
| 3.5 | 0.241 | 0.231 | 0.183 | 0.218 | 0.031 | 14.201 |
| 3.584 | 0.237 | 0.232 | 0.181 | 0.217 | 0.031 | 14.303 |
| 3.667 | 0.243 | 0.226 | 0.181 | 0.217 | 0.032 | 14.786 |
| 3.75 | 0.237 | 0.223 | 0.178 | 0.213 | 0.031 | 14.496 |
| 3.834 | 0.239 | 0.22 | 0.178 | 0.212 | 0.031 | 14.701 |
| 3.917 | 0.231 | 0.219 | 0.176 | 0.209 | 0.029 | 13.859 |
| 4 | 0.235 | 0.221 | 0.176 | 0.211 | 0.031 | 14.633 |
| 4.084 | 0.232 | 0.22 | 0.175 | 0.209 | 0.030 | 14.378 |
| 4.167 | 0.232 | 0.22 | 0.178 | 0.210 | 0.028 | 13.502 |

| | | | | | | |
|---|---|---|---|---|---|---|
| 4.25 | 0.229 | 0.22 | 0.174 | 0.208 | 0.030 | 14.206 |
| 4.334 | 0.232 | 0.22 | 0.177 | 0.210 | 0.029 | 13.793 |
| 4.417 | 0.229 | 0.22 | 0.177 | 0.209 | 0.028 | 13.318 |
| 4.5 | 0.231 | 0.218 | 0.175 | 0.208 | 0.029 | 14.091 |
| 4.584 | 0.235 | 0.216 | 0.177 | 0.209 | 0.030 | 14.125 |
| 4.667 | 0.231 | 0.219 | 0.173 | 0.208 | 0.031 | 14.743 |
| 4.75 | 0.229 | 0.215 | 0.178 | 0.207 | 0.026 | 12.709 |
| 4.834 | 0.231 | 0.221 | 0.174 | 0.209 | 0.030 | 14.586 |
| 4.917 | 0.231 | 0.216 | 0.176 | 0.208 | 0.028 | 13.691 |
| 5 | 0.233 | 0.216 | 0.176 | 0.208 | 0.029 | 14.046 |
| 5.084 | 0.226 | 0.218 | 0.175 | 0.206 | 0.027 | 13.293 |
| 5.167 | 0.229 | 0.221 | 0.173 | 0.208 | 0.030 | 14.585 |
| 5.25 | 0.227 | 0.217 | 0.174 | 0.206 | 0.028 | 13.670 |
| 5.334 | 0.228 | 0.221 | 0.173 | 0.207 | 0.030 | 14.440 |
| 5.417 | 0.232 | 0.219 | 0.176 | 0.209 | 0.029 | 14.023 |
| 5.5 | 0.229 | 0.218 | 0.176 | 0.208 | 0.028 | 13.469 |
| 5.584 | 0.232 | 0.218 | 0.173 | 0.208 | 0.031 | 14.845 |
| 5.667 | 0.225 | 0.219 | 0.172 | 0.205 | 0.029 | 14.135 |
| 5.75 | 0.228 | 0.219 | 0.174 | 0.207 | 0.029 | 13.976 |

| | | | | | | |
|---|---|---|---|---|---|---|
| 5.834 | 0.228 | 0.215 | 0.176 | 0.206 | 0.027 | 13.116 |
| 5.917 | 0.226 | 0.222 | 0.17 | 0.206 | 0.031 | 15.166 |
| 6 | 0.224 | 0.218 | 0.174 | 0.205 | 0.027 | 13.296 |
| 6.084 | 0.225 | 0.22 | 0.173 | 0.206 | 0.029 | 13.926 |
| 6.167 | 0.223 | 0.219 | 0.174 | 0.205 | 0.027 | 13.251 |
| 6.25 | 0.23 | 0.219 | 0.174 | 0.208 | 0.030 | 14.288 |
| 6.334 | 0.23 | 0.22 | 0.172 | 0.207 | 0.031 | 14.954 |
| 6.417 | 0.23 | 0.22 | 0.173 | 0.208 | 0.030 | 14.656 |
| 6.5 | 0.229 | 0.221 | 0.173 | 0.208 | 0.030 | 14.585 |
| 6.584 | 0.224 | 0.221 | 0.175 | 0.207 | 0.027 | 13.290 |
| 6.667 | 0.225 | 0.223 | 0.173 | 0.207 | 0.029 | 14.233 |
| 6.75 | 0.224 | 0.221 | 0.173 | 0.206 | 0.029 | 13.892 |
| 6.834 | 0.228 | 0.224 | 0.172 | 0.208 | 0.031 | 15.020 |
| 6.917 | 0.226 | 0.224 | 0.173 | 0.208 | 0.030 | 14.465 |
| 7 | 0.226 | 0.224 | 0.177 | 0.209 | 0.028 | 13.268 |

## 3) 2010 WINE

### a. 60°C

*Table S11 : evolution of dissolved oxygen in 2010 red wine at 60 °C*

| Time (h) | Temperature (°C) | Oxygen (ppm) n1 | Oxygen (ppm) n2 | Oxygen (ppm) n3 | Mean | Standard deviation | Variation coefficient (%) |
|---|---|---|---|---|---|---|---|
| 0 | 60.2 | 4.917 | 5.24 | 5.505 | 5.221 | 0.294 | 5.641 |
| 0.167 | 60.7 | 4.515 | 4.943 | 5.28 | 4.913 | 0.383 | 7.804 |
| 0.333 | 60.9 | 3.97 | 4.301 | 4.803 | 4.358 | 0.419 | 9.624 |
| 0.5 | 60.9 | 3.643 | 3.843 | 4.407 | 3.964 | 0.396 | 9.994 |
| 0.667 | 61 | 3.417 | 3.435 | 4.018 | 3.623 | 0.342 | 9.436 |
| 0.833 | 61 | 3.172 | 3.106 | 3.636 | 3.305 | 0.289 | 8.740 |
| 1 | 60.9 | 2.912 | 2.796 | 3.256 | 2.988 | 0.239 | 8.006 |
| 1.167 | 60.9 | 2.654 | 2.514 | 2.978 | 2.715 | 0.238 | 8.765 |
| 1.333 | 60.9 | 2.389 | 2.243 | 2.684 | 2.439 | 0.225 | 9.212 |
| 1.5 | 60.9 | 2.159 | 2.005 | 2.453 | 2.206 | 0.228 | 10.320 |
| 1.667 | 61 | 1.948 | 1.862 | 2.229 | 2.013 | 0.192 | 9.535 |
| 1.833 | 60.9 | 1.775 | 1.649 | 2.028 | 1.817 | 0.193 | 10.621 |
| 2 | 60.9 | 1.606 | 1.48 | 1.835 | 1.640 | 0.180 | 10.972 |
| 2.167 | 61 | 1.466 | 1.331 | 1.668 | 1.488 | 0.170 | 11.396 |
| 2.333 | 60.9 | 1.326 | 1.213 | 1.526 | 1.355 | 0.159 | 11.698 |

| | | | | | | | |
|---|---|---|---|---|---|---|---|
| 2.5 | 60.9 | 1.2 | 1.043 | 1.377 | 1.207 | 0.167 | 13.848 |
| 2.667 | 61 | 1.072 | 0.973 | 1.25 | 1.098 | 0.140 | 12.780 |
| 2.833 | 61 | 0.95 | 0.869 | 1.121 | 0.980 | 0.129 | 13.128 |
| 3 | 60.9 | 0.855 | 0.745 | 1.008 | 0.869 | 0.132 | 15.194 |
| 3.167 | 60.9 | 0.76 | 0.646 | 0.904 | 0.770 | 0.129 | 16.791 |
| 3.333 | 60.9 | 0.683 | 0.565 | 0.816 | 0.688 | 0.126 | 18.252 |
| 3.5 | 60.9 | 0.608 | 0.515 | 0.743 | 0.622 | 0.115 | 18.431 |
| 3.667 | 60.7 | 0.546 | 0.441 | 0.661 | 0.549 | 0.110 | 20.031 |
| 3.833 | 60.9 | 0.481 | 0.394 | 0.578 | 0.484 | 0.092 | 19.005 |
| 4 | 60.9 | 0.429 | 0.366 | 0.519 | 0.438 | 0.077 | 17.556 |
| 4.167 | 60.7 | 0.38 | 0.299 | 0.468 | 0.382 | 0.085 | 22.107 |
| 4.333 | 60.9 | 0.337 | 0.255 | 0.413 | 0.335 | 0.079 | 23.588 |
| 4.5 | 60.9 | 0.301 | 0.225 | 0.367 | 0.298 | 0.071 | 23.872 |
| 4.667 | 60.7 | 0.268 | 0.198 | 0.315 | 0.260 | 0.059 | 22.615 |
| 4.833 | 60.7 | 0.236 | 0.178 | 0.286 | 0.233 | 0.054 | 23.164 |
| 5 | 60.7 | 0.211 | 0.16 | 0.259 | 0.210 | 0.050 | 23.575 |
| 5.167 | 60.7 | 0.187 | 0.147 | 0.23 | 0.188 | 0.042 | 22.079 |
| 5.333 | 60.7 | 0.169 | 0.137 | 0.204 | 0.170 | 0.034 | 19.712 |
| 5.5 | 60.7 | 0.152 | 0.123 | 0.182 | 0.152 | 0.030 | 19.366 |

| | | | | | | | |
|---|---|---|---|---|---|---|---|
| 5.667 | 60.7 | 0.14 | 0.119 | 0.166 | 0.142 | 0.024 | 16.619 |
| 5.833 | 60.6 | 0.128 | 0.109 | 0.154 | 0.130 | 0.023 | 17.333 |
| 6 | 60.6 | 0.118 | 0.108 | 0.145 | 0.124 | 0.019 | 15.477 |
| 6.167 | 60.6 | 0.109 | 0.103 | 0.137 | 0.116 | 0.018 | 15.600 |
| 6.333 | 60.6 | 0.104 | 0.101 | 0.127 | 0.111 | 0.014 | 12.853 |
| 6.5 | 60.6 | 0.098 | 0.098 | 0.123 | 0.106 | 0.014 | 13.574 |
| 6.667 | 60.7 | 0.092 | 0.094 | 0.116 | 0.101 | 0.013 | 13.228 |
| 6.833 | 60.6 | 0.09 | 0.093 | 0.115 | 0.099 | 0.014 | 13.742 |
| 7 | 60.6 | 0.085 | 0.088 | 0.111 | 0.095 | 0.014 | 15.026 |

## b. Laccase

*Table S12 : evolution of dissolved oxygen in 2010 red wine – laccase oxidation test*

| Time (h) | Oxygen (ppm) n1 | Oxygen (ppm) n2 | Oxygen (ppm) n3 | Mean | Standard deviation | Variation coefficient (%) |
|---|---|---|---|---|---|---|
| 0 | 7.222 | 7.521 | 7.202 | 7.315 | 0.179 | 2.443 |
| 0.083 | 7.019 | 7.151 | 6.769 | 6.980 | 0.194 | 2.780 |
| 0.167 | 6.481 | 6.647 | 6.27 | 6.466 | 0.189 | 2.922 |
| 0.25 | 6.056 | 5.965 | 5.965 | 5.995 | 0.053 | 0.876 |
| 0.333 | 5.492 | 5.416 | 5.599 | 5.502 | 0.092 | 1.671 |

| | | | | | | |
|---|---|---|---|---|---|---|
| 0.417 | 5.053 | 4.937 | 5.191 | 5.060 | 0.127 | 2.513 |
| 0.5 | 4.702 | 4.471 | 4.803 | 4.659 | 0.170 | 3.653 |
| 0.583 | 4.325 | 4.058 | 4.459 | 4.281 | 0.204 | 4.769 |
| 0.667 | 3.994 | 3.686 | 4.166 | 3.949 | 0.243 | 6.159 |
| 0.75 | 3.689 | 3.361 | 3.93 | 3.660 | 0.286 | 7.803 |
| 0.833 | 3.413 | 3.023 | 3.707 | 3.381 | 0.343 | 10.149 |
| 0.917 | 3.189 | 2.71 | 3.53 | 3.143 | 0.412 | 13.106 |
| 1 | 2.99 | 2.442 | 3.299 | 2.910 | 0.434 | 14.913 |
| 1.083 | 2.788 | 2.186 | 3.109 | 2.694 | 0.469 | 17.391 |
| 1.167 | 2.591 | 1.956 | 2.932 | 2.493 | 0.495 | 19.869 |
| 1.25 | 2.389 | 1.731 | 2.752 | 2.291 | 0.518 | 22.594 |
| 1.333 | 2.184 | 1.528 | 2.595 | 2.102 | 0.538 | 25.599 |
| 1.417 | 1.983 | 1.359 | 2.42 | 1.921 | 0.533 | 27.763 |
| 1.5 | 1.798 | 1.199 | 2.084 | 1.694 | 0.452 | 26.666 |
| 1.583 | 1.61 | 1.065 | 1.91 | 1.528 | 0.428 | 28.029 |
| 1.667 | 1.45 | 0.928 | 1.759 | 1.379 | 0.420 | 30.459 |
| 1.75 | 1.293 | 0.824 | 1.619 | 1.245 | 0.400 | 32.091 |
| 1.833 | 1.15 | 0.735 | 1.477 | 1.121 | 0.372 | 33.183 |
| 1.917 | 1.018 | 0.659 | 1.352 | 1.010 | 0.347 | 34.326 |

| | | | | | | |
|---|---|---|---|---|---|---|
| 2 | 0.906 | 0.587 | 1.232 | 0.908 | 0.323 | 35.505 |
| 2.083 | 0.804 | 0.527 | 1.119 | 0.817 | 0.296 | 36.270 |
| 2.167 | 0.715 | 0.475 | 1.024 | 0.738 | 0.275 | 37.293 |
| 2.25 | 0.638 | 0.439 | 0.939 | 0.672 | 0.252 | 37.460 |
| 2.333 | 0.576 | 0.4 | 0.843 | 0.606 | 0.223 | 36.787 |
| 2.417 | 0.518 | 0.366 | 0.749 | 0.544 | 0.193 | 35.429 |
| 2.5 | 0.474 | 0.342 | 0.662 | 0.493 | 0.161 | 32.642 |
| 2.583 | 0.434 | 0.318 | 0.587 | 0.446 | 0.135 | 30.229 |
| 2.667 | 0.4 | 0.296 | 0.524 | 0.407 | 0.114 | 28.069 |
| 2.75 | 0.374 | 0.278 | 0.475 | 0.376 | 0.099 | 26.223 |
| 2.833 | 0.346 | 0.263 | 0.425 | 0.345 | 0.081 | 23.503 |
| 2.917 | 0.329 | 0.252 | 0.388 | 0.323 | 0.068 | 21.114 |
| 3 | 0.314 | 0.243 | 0.358 | 0.305 | 0.058 | 19.025 |
| 3.083 | 0.298 | 0.233 | 0.335 | 0.289 | 0.052 | 17.888 |
| 3.167 | 0.29 | 0.227 | 0.312 | 0.276 | 0.044 | 15.965 |
| 3.25 | 0.277 | 0.22 | 0.299 | 0.265 | 0.041 | 15.366 |
| 3.333 | 0.267 | 0.213 | 0.282 | 0.254 | 0.036 | 14.288 |
| 3.417 | 0.26 | 0.21 | 0.276 | 0.249 | 0.034 | 13.845 |
| 3.5 | 0.25 | 0.202 | 0.27 | 0.241 | 0.035 | 14.521 |

| | | | | | | |
|---|---|---|---|---|---|---|
| 3.583 | 0.247 | 0.203 | 0.264 | 0.238 | 0.031 | 13.227 |
| 3.667 | 0.242 | 0.196 | 0.259 | 0.232 | 0.033 | 14.029 |
| 3.75 | 0.235 | 0.193 | 0.259 | 0.229 | 0.033 | 14.588 |
| 3.833 | 0.231 | 0.189 | 0.251 | 0.224 | 0.032 | 14.148 |
| 3.917 | 0.228 | 0.19 | 0.245 | 0.221 | 0.028 | 12.742 |
| 4 | 0.225 | 0.183 | 0.242 | 0.217 | 0.030 | 14.017 |
| 4.083 | 0.223 | 0.183 | 0.246 | 0.217 | 0.032 | 14.669 |
| 4.167 | 0.223 | 0.185 | 0.244 | 0.217 | 0.030 | 13.760 |
| 4.25 | 0.22 | 0.18 | 0.246 | 0.215 | 0.033 | 15.440 |
| 4.333 | 0.219 | 0.182 | 0.243 | 0.215 | 0.031 | 14.315 |
| 4.417 | 0.219 | 0.178 | 0.242 | 0.213 | 0.032 | 15.220 |
| 4.5 | 0.215 | 0.175 | 0.237 | 0.209 | 0.031 | 15.039 |
| 4.583 | 0.213 | 0.176 | 0.234 | 0.208 | 0.029 | 14.141 |
| 4.667 | 0.213 | 0.176 | 0.236 | 0.208 | 0.030 | 14.530 |
| 4.75 | 0.213 | 0.175 | 0.236 | 0.208 | 0.031 | 14.811 |
| 4.833 | 0.213 | 0.175 | 0.235 | 0.208 | 0.030 | 14.616 |
| 4.917 | 0.211 | 0.173 | 0.23 | 0.205 | 0.029 | 14.181 |
| 5 | 0.206 | 0.173 | 0.235 | 0.205 | 0.031 | 15.157 |
| 5.083 | 0.209 | 0.173 | 0.229 | 0.204 | 0.028 | 13.934 |

| | | | | | | |
|---|---|---|---|---|---|---|
| 5.167 | 0.209 | 0.169 | 0.233 | 0.204 | 0.032 | 15.875 |
| 5.25 | 0.209 | 0.169 | 0.231 | 0.203 | 0.031 | 15.484 |
| 5.333 | 0.207 | 0.173 | 0.23 | 0.203 | 0.029 | 14.103 |
| 5.417 | 0.206 | 0.169 | 0.223 | 0.199 | 0.028 | 13.851 |
| 5.5 | 0.205 | 0.167 | 0.221 | 0.198 | 0.028 | 14.032 |
| 5.583 | 0.205 | 0.167 | 0.221 | 0.198 | 0.028 | 14.032 |
| 5.667 | 0.202 | 0.168 | 0.219 | 0.196 | 0.026 | 13.226 |
| 5.75 | 0.2 | 0.17 | 0.219 | 0.196 | 0.025 | 12.583 |
| 5.833 | 0.202 | 0.17 | 0.218 | 0.197 | 0.024 | 12.427 |
| 5.917 | 0.202 | 0.164 | 0.218 | 0.195 | 0.028 | 14.248 |
| 6 | 0.203 | 0.168 | 0.214 | 0.195 | 0.024 | 12.318 |
| 6.083 | 0.203 | 0.168 | 0.218 | 0.196 | 0.026 | 13.069 |
| 6.167 | 0.203 | 0.17 | 0.218 | 0.197 | 0.025 | 12.465 |
| 6.25 | 0.201 | 0.168 | 0.213 | 0.194 | 0.023 | 12.012 |
| 6.333 | 0.201 | 0.164 | 0.215 | 0.193 | 0.026 | 13.629 |
| 6.417 | 0.2 | 0.166 | 0.212 | 0.193 | 0.024 | 12.384 |
| 6.5 | 0.201 | 0.165 | 0.21 | 0.192 | 0.024 | 12.402 |
| 6.583 | 0.202 | 0.168 | 0.212 | 0.194 | 0.023 | 11.889 |
| 6.667 | 0.202 | 0.165 | 0.21 | 0.192 | 0.024 | 12.482 |

| | | | | | | |
|---|---|---|---|---|---|---|
| 6.75 | 0.2 | 0.169 | 0.212 | 0.194 | 0.022 | 11.457 |
| 6.833 | 0.199 | 0.165 | 0.209 | 0.191 | 0.023 | 12.076 |
| 6.917 | 0.201 | 0.165 | 0.209 | 0.192 | 0.023 | 12.228 |
| 7 | 0.201 | 0.167 | 0.208 | 0.192 | 0.022 | 11.423 |

## c. hydrogen peroxide

*Table S13 : evolution of dissolved oxygen in 2010 red wine – hydrogen peroxide oxidation test*

| Time (h) | Oxygen (ppm) n1 | Oxygen (ppm) n2 | Oxygen (ppm) n3 | Oxygen (ppm) n4 | Mean | Standard deviation | Variation coefficient (%) |
|---|---|---|---|---|---|---|---|
| 0 | 7.177 | 7.332 | 7.438 | 7.454 | 7.350 | 0.128 | 1.735 |
| 0.083 | 6.894 | 6.939 | 7.052 | 7.195 | 7.020 | 0.134 | 1.913 |
| 0.167 | 6.582 | 6.685 | 6.744 | 6.874 | 6.721 | 0.122 | 1.813 |
| 0.25 | 6.259 | 6.333 | 6.522 | 6.593 | 6.427 | 0.157 | 2.438 |
| 0.333 | 5.913 | 6.057 | 6.209 | 6.253 | 6.108 | 0.155 | 2.534 |
| 0.417 | 5.585 | 5.724 | 5.81 | 6.019 | 5.785 | 0.182 | 3.142 |
| 0.5 | 5.268 | 5.426 | 5.454 | 5.718 | 5.467 | 0.187 | 3.413 |
| 0.583 | 4.95 | 5.123 | 5.106 | 5.416 | 5.149 | 0.194 | 3.776 |
| 0.667 | 4.669 | 4.811 | 4.82 | 5.066 | 4.842 | 0.165 | 3.405 |
| 0.75 | 4.391 | 4.505 | 4.54 | 4.779 | 4.554 | 0.163 | 3.581 |

| | | | | | | | |
|---|---|---|---|---|---|---|---|
| 0.833 | 4.082 | 4.216 | 4.288 | 4.478 | 4.266 | 0.165 | 3.870 |
| 0.917 | 3.777 | 3.923 | 4.051 | 4.245 | 3.999 | 0.199 | 4.965 |
| 1 | 3.503 | 3.657 | 3.828 | 3.983 | 3.743 | 0.208 | 5.558 |
| 1.083 | 3.23 | 3.375 | 3.587 | 3.711 | 3.476 | 0.215 | 6.177 |
| 1.167 | 2.962 | 3.112 | 3.358 | 3.457 | 3.222 | 0.226 | 7.018 |
| 1.25 | 2.719 | 2.848 | 3.136 | 3.203 | 2.977 | 0.231 | 7.748 |
| 1.333 | 2.496 | 2.597 | 2.94 | 3.003 | 2.759 | 0.250 | 9.066 |
| 1.417 | 2.241 | 2.361 | 2.729 | 2.771 | 2.526 | 0.264 | 10.468 |
| 1.5 | 2.003 | 2.135 | 2.535 | 2.593 | 2.317 | 0.292 | 12.596 |
| 1.583 | 1.798 | 1.928 | 2.33 | 2.372 | 2.107 | 0.287 | 13.631 |
| 1.667 | 1.595 | 1.716 | 2.151 | 2.149 | 1.903 | 0.290 | 15.228 |
| 1.75 | 1.404 | 1.511 | 1.974 | 1.965 | 1.714 | 0.299 | 17.440 |
| 1.833 | 1.232 | 1.324 | 1.815 | 1.771 | 1.536 | 0.300 | 19.553 |
| 1.917 | 1.074 | 1.152 | 1.644 | 1.603 | 1.368 | 0.297 | 21.701 |
| 2 | 0.938 | 1.012 | 1.495 | 1.438 | 1.221 | 0.286 | 23.454 |
| 2.083 | 0.836 | 0.871 | 1.347 | 1.273 | 1.082 | 0.266 | 24.559 |
| 2.167 | 0.732 | 0.742 | 1.205 | 1.135 | 0.954 | 0.252 | 26.393 |
| 2.25 | 0.653 | 0.641 | 1.069 | 0.987 | 0.838 | 0.223 | 26.574 |
| 2.333 | 0.575 | 0.555 | 0.941 | 0.858 | 0.732 | 0.196 | 26.800 |

| | | | | | | | |
|---|---|---|---|---|---|---|---|
| 2.417 | 0.518 | 0.487 | 0.821 | 0.739 | 0.641 | 0.164 | 25.601 |
| 2.5 | 0.471 | 0.433 | 0.713 | 0.625 | 0.561 | 0.131 | 23.417 |
| 2.583 | 0.436 | 0.389 | 0.612 | 0.538 | 0.494 | 0.100 | 20.337 |
| 2.667 | 0.411 | 0.347 | 0.525 | 0.461 | 0.436 | 0.075 | 17.312 |
| 2.75 | 0.382 | 0.317 | 0.452 | 0.397 | 0.387 | 0.056 | 14.348 |
| 2.833 | 0.364 | 0.299 | 0.389 | 0.35 | 0.351 | 0.038 | 10.823 |
| 2.917 | 0.343 | 0.275 | 0.343 | 0.307 | 0.317 | 0.033 | 10.329 |
| 3 | 0.331 | 0.258 | 0.305 | 0.288 | 0.296 | 0.031 | 10.363 |
| 3.083 | 0.315 | 0.243 | 0.285 | 0.273 | 0.279 | 0.030 | 10.681 |
| 3.167 | 0.309 | 0.234 | 0.266 | 0.261 | 0.268 | 0.031 | 11.601 |
| 3.25 | 0.297 | 0.227 | 0.252 | 0.252 | 0.257 | 0.029 | 11.344 |
| 3.333 | 0.293 | 0.22 | 0.236 | 0.243 | 0.248 | 0.032 | 12.704 |
| 3.417 | 0.289 | 0.212 | 0.233 | 0.242 | 0.244 | 0.033 | 13.331 |
| 3.5 | 0.281 | 0.212 | 0.227 | 0.235 | 0.239 | 0.030 | 12.455 |
| 3.583 | 0.273 | 0.206 | 0.221 | 0.235 | 0.234 | 0.029 | 12.287 |
| 3.667 | 0.267 | 0.203 | 0.217 | 0.232 | 0.230 | 0.028 | 11.975 |
| 3.75 | 0.265 | 0.2 | 0.213 | 0.232 | 0.228 | 0.028 | 12.415 |
| 3.833 | 0.264 | 0.199 | 0.211 | 0.229 | 0.226 | 0.028 | 12.547 |
| 3.917 | 0.258 | 0.198 | 0.21 | 0.23 | 0.224 | 0.026 | 11.710 |

| | | | | | | | |
|---|---|---|---|---|---|---|---|
| 4 | 0.258 | 0.193 | 0.21 | 0.228 | 0.222 | 0.028 | 12.504 |
| 4.083 | 0.252 | 0.193 | 0.21 | 0.227 | 0.221 | 0.025 | 11.416 |
| 4.167 | 0.252 | 0.193 | 0.205 | 0.223 | 0.218 | 0.026 | 11.756 |
| 4.25 | 0.25 | 0.197 | 0.205 | 0.228 | 0.220 | 0.024 | 10.877 |
| 4.333 | 0.25 | 0.19 | 0.205 | 0.221 | 0.217 | 0.026 | 11.857 |
| 4.417 | 0.252 | 0.191 | 0.202 | 0.224 | 0.217 | 0.027 | 12.393 |
| 4.5 | 0.25 | 0.19 | 0.203 | 0.224 | 0.217 | 0.026 | 12.098 |
| 4.583 | 0.248 | 0.19 | 0.203 | 0.224 | 0.216 | 0.025 | 11.737 |
| 4.667 | 0.246 | 0.187 | 0.203 | 0.22 | 0.214 | 0.025 | 11.791 |
| 4.75 | 0.246 | 0.189 | 0.202 | 0.223 | 0.215 | 0.025 | 11.612 |
| 4.833 | 0.246 | 0.187 | 0.202 | 0.227 | 0.216 | 0.026 | 12.151 |
| 4.917 | 0.244 | 0.187 | 0.202 | 0.222 | 0.214 | 0.025 | 11.576 |
| 5 | 0.244 | 0.185 | 0.201 | 0.221 | 0.213 | 0.026 | 11.992 |
| 5.083 | 0.246 | 0.185 | 0.201 | 0.215 | 0.212 | 0.026 | 12.238 |
| 5.167 | 0.245 | 0.183 | 0.202 | 0.221 | 0.213 | 0.027 | 12.462 |
| 5.25 | 0.239 | 0.187 | 0.202 | 0.224 | 0.213 | 0.023 | 10.822 |
| 5.333 | 0.245 | 0.184 | 0.201 | 0.218 | 0.212 | 0.026 | 12.270 |
| 5.417 | 0.242 | 0.184 | 0.2 | 0.221 | 0.212 | 0.025 | 11.912 |
| 5.5 | 0.242 | 0.187 | 0.202 | 0.221 | 0.213 | 0.024 | 11.183 |

| | | | | | | | |
|---|---|---|---|---|---|---|---|
| 5.583 | 0.242 | 0.185 | 0.2 | 0.221 | 0.212 | 0.025 | 11.726 |
| 5.667 | 0.239 | 0.18 | 0.202 | 0.221 | 0.211 | 0.025 | 12.034 |
| 5.75 | 0.24 | 0.184 | 0.199 | 0.222 | 0.211 | 0.025 | 11.707 |
| 5.833 | 0.234 | 0.184 | 0.2 | 0.221 | 0.210 | 0.022 | 10.563 |
| 5.917 | 0.24 | 0.183 | 0.201 | 0.22 | 0.211 | 0.025 | 11.628 |
| 6 | 0.24 | 0.187 | 0.205 | 0.221 | 0.213 | 0.023 | 10.600 |
| 6.083 | 0.235 | 0.184 | 0.198 | 0.217 | 0.209 | 0.022 | 10.671 |
| 6.167 | 0.237 | 0.182 | 0.201 | 0.224 | 0.211 | 0.024 | 11.564 |
| 6.25 | 0.237 | 0.187 | 0.2 | 0.215 | 0.210 | 0.021 | 10.236 |
| 6.333 | 0.239 | 0.181 | 0.199 | 0.22 | 0.210 | 0.025 | 12.007 |
| 6.417 | 0.232 | 0.182 | 0.203 | 0.216 | 0.208 | 0.021 | 10.151 |
| 6.5 | 0.236 | 0.186 | 0.198 | 0.219 | 0.210 | 0.022 | 10.578 |
| 6.583 | 0.236 | 0.184 | 0.201 | 0.215 | 0.209 | 0.022 | 10.534 |
| 6.667 | 0.235 | 0.187 | 0.199 | 0.221 | 0.211 | 0.022 | 10.244 |
| 6.75 | 0.236 | 0.185 | 0.197 | 0.221 | 0.210 | 0.023 | 10.978 |
| 6.833 | 0.236 | 0.182 | 0.2 | 0.222 | 0.210 | 0.024 | 11.349 |
| 6.917 | 0.237 | 0.186 | 0.2 | 0.22 | 0.211 | 0.022 | 10.620 |
| 7 | 0.232 | 0.183 | 0.2 | 0.22 | 0.209 | 0.022 | 10.373 |

# III. ABSORBANCE MEASUREMENTS

## 1) 2018 WINE

*Table S14 : Absorbance measurements in UVvis (400–800 nm) for 2018 red wine*

| Wavelength (nm) | Absorbance 1 | Absorbance 2 | Absorbance 3 | Mean | Standard deviation | Variation coefficient (%) |
|---|---|---|---|---|---|---|
| 800.0025 | 0.0241 | 0.0225 | 0.0171 | 0.0213 | 0.0037 | 17.2441 |
| 794.9929 | 0.0242 | 0.0229 | 0.0189 | 0.0220 | 0.0027 | 12.4837 |
| 790.0081 | 0.0249 | 0.0242 | 0.0190 | 0.0227 | 0.0032 | 14.2271 |
| 785.0137 | 0.0269 | 0.0256 | 0.0186 | 0.0237 | 0.0044 | 18.7486 |
| 780.0096 | 0.0272 | 0.0243 | 0.0193 | 0.0236 | 0.0040 | 16.7525 |
| 774.9960 | 0.0281 | 0.0273 | 0.0213 | 0.0256 | 0.0037 | 14.5163 |
| 770.0079 | 0.0281 | 0.0266 | 0.0222 | 0.0256 | 0.0031 | 12.1427 |
| 765.0104 | 0.0301 | 0.0274 | 0.0229 | 0.0268 | 0.0036 | 13.5921 |
| 760.0038 | 0.0293 | 0.0297 | 0.0243 | 0.0278 | 0.0030 | 10.7296 |
| 754.9877 | 0.0297 | 0.0285 | 0.0237 | 0.0273 | 0.0032 | 11.5681 |
| 749.9981 | 0.0307 | 0.0295 | 0.0239 | 0.0280 | 0.0037 | 13.0240 |
| 744.9995 | 0.0302 | 0.0283 | 0.0233 | 0.0273 | 0.0036 | 13.1469 |
| 739.9919 | 0.0309 | 0.0290 | 0.0249 | 0.0283 | 0.0030 | 10.7646 |
| 735.0110 | 0.0298 | 0.0280 | 0.0217 | 0.0265 | 0.0042 | 16.0202 |
| 729.9858 | 0.0289 | 0.0263 | 0.0207 | 0.0253 | 0.0042 | 16.4371 |
| 724.9877 | 0.0267 | 0.0254 | 0.0192 | 0.0238 | 0.0040 | 16.8347 |
| 720.0167 | 0.0252 | 0.0234 | 0.0172 | 0.0219 | 0.0042 | 18.9777 |
| 715.0015 | 0.0237 | 0.0222 | 0.0168 | 0.0209 | 0.0036 | 17.4212 |
| 710.0137 | 0.0250 | 0.0238 | 0.0186 | 0.0225 | 0.0034 | 15.3239 |
| 705.0178 | 0.0294 | 0.0275 | 0.0224 | 0.0264 | 0.0036 | 13.6591 |
| 700.0136 | 0.0349 | 0.0337 | 0.0279 | 0.0322 | 0.0038 | 11.6585 |

| | | | | | | |
|---|---|---|---|---|---|---|
| 695.0013 | 0.0413 | 0.0394 | 0.0337 | 0.0382 | 0.0039 | 10.3327 |
| 690.0170 | 0.0480 | 0.0461 | 0.0395 | 0.0445 | 0.0044 | 9.9782 |
| 684.9886 | 0.0534 | 0.0511 | 0.0455 | 0.0500 | 0.0040 | 8.0986 |
| 679.9885 | 0.0583 | 0.0566 | 0.0509 | 0.0553 | 0.0039 | 7.0064 |
| 675.0170 | 0.0644 | 0.0631 | 0.0565 | 0.0613 | 0.0042 | 6.9065 |
| 670.0014 | 0.0704 | 0.0688 | 0.0635 | 0.0675 | 0.0036 | 5.3237 |
| 665.0146 | 0.0756 | 0.0741 | 0.0680 | 0.0726 | 0.0040 | 5.5033 |
| 659.9838 | 0.0825 | 0.0811 | 0.0759 | 0.0798 | 0.0035 | 4.3925 |
| 654.9819 | 0.0914 | 0.0908 | 0.0848 | 0.0890 | 0.0036 | 4.0984 |
| 650.0092 | 0.1020 | 0.1018 | 0.0954 | 0.0997 | 0.0038 | 3.8117 |
| 644.9926 | 0.1156 | 0.1142 | 0.1080 | 0.1126 | 0.0040 | 3.5950 |
| 640.0055 | 0.1303 | 0.1301 | 0.1242 | 0.1282 | 0.0035 | 2.7201 |
| 635.0112 | 0.1484 | 0.1480 | 0.1422 | 0.1462 | 0.0035 | 2.3719 |
| 630.0099 | 0.1693 | 0.1682 | 0.1621 | 0.1666 | 0.0039 | 2.3279 |
| 625.0015 | 0.1899 | 0.1892 | 0.1831 | 0.1874 | 0.0037 | 1.9934 |
| 619.9862 | 0.2109 | 0.2099 | 0.2050 | 0.2086 | 0.0032 | 1.5141 |
| 615.0009 | 0.2357 | 0.2351 | 0.2291 | 0.2333 | 0.0037 | 1.5653 |
| 610.0089 | 0.2625 | 0.2616 | 0.2557 | 0.2599 | 0.0037 | 1.4281 |
| 605.0101 | 0.2905 | 0.2910 | 0.2856 | 0.2890 | 0.0030 | 1.0367 |
| 600.0048 | 0.3241 | 0.3235 | 0.3186 | 0.3221 | 0.0030 | 0.9271 |
| 594.9929 | 0.3578 | 0.3578 | 0.3514 | 0.3557 | 0.0037 | 1.0356 |
| 590.0115 | 0.3947 | 0.3949 | 0.3889 | 0.3929 | 0.0034 | 0.8655 |
| 584.9866 | 0.4354 | 0.4360 | 0.4281 | 0.4332 | 0.0044 | 1.0083 |
| 579.9926 | 0.4752 | 0.4771 | 0.4685 | 0.4736 | 0.0045 | 0.9534 |
| 574.9923 | 0.5128 | 0.5146 | 0.5075 | 0.5116 | 0.0037 | 0.7201 |
| 569.9858 | 0.5487 | 0.5506 | 0.5435 | 0.5476 | 0.0037 | 0.6710 |
| 565.0105 | 0.5796 | 0.5800 | 0.5733 | 0.5776 | 0.0037 | 0.6489 |
| 559.9918 | 0.6101 | 0.6097 | 0.6042 | 0.6080 | 0.0033 | 0.5426 |
| 555.0045 | 0.6360 | 0.6362 | 0.6282 | 0.6334 | 0.0045 | 0.7179 |
| 550.0113 | 0.6591 | 0.6592 | 0.6515 | 0.6566 | 0.0044 | 0.6730 |

| | | | | | | |
|---|---|---|---|---|---|---|
| 545.0122 | 0.6771 | 0.6792 | 0.6720 | 0.6761 | 0.0037 | 0.5475 |
| 540.0073 | 0.6930 | 0.6955 | 0.6878 | 0.6921 | 0.0039 | 0.5696 |
| 534.9968 | 0.7041 | 0.7066 | 0.6991 | 0.7033 | 0.0038 | 0.5440 |
| 530.0181 | 0.7099 | 0.7115 | 0.7048 | 0.7087 | 0.0035 | 0.4902 |
| 524.9963 | 0.7102 | 0.7121 | 0.7063 | 0.7095 | 0.0030 | 0.4186 |
| 520.0065 | 0.7060 | 0.7078 | 0.7022 | 0.7054 | 0.0028 | 0.4038 |
| 515.0114 | 0.6979 | 0.6993 | 0.6935 | 0.6969 | 0.0030 | 0.4325 |
| 510.0109 | 0.6851 | 0.6866 | 0.6819 | 0.6845 | 0.0024 | 0.3556 |
| 505.0050 | 0.6705 | 0.6716 | 0.6664 | 0.6695 | 0.0028 | 0.4128 |
| 499.9938 | 0.6546 | 0.6550 | 0.6511 | 0.6536 | 0.0022 | 0.3318 |
| 495.0154 | 0.6397 | 0.6411 | 0.6364 | 0.6390 | 0.0024 | 0.3755 |
| 489.9938 | 0.6255 | 0.6277 | 0.6224 | 0.6252 | 0.0026 | 0.4225 |
| 485.0052 | 0.6128 | 0.6140 | 0.6098 | 0.6122 | 0.0022 | 0.3585 |
| 480.0115 | 0.6019 | 0.6031 | 0.5992 | 0.6014 | 0.0020 | 0.3370 |
| 475.0129 | 0.5925 | 0.5940 | 0.5891 | 0.5919 | 0.0025 | 0.4208 |
| 470.0095 | 0.5854 | 0.5866 | 0.5821 | 0.5847 | 0.0024 | 0.4038 |
| 465.0010 | 0.5800 | 0.5805 | 0.5769 | 0.5791 | 0.0019 | 0.3338 |
| 459.9879 | 0.5752 | 0.5763 | 0.5723 | 0.5746 | 0.0021 | 0.3632 |
| 455.0083 | 0.5711 | 0.5716 | 0.5678 | 0.5702 | 0.0020 | 0.3579 |
| 449.9857 | 0.5659 | 0.5664 | 0.5623 | 0.5649 | 0.0022 | 0.3896 |
| 444.9969 | 0.5602 | 0.5613 | 0.5574 | 0.5596 | 0.0020 | 0.3560 |
| 440.0034 | 0.5551 | 0.5556 | 0.5521 | 0.5543 | 0.0019 | 0.3374 |
| 435.0056 | 0.5496 | 0.5501 | 0.5464 | 0.5487 | 0.0020 | 0.3673 |
| 430.0033 | 0.5447 | 0.5456 | 0.5418 | 0.5440 | 0.0020 | 0.3638 |
| 424.9967 | 0.5422 | 0.5433 | 0.5395 | 0.5417 | 0.0020 | 0.3608 |
| 419.9857 | 0.5456 | 0.5464 | 0.5431 | 0.5450 | 0.0017 | 0.3100 |
| 415.0091 | 0.5562 | 0.5577 | 0.5540 | 0.5559 | 0.0019 | 0.3342 |
| 409.9896 | 0.5754 | 0.5774 | 0.5729 | 0.5752 | 0.0023 | 0.3932 |
| 405.0047 | 0.6026 | 0.6040 | 0.6003 | 0.6023 | 0.0019 | 0.3142 |
| 400.0156 | 0.6368 | 0.6386 | 0.6360 | 0.6371 | 0.0013 | 0.2099 |

## 2) 2014 WINE

*Table S15 : Absorbance measurements in UVvis (400–800 nm) for 2014 red wine*

| Wavelength (nm) | Absorbance 1 | Absorbance 2 | Absorbance 3 | Mean | Standard deviation | Variation coefficient (%) |
|---|---|---|---|---|---|---|
| 800.003 | 0.024 | 0.020 | 0.018 | 0.020 | 0.003 | 15.114 |
| 794.993 | 0.024 | 0.021 | 0.018 | 0.021 | 0.003 | 14.483 |
| 790.008 | 0.025 | 0.021 | 0.019 | 0.022 | 0.003 | 14.073 |
| 785.014 | 0.026 | 0.021 | 0.022 | 0.023 | 0.002 | 10.797 |
| 780.010 | 0.026 | 0.022 | 0.022 | 0.023 | 0.002 | 7.953 |
| 774.996 | 0.027 | 0.022 | 0.022 | 0.024 | 0.003 | 11.967 |
| 770.008 | 0.029 | 0.024 | 0.023 | 0.026 | 0.003 | 11.623 |
| 765.010 | 0.028 | 0.024 | 0.023 | 0.025 | 0.003 | 10.114 |
| 760.004 | 0.028 | 0.025 | 0.023 | 0.025 | 0.003 | 10.108 |
| 754.988 | 0.030 | 0.026 | 0.024 | 0.026 | 0.003 | 11.273 |
| 749.998 | 0.029 | 0.024 | 0.024 | 0.026 | 0.003 | 11.101 |
| 744.999 | 0.028 | 0.024 | 0.023 | 0.025 | 0.002 | 9.122 |
| 739.992 | 0.028 | 0.024 | 0.022 | 0.025 | 0.003 | 10.309 |
| 735.011 | 0.027 | 0.024 | 0.022 | 0.024 | 0.002 | 10.047 |
| 729.986 | 0.026 | 0.021 | 0.021 | 0.023 | 0.003 | 12.051 |
| 724.988 | 0.023 | 0.019 | 0.018 | 0.020 | 0.003 | 13.639 |
| 720.017 | 0.021 | 0.018 | 0.016 | 0.018 | 0.003 | 14.194 |
| 715.002 | 0.021 | 0.017 | 0.015 | 0.018 | 0.003 | 16.770 |
| 710.014 | 0.022 | 0.018 | 0.017 | 0.019 | 0.003 | 14.009 |
| 705.018 | 0.026 | 0.022 | 0.020 | 0.023 | 0.003 | 13.155 |
| 700.014 | 0.031 | 0.027 | 0.025 | 0.028 | 0.003 | 10.097 |
| 695.001 | 0.036 | 0.032 | 0.031 | 0.033 | 0.003 | 8.028 |

| | | | | | | |
|---|---|---|---|---|---|---|
| 690.017 | 0.042 | 0.038 | 0.037 | 0.039 | 0.003 | 7.264 |
| 684.989 | 0.047 | 0.043 | 0.042 | 0.044 | 0.003 | 6.016 |
| 679.989 | 0.053 | 0.048 | 0.047 | 0.049 | 0.003 | 6.105 |
| 675.017 | 0.057 | 0.053 | 0.051 | 0.054 | 0.003 | 5.169 |
| 670.001 | 0.062 | 0.058 | 0.057 | 0.059 | 0.003 | 4.865 |
| 665.015 | 0.066 | 0.062 | 0.061 | 0.063 | 0.003 | 4.530 |
| 659.984 | 0.071 | 0.067 | 0.065 | 0.068 | 0.003 | 4.086 |
| 654.982 | 0.079 | 0.074 | 0.071 | 0.075 | 0.004 | 5.331 |
| 650.009 | 0.085 | 0.081 | 0.079 | 0.081 | 0.003 | 4.019 |
| 644.993 | 0.093 | 0.089 | 0.087 | 0.090 | 0.003 | 3.374 |
| 640.005 | 0.104 | 0.100 | 0.098 | 0.101 | 0.003 | 3.329 |
| 635.011 | 0.116 | 0.111 | 0.110 | 0.112 | 0.003 | 2.819 |
| 630.010 | 0.129 | 0.125 | 0.124 | 0.126 | 0.003 | 2.344 |
| 625.001 | 0.144 | 0.139 | 0.137 | 0.140 | 0.003 | 2.460 |
| 619.986 | 0.161 | 0.155 | 0.153 | 0.156 | 0.004 | 2.645 |
| 615.001 | 0.176 | 0.172 | 0.170 | 0.172 | 0.003 | 1.821 |
| 610.009 | 0.194 | 0.190 | 0.188 | 0.191 | 0.003 | 1.764 |
| 605.010 | 0.214 | 0.210 | 0.208 | 0.211 | 0.003 | 1.530 |
| 600.005 | 0.236 | 0.231 | 0.229 | 0.232 | 0.003 | 1.463 |
| 594.993 | 0.256 | 0.251 | 0.249 | 0.252 | 0.004 | 1.479 |
| 590.011 | 0.279 | 0.275 | 0.272 | 0.275 | 0.004 | 1.313 |
| 584.987 | 0.302 | 0.297 | 0.295 | 0.298 | 0.004 | 1.257 |
| 579.993 | 0.325 | 0.321 | 0.317 | 0.321 | 0.004 | 1.216 |
| 574.992 | 0.347 | 0.341 | 0.338 | 0.342 | 0.004 | 1.310 |
| 569.986 | 0.367 | 0.361 | 0.359 | 0.362 | 0.004 | 1.165 |
| 565.010 | 0.385 | 0.379 | 0.377 | 0.381 | 0.004 | 1.077 |
| 559.992 | 0.403 | 0.398 | 0.395 | 0.399 | 0.004 | 1.035 |
| 555.005 | 0.420 | 0.416 | 0.413 | 0.416 | 0.004 | 0.865 |
| 550.011 | 0.438 | 0.432 | 0.430 | 0.433 | 0.005 | 1.067 |
| 545.012 | 0.455 | 0.449 | 0.446 | 0.450 | 0.005 | 1.029 |

| | | | | | | |
|---|---|---|---|---|---|---|
| 540.007 | 0.469 | 0.463 | 0.461 | 0.464 | 0.004 | 0.841 |
| 534.997 | 0.483 | 0.477 | 0.475 | 0.478 | 0.004 | 0.880 |
| 530.018 | 0.495 | 0.489 | 0.487 | 0.491 | 0.004 | 0.764 |
| 524.996 | 0.504 | 0.499 | 0.498 | 0.500 | 0.003 | 0.689 |
| 520.007 | 0.512 | 0.507 | 0.505 | 0.508 | 0.004 | 0.780 |
| 515.011 | 0.515 | 0.511 | 0.509 | 0.512 | 0.003 | 0.614 |
| 510.011 | 0.517 | 0.512 | 0.510 | 0.513 | 0.003 | 0.661 |
| 505.005 | 0.516 | 0.510 | 0.509 | 0.512 | 0.003 | 0.650 |
| 499.994 | 0.512 | 0.507 | 0.506 | 0.508 | 0.003 | 0.545 |
| 495.015 | 0.509 | 0.504 | 0.503 | 0.505 | 0.003 | 0.663 |
| 489.994 | 0.505 | 0.500 | 0.499 | 0.501 | 0.003 | 0.627 |
| 485.005 | 0.501 | 0.496 | 0.495 | 0.497 | 0.003 | 0.630 |
| 480.012 | 0.498 | 0.493 | 0.492 | 0.494 | 0.003 | 0.652 |
| 475.013 | 0.494 | 0.490 | 0.488 | 0.491 | 0.003 | 0.643 |
| 470.009 | 0.492 | 0.488 | 0.487 | 0.489 | 0.003 | 0.582 |
| 465.001 | 0.493 | 0.488 | 0.487 | 0.489 | 0.003 | 0.621 |
| 459.988 | 0.493 | 0.488 | 0.487 | 0.489 | 0.003 | 0.609 |
| 455.008 | 0.493 | 0.489 | 0.487 | 0.490 | 0.003 | 0.609 |
| 449.986 | 0.493 | 0.488 | 0.487 | 0.489 | 0.003 | 0.592 |
| 444.997 | 0.493 | 0.488 | 0.487 | 0.489 | 0.003 | 0.662 |
| 440.003 | 0.493 | 0.488 | 0.487 | 0.489 | 0.003 | 0.636 |
| 435.006 | 0.493 | 0.488 | 0.487 | 0.489 | 0.003 | 0.659 |
| 430.003 | 0.493 | 0.488 | 0.487 | 0.490 | 0.003 | 0.636 |
| 424.997 | 0.496 | 0.491 | 0.489 | 0.492 | 0.004 | 0.715 |
| 419.986 | 0.502 | 0.497 | 0.496 | 0.499 | 0.003 | 0.668 |
| 415.009 | 0.516 | 0.510 | 0.509 | 0.512 | 0.004 | 0.719 |
| 409.990 | 0.537 | 0.531 | 0.529 | 0.532 | 0.004 | 0.810 |
| 405.005 | 0.562 | 0.557 | 0.555 | 0.558 | 0.003 | 0.616 |
| 400.016 | 0.595 | 0.590 | 0.589 | 0.591 | 0.003 | 0.579 |

## 3) 2010 WINE

*Table S16 : Absorbance measurements in UVvis (400–800 nm) for 2010 red wine*

| Wavelength (nm) | Absorbance 1 | Absorbance 2 | Absorbance 3 | Mean | Standard deviation | Variation coefficient (%) |
|---|---|---|---|---|---|---|
| 800.003 | 0.042 | 0.044 | 0.044 | 0.043 | 0.001 | 1.719 |
| 794.993 | 0.045 | 0.046 | 0.046 | 0.045 | 0.000 | 0.539 |
| 790.008 | 0.044 | 0.048 | 0.046 | 0.046 | 0.002 | 4.460 |
| 785.014 | 0.046 | 0.046 | 0.047 | 0.046 | 0.001 | 1.675 |
| 780.010 | 0.047 | 0.049 | 0.047 | 0.048 | 0.001 | 2.579 |
| 774.996 | 0.047 | 0.049 | 0.047 | 0.048 | 0.001 | 1.433 |
| 770.008 | 0.047 | 0.051 | 0.050 | 0.049 | 0.002 | 3.678 |
| 765.010 | 0.049 | 0.051 | 0.050 | 0.050 | 0.001 | 2.412 |
| 760.004 | 0.050 | 0.051 | 0.051 | 0.051 | 0.001 | 1.196 |
| 754.988 | 0.050 | 0.052 | 0.053 | 0.051 | 0.002 | 3.132 |
| 749.998 | 0.051 | 0.053 | 0.051 | 0.052 | 0.001 | 2.599 |
| 744.999 | 0.051 | 0.052 | 0.052 | 0.052 | 0.001 | 1.723 |
| 739.992 | 0.051 | 0.053 | 0.052 | 0.052 | 0.001 | 2.078 |
| 735.011 | 0.050 | 0.052 | 0.052 | 0.051 | 0.001 | 2.755 |
| 729.986 | 0.049 | 0.051 | 0.050 | 0.050 | 0.001 | 2.198 |
| 724.988 | 0.047 | 0.050 | 0.048 | 0.049 | 0.002 | 3.542 |
| 720.017 | 0.045 | 0.048 | 0.047 | 0.047 | 0.002 | 3.837 |
| 715.002 | 0.045 | 0.047 | 0.046 | 0.046 | 0.001 | 2.892 |
| 710.014 | 0.046 | 0.048 | 0.049 | 0.048 | 0.001 | 3.090 |
| 705.018 | 0.050 | 0.053 | 0.052 | 0.052 | 0.001 | 2.412 |
| 700.014 | 0.055 | 0.059 | 0.058 | 0.057 | 0.002 | 2.918 |
| 695.001 | 0.062 | 0.064 | 0.064 | 0.063 | 0.001 | 2.166 |
| 690.017 | 0.068 | 0.070 | 0.069 | 0.069 | 0.001 | 2.025 |
| 684.989 | 0.074 | 0.077 | 0.076 | 0.076 | 0.002 | 2.678 |
| 679.989 | 0.079 | 0.082 | 0.081 | 0.081 | 0.001 | 1.756 |
| 675.017 | 0.085 | 0.088 | 0.087 | 0.086 | 0.002 | 1.765 |

| | | | | | | |
|---|---|---|---|---|---|---|
| 670.001 | 0.090 | 0.094 | 0.093 | 0.092 | 0.002 | 2.084 |
| 665.015 | 0.094 | 0.097 | 0.098 | 0.096 | 0.002 | 1.955 |
| 659.984 | 0.101 | 0.104 | 0.103 | 0.103 | 0.002 | 1.657 |
| 654.982 | 0.108 | 0.112 | 0.111 | 0.110 | 0.002 | 1.830 |
| 650.009 | 0.117 | 0.120 | 0.119 | 0.119 | 0.001 | 1.102 |
| 644.993 | 0.126 | 0.129 | 0.130 | 0.128 | 0.002 | 1.634 |
| 640.005 | 0.139 | 0.143 | 0.142 | 0.141 | 0.002 | 1.681 |
| 635.011 | 0.154 | 0.157 | 0.157 | 0.156 | 0.002 | 1.199 |
| 630.010 | 0.170 | 0.174 | 0.174 | 0.173 | 0.002 | 1.266 |
| 625.001 | 0.188 | 0.192 | 0.192 | 0.191 | 0.003 | 1.425 |
| 619.986 | 0.207 | 0.211 | 0.211 | 0.210 | 0.002 | 1.162 |
| 615.001 | 0.229 | 0.234 | 0.234 | 0.232 | 0.003 | 1.294 |
| 610.009 | 0.252 | 0.258 | 0.257 | 0.256 | 0.003 | 1.327 |
| 605.010 | 0.278 | 0.284 | 0.283 | 0.282 | 0.004 | 1.262 |
| 600.005 | 0.303 | 0.309 | 0.309 | 0.307 | 0.003 | 1.115 |
| 594.993 | 0.329 | 0.335 | 0.335 | 0.333 | 0.003 | 1.037 |
| 590.011 | 0.354 | 0.362 | 0.361 | 0.359 | 0.004 | 1.124 |
| 584.987 | 0.380 | 0.386 | 0.387 | 0.384 | 0.004 | 1.061 |
| 579.993 | 0.405 | 0.410 | 0.412 | 0.409 | 0.004 | 0.932 |
| 574.992 | 0.428 | 0.435 | 0.434 | 0.432 | 0.004 | 0.874 |
| 569.986 | 0.448 | 0.456 | 0.457 | 0.454 | 0.005 | 1.070 |
| 565.010 | 0.468 | 0.476 | 0.477 | 0.474 | 0.005 | 1.020 |
| 559.992 | 0.489 | 0.496 | 0.497 | 0.494 | 0.004 | 0.894 |
| 555.005 | 0.508 | 0.518 | 0.517 | 0.514 | 0.005 | 1.008 |
| 550.011 | 0.529 | 0.538 | 0.538 | 0.535 | 0.005 | 0.952 |
| 545.012 | 0.549 | 0.558 | 0.559 | 0.555 | 0.005 | 0.954 |
| 540.007 | 0.568 | 0.577 | 0.577 | 0.574 | 0.005 | 0.929 |
| 534.997 | 0.585 | 0.594 | 0.596 | 0.592 | 0.006 | 1.016 |
| 530.018 | 0.600 | 0.610 | 0.611 | 0.607 | 0.006 | 1.016 |
| 524.996 | 0.614 | 0.624 | 0.625 | 0.621 | 0.006 | 0.929 |

| | | | | | | |
|---|---|---|---|---|---|---|
| 520.007 | 0.625 | 0.634 | 0.636 | 0.631 | 0.006 | 0.916 |
| 515.011 | 0.632 | 0.641 | 0.642 | 0.639 | 0.006 | 0.899 |
| 510.011 | 0.637 | 0.646 | 0.647 | 0.643 | 0.006 | 0.895 |
| 505.005 | 0.638 | 0.648 | 0.649 | 0.645 | 0.006 | 0.924 |
| 499.994 | 0.639 | 0.648 | 0.650 | 0.646 | 0.006 | 0.877 |
| 495.015 | 0.640 | 0.649 | 0.651 | 0.647 | 0.006 | 0.913 |
| 489.994 | 0.640 | 0.649 | 0.651 | 0.647 | 0.006 | 0.948 |
| 485.005 | 0.639 | 0.649 | 0.651 | 0.646 | 0.006 | 1.005 |
| 480.012 | 0.641 | 0.650 | 0.651 | 0.648 | 0.006 | 0.914 |
| 475.013 | 0.641 | 0.651 | 0.652 | 0.648 | 0.006 | 0.905 |
| 470.009 | 0.644 | 0.652 | 0.654 | 0.650 | 0.006 | 0.885 |
| 465.001 | 0.647 | 0.656 | 0.658 | 0.654 | 0.006 | 0.895 |
| 459.988 | 0.649 | 0.659 | 0.660 | 0.656 | 0.006 | 0.925 |
| 455.008 | 0.653 | 0.662 | 0.664 | 0.660 | 0.006 | 0.866 |
| 449.986 | 0.657 | 0.666 | 0.668 | 0.663 | 0.006 | 0.891 |
| 444.997 | 0.660 | 0.670 | 0.671 | 0.667 | 0.006 | 0.891 |
| 440.003 | 0.664 | 0.673 | 0.675 | 0.671 | 0.006 | 0.909 |
| 435.006 | 0.667 | 0.677 | 0.678 | 0.674 | 0.006 | 0.916 |
| 430.003 | 0.672 | 0.682 | 0.683 | 0.679 | 0.006 | 0.896 |
| 424.997 | 0.679 | 0.689 | 0.692 | 0.687 | 0.006 | 0.938 |
| 419.986 | 0.694 | 0.704 | 0.706 | 0.702 | 0.006 | 0.906 |
| 415.009 | 0.717 | 0.728 | 0.730 | 0.725 | 0.007 | 0.993 |
| 409.990 | 0.751 | 0.766 | 0.766 | 0.761 | 0.008 | 1.081 |
| 405.005 | 0.795 | 0.808 | 0.808 | 0.804 | 0.007 | 0.897 |
| 400.016 | 0.847 | 0.861 | 0.864 | 0.858 | 0.009 | 1.042 |

## 4) 2018 WINE – HEAT TEST (60°C)

*Table S17 : Absorbance measurements in UVvis (400–800 nm) for 2018 red wine at 60 °C*

| Wavelength (nm) | Absorbance 1 | Absorbance 2 | Absorbance 3 | Mean | Standard deviation | Variation coefficient (%) |
|---|---|---|---|---|---|---|
| 800.003 | 0.007 | 0.008 | 0.009 | 0.008 | 0.001 | 10.804 |
| 794.993 | 0.007 | 0.008 | 0.009 | 0.008 | 0.001 | 10.823 |
| 790.008 | 0.009 | 0.009 | 0.007 | 0.009 | 0.001 | 11.147 |
| 785.014 | 0.009 | 0.008 | 0.008 | 0.008 | 0.001 | 6.400 |
| 780.010 | 0.009 | 0.010 | 0.011 | 0.010 | 0.001 | 9.789 |
| 774.996 | 0.010 | 0.011 | 0.011 | 0.011 | 0.000 | 4.541 |
| 770.008 | 0.011 | 0.012 | 0.011 | 0.011 | 0.000 | 4.141 |
| 765.010 | 0.011 | 0.011 | 0.011 | 0.011 | 0.000 | 2.514 |
| 760.004 | 0.013 | 0.012 | 0.012 | 0.012 | 0.000 | 2.464 |
| 754.988 | 0.013 | 0.012 | 0.012 | 0.012 | 0.000 | 2.997 |
| 749.998 | 0.013 | 0.012 | 0.012 | 0.013 | 0.001 | 5.102 |
| 744.999 | 0.012 | 0.013 | 0.012 | 0.012 | 0.001 | 6.279 |
| 739.992 | 0.013 | 0.012 | 0.012 | 0.012 | 0.000 | 4.158 |
| 735.011 | 0.011 | 0.011 | 0.010 | 0.011 | 0.001 | 5.940 |
| 729.986 | 0.010 | 0.010 | 0.009 | 0.010 | 0.000 | 3.207 |
| 724.988 | 0.008 | 0.007 | 0.007 | 0.008 | 0.000 | 5.904 |
| 720.017 | 0.006 | 0.006 | 0.005 | 0.006 | 0.000 | 5.019 |
| 715.002 | 0.005 | 0.005 | 0.004 | 0.005 | 0.000 | 3.806 |
| 710.014 | 0.006 | 0.006 | 0.006 | 0.006 | 0.000 | 2.489 |
| 705.018 | 0.010 | 0.010 | 0.010 | 0.010 | 0.000 | 1.515 |
| 700.014 | 0.016 | 0.015 | 0.016 | 0.016 | 0.000 | 0.757 |
| 695.001 | 0.022 | 0.022 | 0.021 | 0.022 | 0.000 | 1.630 |
| 690.017 | 0.027 | 0.028 | 0.027 | 0.027 | 0.001 | 1.948 |
| 684.989 | 0.034 | 0.033 | 0.033 | 0.033 | 0.001 | 1.727 |
| 679.989 | 0.039 | 0.039 | 0.039 | 0.039 | 0.000 | 0.747 |
| 675.017 | 0.044 | 0.044 | 0.044 | 0.044 | 0.000 | 0.106 |
| 670.001 | 0.050 | 0.050 | 0.050 | 0.050 | 0.000 | 0.284 |

| | | | | | | |
|---|---|---|---|---|---|---|
| 665.015 | 0.055 | 0.056 | 0.055 | 0.056 | 0.000 | 0.586 |
| 659.984 | 0.062 | 0.062 | 0.062 | 0.062 | 0.000 | 0.313 |
| 654.982 | 0.070 | 0.071 | 0.071 | 0.070 | 0.000 | 0.618 |
| 650.009 | 0.080 | 0.081 | 0.081 | 0.081 | 0.000 | 0.565 |
| 644.993 | 0.092 | 0.092 | 0.093 | 0.092 | 0.000 | 0.190 |
| 640.005 | 0.107 | 0.107 | 0.108 | 0.107 | 0.000 | 0.314 |
| 635.011 | 0.124 | 0.124 | 0.124 | 0.124 | 0.000 | 0.282 |
| 630.010 | 0.143 | 0.143 | 0.143 | 0.143 | 0.000 | 0.180 |
| 625.001 | 0.160 | 0.162 | 0.162 | 0.162 | 0.001 | 0.603 |
| 619.986 | 0.180 | 0.180 | 0.180 | 0.180 | 0.000 | 0.152 |
| 615.001 | 0.202 | 0.204 | 0.203 | 0.203 | 0.001 | 0.527 |
| 610.009 | 0.225 | 0.228 | 0.227 | 0.226 | 0.001 | 0.509 |
| 605.010 | 0.253 | 0.253 | 0.254 | 0.253 | 0.001 | 0.356 |
| 600.005 | 0.282 | 0.283 | 0.282 | 0.282 | 0.001 | 0.336 |
| 594.993 | 0.312 | 0.315 | 0.314 | 0.314 | 0.001 | 0.357 |
| 590.011 | 0.347 | 0.349 | 0.349 | 0.348 | 0.001 | 0.280 |
| 584.987 | 0.383 | 0.385 | 0.386 | 0.385 | 0.001 | 0.305 |
| 579.993 | 0.421 | 0.424 | 0.423 | 0.422 | 0.001 | 0.322 |
| 574.992 | 0.456 | 0.460 | 0.458 | 0.458 | 0.002 | 0.411 |
| 569.986 | 0.490 | 0.492 | 0.493 | 0.492 | 0.001 | 0.303 |
| 565.010 | 0.517 | 0.522 | 0.522 | 0.521 | 0.003 | 0.520 |
| 559.992 | 0.547 | 0.551 | 0.550 | 0.549 | 0.002 | 0.401 |
| 555.005 | 0.574 | 0.576 | 0.578 | 0.576 | 0.002 | 0.365 |
| 550.011 | 0.597 | 0.600 | 0.600 | 0.599 | 0.002 | 0.319 |
| 545.012 | 0.617 | 0.622 | 0.622 | 0.620 | 0.003 | 0.433 |
| 540.007 | 0.633 | 0.639 | 0.638 | 0.637 | 0.003 | 0.506 |
| 534.997 | 0.649 | 0.652 | 0.653 | 0.651 | 0.002 | 0.332 |
| 530.018 | 0.656 | 0.661 | 0.662 | 0.660 | 0.003 | 0.444 |
| 524.996 | 0.661 | 0.665 | 0.666 | 0.664 | 0.002 | 0.367 |
| 520.007 | 0.660 | 0.666 | 0.666 | 0.664 | 0.003 | 0.510 |

| | | | | | | |
|---|---|---|---|---|---|---|
| 515.011 | 0.657 | 0.661 | 0.662 | 0.660 | 0.003 | 0.401 |
| 510.011 | 0.649 | 0.654 | 0.654 | 0.652 | 0.003 | 0.449 |
| 505.005 | 0.639 | 0.643 | 0.644 | 0.642 | 0.003 | 0.418 |
| 499.994 | 0.629 | 0.632 | 0.633 | 0.631 | 0.002 | 0.352 |
| 495.015 | 0.618 | 0.622 | 0.622 | 0.621 | 0.002 | 0.390 |
| 489.994 | 0.608 | 0.612 | 0.612 | 0.611 | 0.003 | 0.420 |
| 485.005 | 0.599 | 0.603 | 0.602 | 0.601 | 0.002 | 0.396 |
| 480.012 | 0.591 | 0.595 | 0.595 | 0.594 | 0.002 | 0.362 |
| 475.013 | 0.584 | 0.588 | 0.588 | 0.586 | 0.002 | 0.415 |
| 470.009 | 0.579 | 0.582 | 0.583 | 0.581 | 0.002 | 0.398 |
| 465.001 | 0.576 | 0.580 | 0.579 | 0.578 | 0.002 | 0.352 |
| 459.988 | 0.573 | 0.577 | 0.576 | 0.575 | 0.002 | 0.367 |
| 455.008 | 0.570 | 0.574 | 0.573 | 0.572 | 0.002 | 0.371 |
| 449.986 | 0.566 | 0.569 | 0.569 | 0.568 | 0.002 | 0.390 |
| 444.997 | 0.561 | 0.565 | 0.565 | 0.564 | 0.002 | 0.384 |
| 440.003 | 0.557 | 0.560 | 0.560 | 0.559 | 0.002 | 0.322 |
| 435.006 | 0.553 | 0.556 | 0.556 | 0.555 | 0.002 | 0.324 |
| 430.003 | 0.549 | 0.552 | 0.552 | 0.551 | 0.002 | 0.311 |
| 424.997 | 0.548 | 0.550 | 0.550 | 0.549 | 0.002 | 0.292 |
| 419.986 | 0.552 | 0.554 | 0.554 | 0.554 | 0.002 | 0.281 |
| 415.009 | 0.563 | 0.566 | 0.566 | 0.565 | 0.001 | 0.259 |
| 409.990 | 0.583 | 0.586 | 0.585 | 0.584 | 0.001 | 0.239 |
| 405.005 | 0.611 | 0.613 | 0.613 | 0.612 | 0.001 | 0.221 |
| 400.016 | 0.647 | 0.650 | 0.649 | 0.649 | 0.001 | 0.199 |

## 5) 2018 WINE - LACCASE TEST

*Table S18 : Absorbance measurements in UVvis (400–800 nm) for 2018 red wine – laccase oxidation test*

| Wavelength (nm) | Absorbance 1 | Absorbance 2 | Absorbance 3 | Mean | Standard deviation | Variation coefficient (%) |
|---|---|---|---|---|---|---|

| | | | | | | |
|---|---|---|---|---|---|---|
| 800.003 | 0.018 | 0.018 | 0.014 | 0.016 | 0.002 | 14.246 |
| 794.993 | 0.020 | 0.019 | 0.014 | 0.018 | 0.003 | 18.253 |
| 790.008 | 0.021 | 0.021 | 0.014 | 0.019 | 0.004 | 19.949 |
| 785.014 | 0.020 | 0.020 | 0.017 | 0.019 | 0.002 | 8.601 |
| 780.010 | 0.022 | 0.021 | 0.016 | 0.020 | 0.003 | 14.961 |
| 774.996 | 0.023 | 0.021 | 0.016 | 0.020 | 0.004 | 19.560 |
| 770.008 | 0.023 | 0.024 | 0.018 | 0.022 | 0.004 | 16.252 |
| 765.010 | 0.024 | 0.023 | 0.018 | 0.022 | 0.003 | 15.845 |
| 760.004 | 0.025 | 0.025 | 0.019 | 0.023 | 0.003 | 13.380 |
| 754.988 | 0.025 | 0.024 | 0.019 | 0.023 | 0.003 | 14.263 |
| 749.998 | 0.027 | 0.025 | 0.019 | 0.024 | 0.004 | 16.409 |
| 744.999 | 0.026 | 0.026 | 0.020 | 0.024 | 0.004 | 16.017 |
| 739.992 | 0.026 | 0.027 | 0.020 | 0.024 | 0.003 | 14.162 |
| 735.011 | 0.026 | 0.026 | 0.020 | 0.024 | 0.003 | 14.379 |
| 729.986 | 0.024 | 0.024 | 0.018 | 0.022 | 0.004 | 16.194 |
| 724.988 | 0.023 | 0.022 | 0.016 | 0.021 | 0.004 | 19.417 |
| 720.017 | 0.021 | 0.021 | 0.014 | 0.019 | 0.004 | 20.701 |
| 715.002 | 0.020 | 0.021 | 0.014 | 0.019 | 0.004 | 21.291 |
| 710.014 | 0.023 | 0.023 | 0.016 | 0.021 | 0.004 | 20.438 |
| 705.018 | 0.027 | 0.028 | 0.021 | 0.025 | 0.004 | 15.594 |
| 700.014 | 0.033 | 0.034 | 0.026 | 0.031 | 0.004 | 14.418 |
| 695.001 | 0.040 | 0.040 | 0.033 | 0.038 | 0.004 | 11.623 |
| 690.017 | 0.046 | 0.046 | 0.039 | 0.044 | 0.004 | 9.241 |
| 684.989 | 0.052 | 0.053 | 0.045 | 0.050 | 0.005 | 9.288 |
| 679.989 | 0.059 | 0.059 | 0.052 | 0.057 | 0.004 | 7.630 |
| 675.017 | 0.066 | 0.066 | 0.057 | 0.063 | 0.005 | 7.873 |
| 670.001 | 0.072 | 0.073 | 0.064 | 0.070 | 0.005 | 7.175 |
| 665.015 | 0.079 | 0.079 | 0.070 | 0.076 | 0.005 | 7.105 |
| 659.984 | 0.086 | 0.087 | 0.077 | 0.084 | 0.006 | 6.946 |
| 654.982 | 0.097 | 0.097 | 0.087 | 0.094 | 0.006 | 6.512 |

| | | | | | | |
|---|---|---|---|---|---|---|
| 650.009 | 0.108 | 0.109 | 0.099 | 0.105 | 0.006 | 5.398 |
| 644.993 | 0.123 | 0.124 | 0.112 | 0.120 | 0.007 | 5.446 |
| 640.005 | 0.140 | 0.142 | 0.128 | 0.137 | 0.007 | 5.353 |
| 635.011 | 0.160 | 0.161 | 0.148 | 0.156 | 0.008 | 4.898 |
| 630.010 | 0.181 | 0.183 | 0.168 | 0.178 | 0.008 | 4.534 |
| 625.001 | 0.205 | 0.206 | 0.189 | 0.200 | 0.009 | 4.668 |
| 619.986 | 0.227 | 0.229 | 0.212 | 0.223 | 0.009 | 4.257 |
| 615.001 | 0.253 | 0.253 | 0.236 | 0.247 | 0.010 | 4.096 |
| 610.009 | 0.280 | 0.283 | 0.263 | 0.276 | 0.011 | 3.917 |
| 605.010 | 0.313 | 0.314 | 0.293 | 0.307 | 0.012 | 3.873 |
| 600.005 | 0.346 | 0.349 | 0.325 | 0.340 | 0.013 | 3.819 |
| 594.993 | 0.383 | 0.385 | 0.360 | 0.376 | 0.014 | 3.625 |
| 590.011 | 0.424 | 0.425 | 0.399 | 0.416 | 0.015 | 3.602 |
| 584.987 | 0.467 | 0.470 | 0.441 | 0.459 | 0.016 | 3.509 |
| 579.993 | 0.512 | 0.515 | 0.484 | 0.503 | 0.017 | 3.416 |
| 574.992 | 0.553 | 0.556 | 0.524 | 0.544 | 0.018 | 3.259 |
| 569.986 | 0.594 | 0.596 | 0.562 | 0.584 | 0.019 | 3.237 |
| 565.010 | 0.626 | 0.631 | 0.593 | 0.617 | 0.020 | 3.295 |
| 559.992 | 0.662 | 0.666 | 0.624 | 0.650 | 0.023 | 3.595 |
| 555.005 | 0.689 | 0.694 | 0.654 | 0.679 | 0.022 | 3.233 |
| 550.011 | 0.718 | 0.721 | 0.679 | 0.706 | 0.024 | 3.339 |
| 545.012 | 0.740 | 0.745 | 0.702 | 0.729 | 0.024 | 3.239 |
| 540.007 | 0.758 | 0.762 | 0.719 | 0.746 | 0.024 | 3.193 |
| 534.997 | 0.772 | 0.777 | 0.733 | 0.761 | 0.025 | 3.221 |
| 530.018 | 0.782 | 0.786 | 0.741 | 0.770 | 0.025 | 3.207 |
| 524.996 | 0.784 | 0.789 | 0.744 | 0.773 | 0.025 | 3.213 |
| 520.007 | 0.781 | 0.787 | 0.741 | 0.770 | 0.025 | 3.227 |
| 515.011 | 0.775 | 0.778 | 0.735 | 0.763 | 0.024 | 3.192 |
| 510.011 | 0.763 | 0.767 | 0.724 | 0.751 | 0.023 | 3.122 |
| 505.005 | 0.748 | 0.752 | 0.709 | 0.736 | 0.024 | 3.223 |

| | | | | | | |
|---|---|---|---|---|---|---|
| 499.994 | 0.732 | 0.737 | 0.696 | 0.722 | 0.022 | 3.083 |
| 495.015 | 0.719 | 0.722 | 0.683 | 0.708 | 0.022 | 3.068 |
| 489.994 | 0.705 | 0.709 | 0.670 | 0.695 | 0.021 | 3.072 |
| 485.005 | 0.693 | 0.696 | 0.659 | 0.682 | 0.021 | 3.038 |
| 480.012 | 0.683 | 0.686 | 0.649 | 0.673 | 0.021 | 3.083 |
| 475.013 | 0.674 | 0.677 | 0.640 | 0.664 | 0.020 | 3.089 |
| 470.009 | 0.667 | 0.670 | 0.634 | 0.657 | 0.020 | 3.029 |
| 465.001 | 0.663 | 0.666 | 0.630 | 0.653 | 0.020 | 3.032 |
| 459.988 | 0.659 | 0.662 | 0.627 | 0.649 | 0.019 | 2.939 |
| 455.008 | 0.655 | 0.658 | 0.624 | 0.646 | 0.019 | 2.950 |
| 449.986 | 0.651 | 0.653 | 0.619 | 0.641 | 0.019 | 2.962 |
| 444.997 | 0.646 | 0.649 | 0.615 | 0.636 | 0.019 | 2.980 |
| 440.003 | 0.641 | 0.644 | 0.610 | 0.631 | 0.019 | 2.975 |
| 435.006 | 0.635 | 0.638 | 0.604 | 0.626 | 0.019 | 2.976 |
| 430.003 | 0.631 | 0.633 | 0.600 | 0.621 | 0.019 | 3.001 |
| 424.997 | 0.627 | 0.630 | 0.596 | 0.618 | 0.019 | 3.030 |
| 419.986 | 0.630 | 0.632 | 0.598 | 0.620 | 0.019 | 3.118 |
| 415.009 | 0.639 | 0.642 | 0.607 | 0.629 | 0.020 | 3.120 |
| 409.990 | 0.659 | 0.659 | 0.623 | 0.647 | 0.021 | 3.202 |
| 405.005 | 0.684 | 0.685 | 0.648 | 0.672 | 0.021 | 3.159 |
| 400.016 | 0.720 | 0.720 | 0.680 | 0.707 | 0.023 | 3.275 |

## 6) 2018 WINE - HYDROGEN PEROXIDE TEST

*Table S19 : Absorbance measurements in UVvis (400–800 nm) for 2018 red wine – hydrogen peroxide oxidation test*

| Wavelength (nm) | Absorbance 1 | Absorbance 2 | Absorbance 3 | Mean | Standard deviation | Variation coefficient (%) |
|---|---|---|---|---|---|---|
| 800.00250 | 0.00777 | 0.00729 | 0.00722 | 0.00743 | 0.00030 | 4.01531 |

| | | | | | | |
|---|---|---|---|---|---|---|
| 794.99286 | 0.00778 | 0.00788 | 0.00990 | 0.00852 | 0.00120 | 14.05238 |
| 790.00812 | 0.00683 | 0.00782 | 0.01007 | 0.00824 | 0.00166 | 20.14709 |
| 785.01367 | 0.00862 | 0.00804 | 0.01036 | 0.00900 | 0.00121 | 13.39701 |
| 780.00964 | 0.01067 | 0.00958 | 0.00949 | 0.00991 | 0.00066 | 6.62254 |
| 774.99597 | 0.01066 | 0.00877 | 0.01172 | 0.01038 | 0.00149 | 14.38445 |
| 770.00787 | 0.01008 | 0.00995 | 0.01124 | 0.01042 | 0.00071 | 6.80274 |
| 765.01044 | 0.01012 | 0.00997 | 0.01025 | 0.01011 | 0.00014 | 1.40427 |
| 760.00378 | 0.01084 | 0.01012 | 0.01166 | 0.01087 | 0.00077 | 7.07063 |
| 754.98773 | 0.01169 | 0.01044 | 0.01165 | 0.01126 | 0.00071 | 6.32143 |
| 749.99811 | 0.01180 | 0.00975 | 0.01297 | 0.01151 | 0.00163 | 14.13336 |
| 744.99945 | 0.01023 | 0.00939 | 0.01070 | 0.01011 | 0.00066 | 6.54654 |
| 739.99188 | 0.00939 | 0.00910 | 0.00979 | 0.00943 | 0.00035 | 3.66648 |
| 735.01105 | 0.00813 | 0.00817 | 0.00887 | 0.00839 | 0.00042 | 4.97048 |
| 729.98584 | 0.00659 | 0.00465 | 0.00758 | 0.00627 | 0.00149 | 23.72833 |
| 724.98767 | 0.00430 | 0.00288 | 0.00382 | 0.00367 | 0.00072 | 19.64157 |
| 720.01666 | 0.00186 | 0.00040 | 0.00208 | 0.00145 | 0.00092 | 63.39425 |
| 715.00153 | 0.00012 | -0.00045 | 0.00027 | -0.00002 | 0.00038 | -2055.94762 |
| 710.01373 | 0.00058 | -0.00014 | 0.00127 | 0.00057 | 0.00070 | 123.43076 |
| 705.01782 | 0.00402 | 0.00303 | 0.00461 | 0.00389 | 0.00080 | 20.57916 |
| 700.01361 | 0.00862 | 0.00763 | 0.00915 | 0.00847 | 0.00077 | 9.13066 |
| 695.00128 | 0.01428 | 0.01289 | 0.01400 | 0.01372 | 0.00073 | 5.33136 |
| 690.01703 | 0.01826 | 0.01732 | 0.01817 | 0.01792 | 0.00052 | 2.88749 |
| 684.98865 | 0.02264 | 0.02131 | 0.02346 | 0.02247 | 0.00108 | 4.81859 |
| 679.98853 | 0.02751 | 0.02588 | 0.02728 | 0.02689 | 0.00089 | 3.29255 |
| 675.01697 | 0.03073 | 0.02928 | 0.03147 | 0.03049 | 0.00111 | 3.65569 |
| 670.00140 | 0.03478 | 0.03349 | 0.03417 | 0.03415 | 0.00064 | 1.88881 |
| 665.01459 | 0.03699 | 0.03489 | 0.03582 | 0.03590 | 0.00105 | 2.93592 |
| 659.98376 | 0.03974 | 0.03783 | 0.03915 | 0.03891 | 0.00098 | 2.51065 |
| 654.98187 | 0.04371 | 0.04189 | 0.04251 | 0.04271 | 0.00092 | 2.16384 |
| 650.00922 | 0.04700 | 0.04460 | 0.04602 | 0.04587 | 0.00121 | 2.62821 |

| | | | | | | |
|---|---|---|---|---|---|---|
| 644.99261 | 0.05218 | 0.04873 | 0.05068 | 0.05053 | 0.00173 | 3.42368 |
| 640.00549 | 0.05747 | 0.05480 | 0.05630 | 0.05619 | 0.00133 | 2.37505 |
| 635.01123 | 0.06439 | 0.06155 | 0.06270 | 0.06288 | 0.00143 | 2.27573 |
| 630.00989 | 0.07123 | 0.06808 | 0.06956 | 0.06962 | 0.00157 | 2.25706 |
| 625.00146 | 0.07962 | 0.07559 | 0.07647 | 0.07723 | 0.00212 | 2.74526 |
| 619.98621 | 0.08723 | 0.08335 | 0.08509 | 0.08522 | 0.00194 | 2.28017 |
| 615.00085 | 0.09649 | 0.09261 | 0.09369 | 0.09426 | 0.00200 | 2.12241 |
| 610.00885 | 0.10705 | 0.10156 | 0.10291 | 0.10384 | 0.00286 | 2.75188 |
| 605.01013 | 0.11726 | 0.11159 | 0.11321 | 0.11402 | 0.00292 | 2.55780 |
| 600.00476 | 0.12797 | 0.12234 | 0.12336 | 0.12455 | 0.00300 | 2.40756 |
| 594.99286 | 0.13816 | 0.13184 | 0.13308 | 0.13436 | 0.00335 | 2.49104 |
| 590.01147 | 0.14946 | 0.14329 | 0.14442 | 0.14572 | 0.00328 | 2.25422 |
| 584.98663 | 0.16099 | 0.15377 | 0.15564 | 0.15680 | 0.00375 | 2.39127 |
| 579.99255 | 0.17346 | 0.16527 | 0.16670 | 0.16848 | 0.00437 | 2.59611 |
| 574.99231 | 0.18444 | 0.17583 | 0.17699 | 0.17908 | 0.00467 | 2.60863 |
| 569.98578 | 0.19598 | 0.18675 | 0.18783 | 0.19019 | 0.00504 | 2.65219 |
| 565.01050 | 0.20705 | 0.19746 | 0.19883 | 0.20111 | 0.00519 | 2.58109 |
| 559.99176 | 0.21909 | 0.20922 | 0.21057 | 0.21296 | 0.00535 | 2.51209 |
| 555.00452 | 0.23266 | 0.22151 | 0.22340 | 0.22586 | 0.00597 | 2.64183 |
| 550.01129 | 0.24565 | 0.23474 | 0.23639 | 0.23893 | 0.00588 | 2.46234 |
| 545.01221 | 0.26018 | 0.24810 | 0.24969 | 0.25266 | 0.00656 | 2.59703 |
| 540.00732 | 0.27393 | 0.26117 | 0.26309 | 0.26606 | 0.00688 | 2.58490 |
| 534.99677 | 0.28766 | 0.27490 | 0.27608 | 0.27955 | 0.00705 | 2.52133 |
| 530.01813 | 0.30171 | 0.28766 | 0.28966 | 0.29301 | 0.00760 | 2.59269 |
| 524.99628 | 0.31505 | 0.30148 | 0.30273 | 0.30642 | 0.00750 | 2.44835 |
| 520.00653 | 0.32693 | 0.31332 | 0.31519 | 0.31848 | 0.00738 | 2.31712 |
| 515.01141 | 0.33660 | 0.32304 | 0.32498 | 0.32821 | 0.00733 | 2.23378 |
| 510.01089 | 0.34462 | 0.33148 | 0.33320 | 0.33643 | 0.00715 | 2.12389 |
| 505.00497 | 0.35022 | 0.33686 | 0.33888 | 0.34199 | 0.00720 | 2.10609 |
| 499.99380 | 0.35485 | 0.34239 | 0.34425 | 0.34717 | 0.00672 | 1.93661 |

| | | | | | | |
|---|---|---|---|---|---|---|
| 495.01538 | 0.36008 | 0.34738 | 0.34902 | 0.35216 | 0.00690 | 1.96064 |
| 489.99380 | 0.36385 | 0.35175 | 0.35325 | 0.35628 | 0.00660 | 1.85107 |
| 485.00519 | 0.36705 | 0.35532 | 0.35705 | 0.35981 | 0.00634 | 1.76069 |
| 480.01151 | 0.37163 | 0.35985 | 0.36204 | 0.36451 | 0.00626 | 1.71806 |
| 475.01291 | 0.37534 | 0.36409 | 0.36576 | 0.36840 | 0.00607 | 1.64692 |
| 470.00946 | 0.38017 | 0.36898 | 0.37062 | 0.37326 | 0.00604 | 1.61815 |
| 465.00101 | 0.38584 | 0.37442 | 0.37652 | 0.37893 | 0.00608 | 1.60329 |
| 459.98788 | 0.39128 | 0.38033 | 0.38247 | 0.38469 | 0.00580 | 1.50853 |
| 455.00827 | 0.39696 | 0.38590 | 0.38821 | 0.39036 | 0.00584 | 1.49491 |
| 449.98569 | 0.40172 | 0.39112 | 0.39284 | 0.39522 | 0.00569 | 1.43923 |
| 444.99686 | 0.40690 | 0.39626 | 0.39794 | 0.40036 | 0.00572 | 1.42882 |
| 440.00345 | 0.41135 | 0.40076 | 0.40311 | 0.40507 | 0.00556 | 1.37254 |
| 435.00555 | 0.41621 | 0.40482 | 0.40717 | 0.40940 | 0.00602 | 1.46953 |
| 430.00333 | 0.42150 | 0.41063 | 0.41269 | 0.41494 | 0.00578 | 1.39223 |
| 424.99667 | 0.42888 | 0.41804 | 0.41996 | 0.42229 | 0.00578 | 1.36946 |
| 419.98572 | 0.44114 | 0.43019 | 0.43235 | 0.43456 | 0.00580 | 1.33522 |
| 415.00906 | 0.45843 | 0.44769 | 0.44942 | 0.45184 | 0.00577 | 1.27705 |
| 409.98962 | 0.48360 | 0.47181 | 0.47384 | 0.47642 | 0.00630 | 1.32306 |
| 405.00467 | 0.51289 | 0.50178 | 0.50356 | 0.50608 | 0.00597 | 1.17948 |
| 400.01563 | 0.55027 | 0.53887 | 0.54032 | 0.54315 | 0.00621 | 1.14305 |

# IV. HIGH RESOLUTION UPLC-MS

## 1) 2018 WINE

*Table S20 : High resolution UPLC-MS for 2018 red wine*

| $[M+H]^+$ | | 2018 n1 | 2018 n2 | 2018 n3 | Mean | Standard deviation | Variation coefficient (%) |
|---|---|---|---|---|---|---|---|
| 287 | Exact mass | 287.0885 | 287.0885 | 287.0885 | 287.0885 | | |
| | Intensity | 6.55E+05 | 6.01E+05 | 5.92E+05 | 616000 | 34035.7165 | 5.5 |
| 289 | Exact mass | 289.1032 | 289.1032 | 289.1032 | 289.1032 | | |
| | Intensity | 2.20E+05 | 2.08E+05 | 2.03E+05 | 210100 | 9180.41393 | 4.4 |
| 291 | Exact mass | 291.125 | 291.1176 | 291.1176 | 291.120067 | | |
| | Intensity | 2.35E+05 | 2.25E+05 | 2.28E+05 | 229300 | 5271.62214 | 2.3 |
| 303 | Exact mass | 303.0846 | 303.0846 | 303.0846 | 303.0846 | | |
| | Intensity | 6.19E+06 | 5.72E+06 | 5.59E+06 | 5830666.67 | 313911.346 | 5.4 |
| 317 | Exact mass | 317.1007 | 317.1007 | 317.1007 | 317.1007 | | |
| | Intensity | 4.42E+06 | 4.17E+06 | 4.15E+06 | 4247333.33 | 148288.008 | 3.5 |
| 319 | Exact mass | 319.0854 | 319.0854 | 319.0854 | 319.0854 | | |
| | Intensity | 1.96E+06 | 1.75E+06 | 1.67E+06 | 1790333.33 | 147842.258 | 8.3 |
| 331 | Exact mass | 331.1239 | 331.1239 | 331.1239 | 331.1239 | | |
| | Intensity | 1.14E+07 | 1.02E+07 | 9.51E+06 | 10389000 | 977518.798 | 9.4 |
| 333 | Exact mass | 333.1042 | 333.1042 | 333.0962 | 333.101533 | | |
| | Intensity | 1.20E+06 | 1.05E+06 | 1.05E+06 | 1098666.67 | 89511.638 | 8.1 |
| 347 | Exact mass | 347.1152 | 347.1152 | 347.1152 | 347.1152 | | |
| | Intensity | 6.76E+05 | 6.41E+05 | 6.35E+05 | 650666.667 | 22250.0187 | 3.4 |
| 409 | Exact mass | 409.1469 | 409.1381 | 409.1381 | 409.141033 | | |
| | Intensity | 4.68E+05 | 4.41E+05 | 4.59E+05 | 456266.667 | 13770.3788 | 3.0 |
| 493 | Exact mass | 493.1945 | 493.1945 | 493.1945 | 493.1945 | | |
| | Intensity | 4.21E+06 | 4.03E+06 | 4.12E+06 | 4117000 | 93016.1276 | 2.3 |
| 535 | Exact mass | 535.2134 | 535.2134 | 535.2134 | 535.2134 | | |

| | | | | | | | |
|---|---|---|---|---|---|---|---|
| | Intensity | 4.10E+06 | 3.79E+06 | 3.73E+06 | 3873000 | 194432.508 | 5.0 |
| 536 | Exact mass | 536.2134 | 536.2134 | 536.2134 | 536.2134 | | |
| | Intensity | 8.93E+05 | 8.55E+05 | 8.59E+05 | 868933.333 | 21001.984 | 2.4 |
| 577 | Exact mass | 577.2081 | 577.2081 | 577.2081 | 577.2081 | | |
| | Intensity | 1.22E+05 | 1.09E+05 | 1.07E+05 | 112533.333 | 7836.02791 | 7.0 |
| 579 | Exact mass | 579.2229 | 579.2229 | 579.2229 | 579.2229 | | |
| | Intensity | 7.68E+05 | 7.23E+05 | 7.07E+05 | 732733.333 | 31665.4912 | 4.3 |
| 581 | Exact mass | 581.2623 | 581.2623 | 581.2623 | 581.2623 | | |
| | Intensity | 2.48E+05 | 2.24E+05 | 2.19E+05 | 230600 | 15429.8412 | 6.7 |
| 639 | Exact mass | 639.2562 | 639.2562 | 639.2562 | 639.2562 | | |
| | Intensity | 3.26E+06 | 2.98E+06 | 2.87E+06 | 3038333.33 | 200253.173 | 6.6 |
| 640 | Exact mass | 640.2607 | 640.2607 | 640.2607 | 640.2607 | | |
| | Intensity | 9.50E+05 | 8.71E+05 | 8.47E+05 | 889433.333 | 53891.867 | 6.1 |
| 867 | Exact mass | 867.3312 | 867.3312 | 867.3312 | 867.3312 | | |
| | Intensity | 3.47E+05 | 3.13E+05 | 3.07E+05 | 321866.667 | 21628.7617 | 6.7 |

## 2) 2014 WINE

*Table S21 : High resolution UPLC-MS for 2014 red wine*

| $[M+H]^+$ | | 2014 n1 | 2014 n2 | 2014 n3 | Mean | Standard deviation | Variation coefficient (%) |
|---|---|---|---|---|---|---|---|
| 287 | Exact mass | 287.0885 | 287.0885 | 287.0885 | 287.0885 | | |
| | Intensity | 1.20E+06 | 1.19E+06 | 1.15E+06 | 1179333.333 | 24785.7486 | 2.1 |

| | | | | | | | |
|---|---|---|---|---|---|---|---|
| 289 | Exact mass | 289.1106 | 289.1032 | 289.1032 | 289.1056667 | | |
| | Intensity | 2.37E+05 | 2.29E+05 | 2.20E+05 | 228700 | 8860.58689 | 3.9 |
| 291 | Exact mass | 291.125 | 291.125 | 291.125 | 291.125 | | |
| | Intensity | 4.31E+05 | 3.79E+05 | 3.54E+05 | 387800 | 39217.8531 | 10.1 |
| 303 | Exact mass | 303.0922 | 303.0846 | 303.0846 | 303.0871333 | | |
| | Intensity | 1.04E+07 | 9.98E+06 | 9.82E+06 | 10070000 | 306196.016 | 3.0 |
| 317 | Exact mass | 317.1085 | 317.1085 | 317.1085 | 317.1085 | | |
| | Intensity | 4.08E+06 | 3.75E+06 | 3.37E+06 | 3733666.667 | 356716.04 | 9.6 |
| 319 | Exact mass | 319.0854 | 319.0854 | 319.0854 | 319.0854 | | |
| | Intensity | 2.10E+06 | 2.02E+06 | 1.87E+06 | 1993000 | 116567.577 | 5.8 |
| 331 | Exact mass | 331.1239 | 331.1239 | 331.1239 | 331.1239 | | |
| | Intensity | 5.68E+05 | 5.31E+05 | 4.95E+05 | 531300 | 36450.1029 | 6.9 |
| 333 | Exact mass | 333.1042 | 333.1042 | 333.1042 | 333.1042 | | |
| | Intensity | 7.89E+05 | 7.46E+05 | 6.90E+05 | 741733.3333 | 49525.3807 | 6.7 |
| 347 | Exact mass | 347.1233 | 347.1233 | 347.1233 | 347.1233 | | |
| | Intensity | 5.90E+05 | 5.65E+05 | 5.09E+05 | 554500 | 41587.0172 | 7.5 |
| 409 | Exact mass | 409.1469 | 409.1469 | 409.1469 | 409.1469 | | |
| | Intensity | 1.07E+06 | 1.02E+06 | 9.35E+05 | 1007033.333 | 67125.2809 | 6.7 |

| | | | | | | | |
|---|---|---|---|---|---|---|---|
| 493 | Exact mass | 493.2043 | 493.2043 | 493.1945 | 493.2010333 | | |
| | Intensity | 2.38E+05 | 2.17E+05 | 2.01E+05 | 218933.3333 | 18461.1303 | 8.4 |
| 535 | Exact mass | 535.2134 | 535.2134 | 535.2134 | 535.2134 | | |
| | Intensity | 7.68E+04 | 7.24E+04 | 6.94E+04 | 72846.66667 | 3730.11171 | 5.1 |
| 536 | Exact mass | 536.2235 | 536.2235 | 536.2235 | 536.2235 | | |
| | Intensity | 2.49E+04 | 2.31E+04 | 2.16E+04 | 23223.33333 | 1626.60177 | 7.0 |
| 577 | Exact mass | 577.2081 | 577.2081 | 577.2081 | 577.2081 | | |
| | Intensity | 8.51E+04 | 8.56E+04 | 8.01E+04 | 83600 | 3009.50162 | 3.6 |
| 579 | Exact mass | 579.2334 | 579.2334 | 579.2229 | 579.2299 | | |
| | Intensity | 7.56E+05 | 6.81E+05 | 6.29E+05 | 688766.6667 | 63430.4606 | 9.2 |
| 581 | Exact mass | 581.2623 | 581.2623 | 581.2623 | 581.2623 | | |
| | Intensity | 2.50E+05 | 2.37E+05 | 2.24E+05 | 236900 | 12852.6262 | 5.4 |
| 639 | Exact mass | 639.2562 | 639.2562 | 639.2562 | 639.2562 | | |
| | Intensity | 1.56E+05 | 1.52E+05 | 1.38E+05 | 148400 | 9681.42551 | 6.5 |
| 640 | Exact mass | 640.2607 | 640.2607 | 640.2607 | 640.2607 | | |
| | Intensity | 5.64E+04 | 5.47E+04 | 5.01E+04 | 53726.66667 | 3269.95923 | 6.1 |
| 867 | Exact mass | 867.3312 | 867.3312 | 867.3312 | 867.3312 | | |
| | Intensity | 2.48E+05 | 2.39E+05 | 5.01E+04 | 179156.6667 | 111875 | 62.4 |

## 3) 2010 WINE

*Table S22 : High resolution UPLC-MS for 2010 red wine*

| $[M+H]^+$ | | 2010 n1 | 2010 n2 | 2010 n3 | Mean | Standard deviation | Variation coefficient (%) |
|---|---|---|---|---|---|---|---|
| 287 | Exact mass | 287.0959 | 287.0959 | 287.0959 | 287.0959 | | |
| | Intensity | 5.66E+06 | 4.48E+06 | 3.56E+06 | 4565333.333 | 1052720.44 | 23.1 |
| 289 | Exact mass | 289.1106 | 289.1106 | 289.1106 | 289.1106 | | |
| | Intensity | 5.51E+05 | 4.53E+05 | 3.78E+05 | 460500 | 86836.801 | 18.9 |
| 291 | Exact mass | 291.1325 | 291.125 | 291.125 | 291.1275 | | |
| | Intensity | 7.33E+05 | 6.05E+05 | 5.61E+05 | 633333.3333 | 89433.8489 | 14.1 |
| 303 | Exact mass | 303.0922 | 303.0922 | 303.0922 | 303.0922 | | |
| | Intensity | 2.90E+07 | 2.37E+07 | 1.92E+07 | 23946666.67 | 4911724.07 | 20.5 |
| 317 | Exact mass | 317.1162 | 317.1085 | 317.1085 | 317.1110667 | | |
| | Intensity | 1.32E+07 | 1.02E+07 | 8.96E+06 | 10766333.33 | 2181797.5 | 20.3 |
| 319 | Exact mass | 319.0932 | 319.0932 | 319.0932 | 319.0932 | | |
| | Intensity | 9.26E+06 | 6.69E+06 | 4.97E+06 | 6974666.667 | 2159915.35 | 31.0 |
| 331 | Exact mass | 331.1318 | 331.1318 | 331.1318 | 331.1318 | | |
| | Intensity | 7.59E+05 | 5.81E+05 | 4.45E+05 | 595200 | 157410.959 | 26.4 |
| 333 | Exact mass | 333.1121 | 333.1121 | 333.1042 | 333.1094667 | | |
| | Intensity | 3.24E+06 | 2.34E+06 | 1.99E+06 | 2524333.333 | 647337.109 | 25.6 |
| 347 | Exact mass | 347.1314 | 347.1314 | 347.1233 | 347.1287 | | |
| | Intensity | 1.99E+06 | 1.46E+06 | 1.29E+06 | 1580000 | 360542.647 | 22.8 |
| 409 | Exact mass | 409.1558 | 409.1558 | 409.1469 | 409.1528333 | | |

| | | | | | | | |
|---|---|---|---|---|---|---|---|
| | Intensity | 1.78E+06 | 1.43E+06 | 1.19E+06 | 1464333.333 | 298352.7 | 20.4 |
| 493 | Exact mass | 493.214 | 493.2043 | 493.2043 | 493.2075333 | | |
| | Intensity | 1.99E+05 | 1.69E+05 | 1.46E+05 | 171100 | 26894.4232 | 15.7 |
| 535 | Exact mass | 535.2236 | 535.2236 | 535.2236 | 535.2236 | | |
| | Intensity | 5.74E+04 | 4.67E+04 | 3.82E+04 | 47410 | 9612.55949 | 20.3 |
| 536 | Exact mass | 536.2336 | 536.2336 | 536.2336 | 536.2336 | | |
| | Intensity | 2.57E+04 | 2.13E+04 | 1.68E+04 | 21246.66667 | 4465.33687 | 21.0 |
| 577 | Exact mass | 577.2291 | 577.2291 | 577.2291 | 577.2291 | | |
| | Intensity | 1.51E+05 | 1.18E+05 | 9.17E+04 | 120243.3333 | 29754.2876 | 24.7 |
| 579 | Exact mass | 579.2439 | 579.2439 | 579.2334 | 579.2404 | | |
| | Intensity | 8.55E+05 | 7.50E+05 | 6.84E+05 | 762933.3333 | 86474.4085 | 11.3 |
| 581 | Exact mass | 581.2833 | 581.2728 | 581.2728 | 581.2763 | | |
| | Intensity | 5.87E+05 | 5.59E+05 | 4.79E+05 | 541933.3333 | 55986.1888 | 10.3 |
| 639 | Exact mass | 639.2673 | 639.2562 | 639.2562 | 639.2599 | | |
| | Intensity | 2.05E+05 | 1.81E+05 | 1.51E+05 | 178800 | 27067.1387 | 15.1 |
| 640 | Exact mass | 640.2717 | 640.2607 | 640.2607 | 640.2644 | | |
| | Intensity | 8.00E+04 | 6.87E+04 | 5.90E+04 | 69246.66667 | 10479.93 | 15.1 |
| 867 | Exact mass | 867.3569 | 867.3440 | 867.3440 | 867.3440 | | |
| | Intensity | 2.81E+05 | 2.40E+05 | 2.09E+05 | 243200 | 35821.2228 | 14.7 |

## 4) 2018 WINE – HEAT TEST (60 °C)

*Table S23 : High resolution UPLC-MS for 2018 wine at 60 °C*

| [M+H]+ | | 2018 60°C n1 | 2018 60°C n2 | 2018 60°C n3 | Mean | Standard deviation | Variation coefficient (%) |
|---|---|---|---|---|---|---|---|
| 287 | Exact mass | 287.0959 | 287.0959 | 287.0885 | 287.093433 | | |
| | Intensity | 1.04E+06 | 7.95E+05 | 7.31E+05 | 854100 | 160717.547 | 18.8 |
| 289 | Exact mass | 289.1106 | 289.1106 | 289.1106 | 289.1106 | | |
| | Intensity | 3.36E+05 | 2.92E+05 | 2.66E+05 | 297933.333 | 35078.3884 | 11.8 |
| 291 | Exact mass | 291.125 | 291.125 | 291.125 | 291.125 | | |
| | Intensity | 4.72E+05 | 4.20E+05 | 3.82E+05 | 424433.333 | 45202.3598 | 10.7 |
| 303 | Exact mass | 303.0922 | 303.0922 | 303.0922 | 303.0922 | | |
| | Intensity | 9.32E+06 | 7.74E+06 | 6.72E+06 | 7926333.33 | 1313840.68 | 16.6 |
| 317 | Exact mass | 317.1085 | 317.1085 | 317.1085 | 317.1085 | | |
| | Intensity | 6.64E+06 | 5.68E+06 | 5.02E+06 | 5781333.33 | 813967.035 | 14.1 |
| 319 | Exact mass | 319.0854 | 319.0854 | 319.0854 | 319.0854 | | |
| | Intensity | 2.79E+06 | 2.55E+06 | 2.33E+06 | 2559666.67 | 229072.769 | 8.9 |
| 331 | Exact mass | 331.1239 | 331.1239 | 331.1239 | 331.1239 | | |
| | Intensity | 1.29E+07 | 1.24E+07 | 1.13E+07 | 12230000 | 805791.536 | 6.6 |
| 333 | Exact mass | 333.1042 | 333.1042 | 333.1042 | 333.1042 | | |
| | Intensity | 1.74E+06 | 1.57E+06 | 1.46E+06 | 1590000 | 141859.085 | 8.9 |
| 347 | Exact mass | 347.1233 | 347.1233 | 347.1233 | 347.1233 | | |
| | Intensity | 1.15E+06 | 1.02E+06 | 9.47E+05 | 1039800 | 101844.587 | 9.8 |
| 409 | Exact mass | 409.1469 | 409.1469 | 409.1469 | 409.1469 | | |
| | Intensity | 5.20E+05 | 4.71E+05 | 4.32E+05 | 474133.333 | 44361.7328 | 9.4 |
| 493 | Exact mass | 493.2043 | 493.2043 | 493.2043 | 493.2043 | | |
| | Intensity | 5.84E+06 | 5.27E+06 | 4.73E+06 | 5280666.67 | 555591.877 | 10.5 |
| 535 | Exact mass | 535.2236 | 535.2236 | 535.2236 | 535.2236 | | |
| | Intensity | 4.79E+06 | 4.06E+06 | 3.68E+06 | 4175000 | 566633.038 | 13.6 |
| 536 | Exact mass | 536.2235 | 536.2235 | 536.2235 | 536.2235 | | |

| | | | | | | | |
|---|---|---|---|---|---|---|---|
| | Intensity | 1.09E+06 | 9.67E+05 | 9.24E+05 | 992966.667 | 85075.8681 | 8.6 |
| 577 | Exact mass | 577.2186 | 577.2186 | 577.2186 | 577.2186 | | |
| | Intensity | 1.72E+05 | 1.49E+05 | 1.35E+05 | 151966.667 | 18788.6491 | 12.4 |
| 579 | Exact mass | 579.2334 | 579.2334 | 579.2334 | 579.2334 | | |
| | Intensity | 1.12E+06 | 1.00E+06 | 9.12E+05 | 1012533.33 | 106021.947 | 10.5 |
| 581 | Exact mass | 581.2728 | 581.2728 | 581.2623 | 581.2693 | | |
| | Intensity | 3.88E+05 | 3.26E+05 | 3.14E+05 | 342566.667 | 39684.5478 | 11.6 |
| 639 | Exact mass | 639.2673 | 639.2673 | 639.2562 | 639.2636 | | |
| | Intensity | 3.58E+06 | 2.92E+06 | 2.71E+06 | 3072666.67 | 455105.848 | 14.8 |
| 640 | Exact mass | 640.2717 | 640.2607 | 640.2607 | 640.264367 | | |
| | Intensity | 1.03E+06 | 8.80E+05 | 8.58E+05 | 921900 | 92547.3392 | 10.0 |
| 867 | Exact mass | 867.344 | 867.344 | 867.3312 | 867.339733 | | |
| | Intensity | 4.86E+05 | 4.08E+05 | 3.87E+05 | 427033.333 | 52010.1272 | 12.2 |

## 5) 2018 WINE - LACCASE TEST

*Table S24 : High resolution UPLC-MS for 2018 red wine – laccase oxidation test*

| $[M+H]^+$ | | 2018 laccase n1 | 2018 laccase n2 | 2018 laccase n3 | Mean | Standard deviation | Variation coefficient (%) |
|---|---|---|---|---|---|---|---|
| 287 | Exact mass | 287.0885 | 287.0885 | 287.0885 | 287.0885 | | |
| | Intensity | 5.99E+05 | 5.57E+05 | 5.25E+05 | 560200 | 37003.9187 | 6.6 |
| 289 | Exact mass | 289.1032 | 289.1032 | 289.1032 | 289.1032 | | |
| | Intensity | 2.18E+05 | 2.07E+05 | 2.00E+05 | 208366.667 | 9115.00594 | 4.4 |
| 291 | Exact mass | 291.125 | 291.125 | 291.1176 | 291.122533 | | |
| | Intensity | 2.64E+05 | 2.36E+05 | 2.26E+05 | 241966.667 | 20000.0833 | 8.3 |

| | | | | | | | |
|---|---|---|---|---|---|---|---|
| 303 | Exact mass | 303.0846 | 303.0846 | 303.0846 | 303.0846 | | |
| | Intensity | 5.43E+06 | 5.12E+06 | 4.93E+06 | 5162333.33 | 250328.451 | 4.8 |
| 317 | Exact mass | 317.1085 | 317.1007 | 317.1007 | 317.1033 | | |
| | Intensity | 3.68E+06 | 3.54E+06 | 3.49E+06 | 3569333.33 | 100818.318 | 2.8 |
| 319 | Exact mass | 319.0854 | 319.0854 | 319.0854 | 319.0854 | | |
| | Intensity | 1.37E+06 | 1.24E+06 | 1.16E+06 | 1257000 | 102122.475 | 8.1 |
| 331 | Exact mass | 331.1239 | 331.1239 | 331.1239 | 331.1239 | | |
| | Intensity | 1.06E+07 | 9.73E+06 | 8.80E+06 | 9699333.33 | 887978.791 | 9.2 |
| 333 | Exact mass | 333.1042 | 333.1042 | 333.1042 | 333.1042 | | |
| | Intensity | 1.04E+06 | 9.36E+05 | 8.59E+05 | 943733.333 | 89477.6695 | 9.5 |
| 347 | Exact mass | 347.1233 | 347.1233 | 347.1233 | 347.1233 | | |
| | Intensity | 7.18E+05 | 6.32E+05 | 5.82E+05 | 643733.333 | 68744.4786 | 10.7 |
| 409 | Exact mass | 409.1469 | 409.1469 | 409.1381 | 409.143967 | | |
| | Intensity | 3.38E+05 | 3.01E+05 | 2.78E+05 | 305800 | 30164.2172 | 9.9 |
| 493 | Exact mass | 493.2043 | 493.1945 | 493.1945 | 493.197767 | | |
| | Intensity | 3.77E+06 | 3.33E+06 | 3.47E+06 | 3522666.67 | 226294.793 | 6.4 |
| 535 | Exact mass | 535.2134 | 535.2134 | 535.2134 | 535.2134 | | |
| | Intensity | 3.35E+06 | 3.14E+06 | 3.07E+06 | 3186333.33 | 144983.907 | 4.6 |
| 536 | Exact mass | 536.2235 | 536.2235 | 536.2134 | 536.220133 | | |
| | Intensity | 7.85E+05 | 6.81E+05 | 6.81E+05 | 715733.333 | 59726.9063 | 8.3 |
| 577 | Exact mass | 577.2081 | 577.2081 | 577.2081 | 577.2081 | | |
| | Intensity | 1.19E+05 | 1.11E+05 | 1.06E+05 | 111866.667 | 6951.49864 | 6.2 |
| 579 | Exact mass | 579.2229 | 579.2229 | 579.2229 | 579.2229 | | |
| | Intensity | 7.37E+05 | 6.94E+05 | 6.85E+05 | 705000 | 27888.8867 | 4.0 |
| 581 | Exact mass | 581.2623 | 581.2623 | 581.2623 | 581.2623 | | |
| | Intensity | 2.66E+05 | 2.44E+05 | 2.36E+05 | 248666.667 | 15851.2881 | 6.4 |
| 639 | Exact mass | 639.2562 | 639.2562 | 639.2562 | 639.2562 | | |

| | | | | | | | |
|---|---|---|---|---|---|---|---|
| | Intensity | 2.69E+06 | 2.47E+06 | 2.32E+06 | 2494333.33 | 184925.21 | 7.4 |
| 640 | Exact mass | 640.2607 | 640.2607 | 640.2607 | 640.2607 | | |
| | Intensity | 8.09E+05 | 7.36E+05 | 6.87E+05 | 744266.667 | 61339.2479 | 8.2 |
| 867 | Exact mass | 867.3312 | 867.3312 | 867.3312 | 867.3312 | | |
| | Intensity | 3.48E+05 | 3.12E+05 | 2.98E+05 | 319633.333 | 25724.3724 | 8.0 |

## 6) 2018 WINE - HYDROGEN PEROXIDE TEST

*Table S25 : High resolution UPLC-MS for 2018 wine – hydrogen peroxide oxidation test*

| $[M+H]^+$ | | 2018 $H_2O_2$ n1 | 2018 $H_2O_2$ n2 | 2018 $H_2O_2$ n3 | Mean | Standard deviation | Variation coefficient (%) |
|---|---|---|---|---|---|---|---|
| 287 | Exact mass | 287.0885 | 287.0885 | 287.0885 | 287.0885 | | |
| | Intensity | 4.70E+05 | 4.24E+05 | 4.14E+05 | 436000 | 29960.1402 | 6.9 |
| 289 | Exact mass | 289.1032 | 289.1032 | 289.1032 | 289.1032 | | |
| | Intensity | 2.09E+05 | 1.94E+05 | 1.88E+05 | 196933.333 | 10864.7749 | 5.5 |
| 291 | Exact mass | 291.1176 | 291.1176 | 291.1176 | 291.1176 | | |
| | Intensity | 2.75E+05 | 2.51E+05 | 2.45E+05 | 256666.667 | 15837.0873 | 6.2 |
| 303 | Exact mass | 303.0846 | 303.0846 | 303.0846 | 303.0846 | | |
| | Intensity | 5.38E+06 | 4.95E+06 | 4.83E+06 | 5053666.67 | 291453.827 | 5.8 |
| 317 | Exact mass | 317.1007 | 317.1007 | 317.1007 | 317.1007 | | |
| | Intensity | 3.36E+06 | 3.14E+06 | 2.97E+06 | 3156333.33 | 196573.989 | 6.2 |
| 319 | Exact mass | 319.0854 | 319.0854 | 319.0854 | 319.0854 | | |
| | Intensity | 1.19E+06 | 1.11E+06 | 1.08E+06 | 1127000 | 52716.2214 | 4.7 |
| 331 | Exact mass | 331.1239 | 331.1239 | 331.1239 | 331.1239 | | |
| | Intensity | 4.95E+05 | 4.54E+05 | 4.37E+05 | 461833.333 | 30026.3773 | 6.5 |

| | | | | | | | |
|---|---|---|---|---|---|---|---|
| 333 | Exact mass | 333.0962 | 333.0962 | 333.0962 | 333.0962 | | |
| | Intensity | 9.83E+05 | 9.34E+05 | 9.14E+05 | 943500 | 35544.7605 | 3.8 |
| 347 | Exact mass | 347.1152 | 347.1152 | 347.1152 | 347.1152 | | |
| | Intensity | 1.10E+06 | 1.05E+06 | 1.01E+06 | 1050666.67 | 44185.2162 | 4.2 |
| 409 | Exact mass | 409.1381 | 409.1381 | 409.1381 | 409.1381 | | |
| | Intensity | 2.01E+05 | 1.94E+05 | 1.85E+05 | 193333.333 | 8203.25139 | 4.2 |
| 493 | Exact mass | 493.1945 | 493.1945 | 493.1945 | 493.1945 | | |
| | Intensity | 2.13E+05 | 1.99E+05 | 1.89E+05 | 200033.333 | 12027.1914 | 6.0 |
| 535 | Exact mass | 535.2134 | 535.2134 | 535.2134 | 535.2134 | | |
| | Intensity | 1.96E+05 | 1.84E+05 | 1.76E+05 | 185166.667 | 9985.15565 | 5.4 |
| 536 | Exact mass | 536.2134 | 536.2134 | 536.2134 | 536.2134 | | |
| | Intensity | 5.56E+04 | 5.21E+04 | 5.06E+04 | 52756.6667 | 2537.76148 | 4.8 |
| 577 | Exact mass | 577.2081 | 577.2081 | 577.2081 | 577.2081 | | |
| | Intensity | 1.05E+05 | 9.97E+04 | 9.38E+04 | 99393.3333 | 5477.31078 | 5.5 |
| 579 | Exact mass | 579.2229 | 579.2229 | 579.2229 | 579.2229 | | |
| | Intensity | 7.22E+05 | 6.74E+05 | 6.54E+05 | 683466.667 | 34671.0734 | 5.1 |
| 581 | Exact mass | 581.2623 | 581.2623 | 581.2623 | 581.2623 | | |
| | Intensity | 3.16E+05 | 2.94E+05 | 2.88E+05 | 298933.333 | 14688.8847 | 4.9 |
| 639 | Exact mass | 639.2452 | 639.2452 | 639.2452 | 639.2452 | | |
| | Intensity | 2.00E+05 | 1.92E+05 | 1.84E+05 | 192000 | 8107.40402 | 4.2 |
| 640 | Exact mass | 640.2496 | 640.2496 | 640.2496 | 640.2496 | | |
| | Intensity | 7.15E+04 | 6.95E+04 | 6.34E+04 | 68120 | 4179.38991 | 6.1 |
| 867 | Exact mass | 867.3312 | 867.3312 | 867.3312 | 867.3312 | | |
| | Intensity | 2.80E+05 | 2.61E+05 | 2.52E+05 | 264533.333 | 14368.484 | 5.4 |
